# USP21 promotes self-renewal and tumorigenicity of mesenchymal glioblastoma stem cells by deubiquitinating and stabilizing FOXD1

**DOI:** 10.1038/s41419-022-05163-3

**Published:** 2022-08-16

**Authors:** Qixiang Zhang, Zhengxin Chen, Qikai Tang, Zhangjie Wang, Jiacheng Lu, Yongping You, Huibo Wang

**Affiliations:** Department of Neurosurgery, First Affiliated Hospital of Nanjing Medical University, Nanjing, 210029 China

**Keywords:** Cancer stem cells, CNS cancer

## Abstract

Recent studies suggest that Forkhead box D1 (FOXD1) plays an indispensable role in maintaining the mesenchymal (MES) properties of glioblastoma (GBM) stem cells (GSCs). Thus, understanding the mechanisms that control FOXD1 protein expression is critical for guiding GBM treatment, particularly in patients with therapy-resistant MES subtypes. In this study, we identify the ubiquitin-specific peptidase 21 (USP21) as a critical FOXD1 deubiquitinase in MES GSCs. We find that USP21 directly interacts with and stabilizes FOXD1 by reverting its proteolytic ubiquitination. Silencing of USP21 enhances polyubiquitination of FOXD1, promotes its proteasomal degradation, and ultimately attenuates MES identity in GSCs, while these effects could be largely restored by reintroduction of FOXD1. Remarkably, we show that disulfiram, a repurposed drug that could block the enzymatic activities of USP21, suppresses GSC tumorigenicity in MES GSC-derived GBM xenograft model. Additionally, we demonstrate that USP21 is overexpressed and positively correlated with FOXD1 protein levels in GBM tissues, and its expression is inversely correlated with patient survival. Collectively, our work reveals that USP21 maintains MES identity by antagonizing FOXD1 ubiquitination and degradation, suggesting that USP21 is a potential therapeutic target for the MES subtype of GBM.

## Introduction

Glioblastoma (GBM) is one of the most deadly and recalcitrant solid malignancies in adults. Current standard-of-care for patients with GBM involves maximal feasible surgical resection followed by radiation and temozolomide-based chemotherapy. Unfortunately, the therapeutic efficacy of these treatment options remains limited, and the majority of patients with GBM experience tumor recurrence after initial treatment [1]. GBM is distinguished by a high degree of intratumoral heterogeneity and has been subdivided into at least four molecular subtypes with distinct genetic alterations: proneural (PN), neural (NL), classical (CL) and mesenchymal (MES) [2]. Among them, the MES subtype is the most aggressive due to its highly invasive and therapy-resistant nature [3, 4]. GBM stem cells (GSCs) are a highly heterogeneous population that resides at the apex of cellular hierarchies, forming a reservoir of self-sustaining cells capable of initiating, maintaining, and repopulating tumor mass, which are known culprits of therapy resistance and disease recurrence [5]. GSCs can be classified as PN or MES subtypes based on their gene expression profiles and distinct biological properties. Compared with PN GSCs, MES GSCs manifest markedly resistance to radiochemotherapies. Besides, PN GSCs can transform into MES GSCs both during the natural evolution of GBM and in response to extrinsic stimuli [6].

Forkhead box D1 (FOXD1) is a core transcription factor member of FOX family and located on chromosome 5q12 [7]. The FOXD1 protein plays a vital role in physiological development during embryogenesis. Accumulating evidence has demonstrated that FOXD1 is associated with tumorigenesis and cancer progression, such as therapeutic resistance and cancer metastasis, and serves as a prognostic biomarker and a promising target in several types of cancer [8–12]. Notably, it has been recently reported that FOXD1 is critical for the maintenance of MES GSCs, as well as the tumorigenicity of this subtype of GBM [13]. However, the mechanism of FOXD1 overexpression in GSCs remains unclear.

Deubiquitylating enzymes (DUBs) are a large group of proteases that remove the ubiquitin chains from ubiquitylated substrates to antagonize the modification mediated by E3 ubiquitin ligases, which is is important for diverse biological functions such as cell cycle and division, DNA transcription and repair, differentiation and development, immune response, nerve and muscle degeneration, and cell apoptosis [14]. Numerous studies have shown that DUBs function as critical controllers of various signal pathways involved in many types of cancer and other diseases, and are emerging as potential biomarkers and drug targets. Therefore, exploring the role of DUB and their downstream effectors will provide new insights into the molecular basis of cancer onset and guide development of novel therapies. Thus far, approximately 100 DUBs can be classified into seven families: ubiquitin-specific protease (USP), JAB1/MPN/Mov34 metalloenzyme (JAMM), ovarian tumor protease (OTU), Josephine, and JAB1/MPN + (MJP), recently discovered UbC-terminal hydrolase (UCH), and two new types of DUB (MINDY) containing MIU, Ub peptidase 1 (ZUP1) containing zinc fingers [15, 16]. Among these DUBs, the USPs form the largest subfamily, which is composed of more than 60 members.

USP21 belongs to the USP family and has been identified as an interacting partner of multiple substrate proteins, Nanog, H2A, RIPK1, GATA3, RIG-1, and Tip5 [17–22]. Accumulating evidence has shown that USP21 exert oncogenic functions in a variety of human cancers [18, 23–28]. However, whether USP21 plays a role in GBM biology remains unknown. In this study, we identify USP21 as a novel upstream regulator which interacts with and stabilizes FOXD1 and show that genetic or pharmacological inhibition of USP21 abrogates MES GSC-derived tumor growth in vitro and in vivo by antagonizing FOXD1 ubiquitination and degradation. Our study reveals that USP21-FOXD1 axis functions as an important regulatory mechanism of the maintenance of MES identity in GSCs and provides a potential therapeutic approach in the management of the MES subtype of GBM.

## Materials and methods

### Cell cultures

The human GBM U87, U251, T98G, LN229 cell lines, and the human embryonic kidney HEK293T cell line were purchased from ATCC. These cell lines were cultured in DMEM with 10% FBS. NHAs cell line was purchased from ScienCell Research Laboratory and cultured in astrocyte growth medium supplemented with rhEGF, insulin, ascorbic acid, GA-1000, l-glutamine, and 5% FBS. GBMs cells were isolated from primary GBM tumors or patient-derived GBM xenografts [29]. The cells were then recovered in DMEM/F12 medium supplemented with B27 (Gibco, catalog 17504044), bFGF, and EGF (20 ng/ml each). MES GSCs was enriched for CD44, while PN GSCs were enriched for OLIG2 by fluorescence-activated cell sorting (FACS). Only early-passages GSCs were used for research. All the cells were routinely tested for mycoplasma contamination bimonthly using MycoAlert PLUS Kits (Lonza).

### Human tissue samples

Four GBM specimens were obtained from the Department of Neurosurgery, First Affiliated Hospital of Nanjing Medical University. A total of 138 GBM tissues were obtained from the Department of Neurosurgery, Second and Fourth Affiliated Hospitals of Harbin Medical University [30]. Specifically, 91 samples exhibited high ALDH1A3 expression (MES GBM marker).

### Transfection

USP21 and control short hairpin RNA (shRNA) were purchased from Sigma–Aldrich. The targeting sequence for shUSP21#1 was 5′-TCACTAAGGAAGAAGAGCT-3′. The targeting sequence for shUSP21#2 was 5′-AACCTAATGTGGAAACGTT-3′. Flag-USP21, Myc-FOXD1, truncated mutants of USP21 and FOXD1, HA-Ub, HA-Ub-K0, HA-Ub-K11, HA-Ub-K27, HA-Ub-K48, and HA-Ub-K63 overexpression plasmids were purchased from GeneChem. All transfections used Lipofectamine 3000 (Invitrogen, catalog L3000150) were performed following the manufacturer’s instructions. Cells transfected with Lipofectamine 3000 for stable cell lines were screened by puromycin.

### siRNA library screening

The siRNA library for human DUB customized from Ribobio was used to screen human deubiquitinating enzymes. HEK293T cells were added to the 96-wells plate, then transfected with siRNAs. After 48 h, the cells were lysed, and the endogenous FOXD1 protein levels were analyzed by immunoblot (IB).

### IB

Following antibodies were used for IB analysis. Anti-USP21 (catalog MA5-34953), anti-FOXD1 (catalog PA5-27142) were purchased from Invitrogen. Anti-TAZ (catalog ab84927), anti-C/EBPβ (catalog ab32358), anti-c-MET (catalog ab74217), anti-ALDH1A3 (catalog ab129815), anti-OLIG2 (catalog ab109186), anti-Flag (catalog ab205606), anti-Myc (catalog ab32), anti-HA (catalog 236632), and GAPDH (catalog ab8245) were purchased from Abcam. Anti-CD44 (catalog 3570), anti-p-STAT3 (catalog 9145), anti-STAT3 (catalog 9139), anti-mouse IgG (catalog 5415), anti-rabbit IgG (catalog 3900), and anti-GST (catalog 2624) were purchased from Cell Signaling Technology.

Samples or cells with indicated treatments were lysed with cell lysis buffer (Beyotime, catalog P0013) with protease Cocktail Inhibitor (MCE, catalog HY-K0010) at 4 °C for 30 minutes. The lysates were centrifugated at 12,000 rpm for 20 min. Cell lysates were subjected to SDS-PAGE, transferred onto a polyvinylidenedifluoride membrane (Roche, catalog 03010040001), followed by incubation overnight at 4 °C with primary antibodies. The membrane was incubated with horseradish peroxidase (HRP)-conjugated secondary antibodies for 2 h. Proteins were then detected with the enhanced chemiluminescence methods.

### Immunofluorescence (IF) and immunohistochemistry (IHC)

Following antibodies were used for IF and IHC analysis. Anti-USP21 (catalog MA5-34953), anti-FOXD1 (catalog PA5-35145) were purchased from Invitrogen. Anti-ALDH1A3 (catalog ab129815) was purchased from Abcam. Anti-CD44 (catalog 3570) was purchased from Cell Signaling Technology.

GSCs were fixed in 4% formaldehyde for IF staining, permeated with 0.25% Triton X-100, and then blocked with 1% BSA at room temperature for 1 h. The cells were probed with the primary antibody. After washing by PBS-T, cells were combined with the Alexa Fluor 488 (abcam, catalog ab150113) or Alexa Fluor 647 (abcam, catalog ab150079) labeled secondary antibody.We used a mounting medium containing DAPI (abcam, catalog ab104139). The samples were observed through a confocal laser scanning microscope. For IHC staining, the slides were handled as before [29]. If <10% of the cells in the tumor area were stained, the result would be classified as negative. If the staining were 10% to 100%, the result would be positive. The percentage of positive tumor cells per slide (10–100%) multiplied by the main intensity pattern of staining (1, weak; 2, medium; 3, strong).

### RNA isolation and quantitative real-time PCR

RNA was extracted by FastPure Cell/Tissue Total RNA Isolation Kit V2 (Vazyme, catalog RC112). The PrimeScript RT Master Mix (TaKaRa, catalog 360 A) was used to reverse RNA transcription to cDNA. SYBR Green (Vazyme, catalog Q141) was used in real-time PCR. The forward and reverse PCR primers for USP21 were 5′-ACTGGGGATACGATGGCTGA-3′ and 5′-ACAGGCTGGACCCACAATC-3′. The forward and reverse PCR primers for FOXD1 were 5′-TCTCGTCTTGGTGGTTCGGT-3′ and 5′-CTGTAGCATAGGTCGGCTTTG-3′. GAPDH mRNAs were used as the internal control. The forward and reverse PCR primers for GAPDH were 5′-TGTGTCCGTCGTGGATCTGA-3′ and 5′-CACCACCTTCTTGATGTCATCATAC-3′.

### Co-immunoprecipitation assay

Lysis buffer (Beyotime, catalog P0013), which contained a protease inhibitor cocktail (MCE, catalog HY-K0010), was used for lysis. The indicated antibodies were used in cell lysates for immunoprecipitation at 4 °C overnight and then incubated with protein A/G (Beyotime, catalog P2055) at 4 °C for 3 h. Then the cell lysates were washed with lysis buffer four times and then analyzed by IB.

### GST pull-down assay

Bacterially expressed GST, GST-FOXD1 was incubated with Flag-USP21WT or Flag-USP21C221A lysed from HEK293T cells using GST Protein Interaction Pull-Down Kit (Thermo Scientific, catalog 21516). The complex was then analyzed by Coomassie Brilliant Blue.

### Determination of protein half-life

Specific cells treated with protein synthesis inhibitor CHX (100 μg/ml; Sigma–Aldrich) were lysed at indicated time points. The lysates were analyzed by IB.

### Neurosphere formation assay and limiting dilution test

In the neurosphere formation assay, the dissociated single cells were plated at a density of 1 cell/μl. After 7 days, the spheroids formed were analyzed. For extreme limiting dilution experiments, GSCs with specific treatment were separated into individual cells and then seeded in 96-well plates at a density of 1, 5, 10, 20, or 50 cells per well. Formations of tumor balls were checked 7 days later.

### Intracranial xenograft tumor models and treatments

For tumorigenicity studies, 8-weeks-old male BALB/c nude mice were randomly divided into the corresponding group (*n* = 10). For modeling, a stereotaxic instrument and microsyringe were used to implant 1 × 10^5^ luciferase-expressing GSCs into the right caudate nucleus of immunocompromised mice (coordinates: 2 mm anterior, 2 mm lateral, 3 mm depth from the dura). All mice were monitored daily for the development of neurological symptoms. We performed in vivo imaging of the mice with orthotopic tumor xenografts using the IVIS Lumina III in vivo imaging system. The mice were humanely euthanized 2 to 10 weeks after implantation, and their brains were harvested, paraffin-embedded, stained with H&E to confirm the presence of tumor, and subjected toimmunohistochemical staining.

For testing in vivo inhibition effect of USP21 inhibitor disulfiram, 1 × 10^5^ MES GSCs were implanted intracranially into individual mice. ALZET micro-osmotic pumps (DURECT Corp.) and infusion apparatus were implanted into tumor-bearing mice, and CED of either disulfiram (50 mg/kg) or vehicle was initiated. Tumor growth was monitored by bioluminescent imaging and mice were maintained until the manifestation of neurologic signs.

### Datasets

All datasets used in this study were available to the public. Gene expression data and corresponding clinical data from glioma patients were downloaded from the Chinese Glioma Genome Atlas (CGGA) (http://www.cgga.org.cn/).

### Statistical analysis

In this study, we used GraphPadPrism 8 to analyze the data. Each experiment was repeated at least three times independently. The experimental data were expressed as the mean ± SD. The data analysis used in the article included two-tailed Student’s *t*-tests, two or three-way ANOVAs. A log-rank (Mantel-Cox) analysis was used to determine statistical significance of Kaplan–Meier survival curves. *P* < 0.05 was considered a significant difference.

### Study approval

All tumor collection and analysis was approved by the the ethics committees of Nanjing Medical University and Harbin Medical University. Informed consent was obtained from all individual participants. All animal experiments were conducted with the approval of IACUC of Nanjing Medical University and in conformity with the Guide for the Care and Use of Laboratory Animals (National Academies Press, 2011).

## Results

### USP21 maintains the stability of FOXD1 protein

To assess whether FOXD1 is regulated by the ubiquitin-proteasomal system (UPS), we treated HEK293T cells and NHAs with cycloheximide (CHX), an inhibitor of de novo protein synthesis, and chased the protein levels of FOXD1. We noted that FOXD1 was gradually degraded, and only half of its original protein levels could be detected within 6 h after CHX treatment. Moreover, we detected a significant increase in FOXD1 protein levels in HEK293T cellsafter treatment with the proteasome inhibitor MG132 (Supplementary Fig. 1a). These data suggest that FOXD1 could be degraded via UPS.

**Fig. 1:**
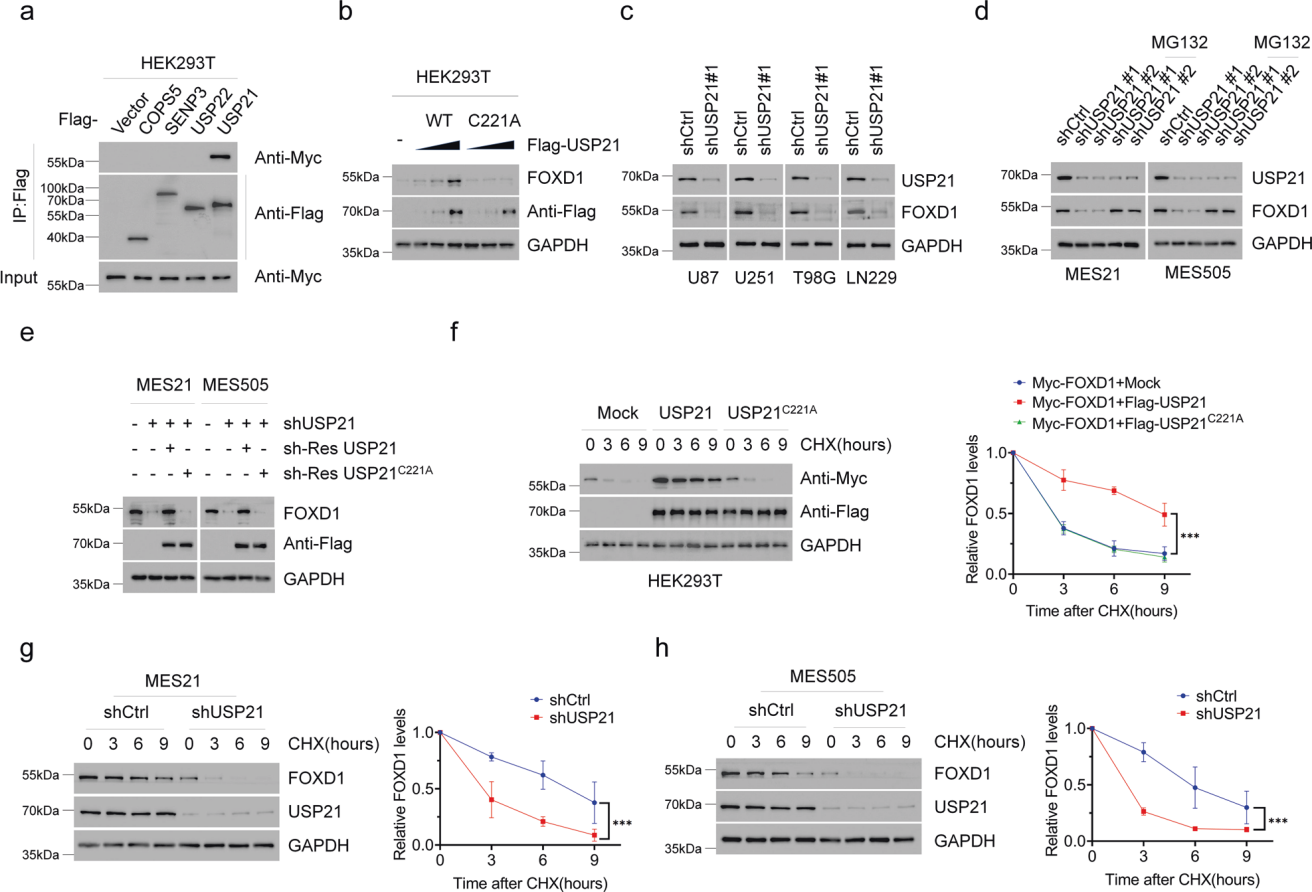
USP21 maintains FOXD1 stability. **a** Co-IP showing that FOXD1 was physically conjugated with USP21 among the predicated proteins(COPS5, SENP3, USP22, USP21). Anti-Flag antibody was used to bind Flag-tagged USP21 WT or C221A. Anti-Myc antibody was used to bind Myc-tagged FOXD1. **b** Western blotting showing that transiently transfecting of USP21-overexpressing plasmid altered the expression of FOXD1 in a dose-dependent manner. **c** Western blotting showing that knockdown of USP21 attenuated the protein expression of FOXD1 in U87, U251, T98G and LN229 GBM cell lines. **d** Western blotting shows that knockdown of USP21 attenuated the expression of FOXD1 in MES 21 and 505 GSCs, whereas treatment with MG-132 (20 μM) abolished the effect of the knockdown of USP21 in MES 21 and 505 GSCs thus increasing the expression of FOXD1. **e** Western blotting showing that the overexpression of an shRNA-resistant WT, but not C221A mutant, USP21 altered the effect of the knockdown of USP21 in MES 21 and 505 GSCs thus increasing the expression of FOXD1. **f** Western blotting showing that the overexpression of wild-type USP21 (USP21 WT) but not the catalytically inactive USP21 mutant (USP21 C221A) stabilized FOXD1. ***P < 0.001. **g**, **h** Western blotting showing that the knockdown of USP21 in MES 21 (**g**) and 505 (**h**) GSCs resulted in accelerated degradation of FOXD1.*** *P* < 0.001.

To screen out possible upstream regulatory DUBs of FOXD1, we added the siDUBs library into the HEK293T cells and detected changes in the protein levels of FOXD1. We identified four potential DUBs (COPS5, SENP6, USP22, and USP21) as major FOXD1-associated proteins (Supplementary Fig. 1b, c). We then expressed each of these four DUBs in HEK293T cells and found that only USP21, but not COPS5, SENP6 or USP22, was able to directly interact with the endogenous FOXD1 (Fig. 1a). Thus, USP21 stood out as a potential FOXD1-interacting protein.

Next, we transfected Flag-USP21 WT or a catalytically inactive mutant USP21 C221A into HEK293T cells. We found that USP21WT but not USP21 C221A could remarkably enhance the FOXD1 protein levels in a dose-dependent manner (Fig. 1b). By contrast, knockdown of USP21 in four GBM cell lines markedly reduced FOXD1 protein levels, but had no effect on its mRNA levels (Fig. 1c and Supplementary Fig. 1d). Moreover, we utilized MES cell surface marker CD44 to enrich two GSCs (MES 21 and 505 GSCs) from patient-derived GBM cells and observed that USP21 was highly expressed in MES 21 and 505 GSCs (Supplementary Fig. 1e). Upon silencing with USP21 shRNA, FOXD1 protein, but not mRNA, expression was drastically decreased in two MES GSCs, which could be almost completely reversed by addition of the proteasome inhibitor MG132 or overexpression of an shRNA-resistant WT, but not C221A mutant, USP21 (Fig. 1d, e and Supplementary Fig. 1f, g). To determine whether USP21 could affect the stability of FOXD1 per se, we used CHX to cease protein synthesis and detect the FOXD1 protein levels after manipulation of USP21. Ectopic expression of USP21 WT, but not the C221A mutant, led to a marked increase in the stability of ectopically expressed FOXD1 protein in HEK293T cells (Fig. 1f), whereas knockdown of USP21 expression in MES 21 and 505 GSCs resulted in destabilization of the FOXD1 protein (Fig. 1g, h). Together, these findings suggest that USP21 could regulate the stability of FOXD1 protein.

### USP21 interacts with FOXD1

**Fig. 2:**
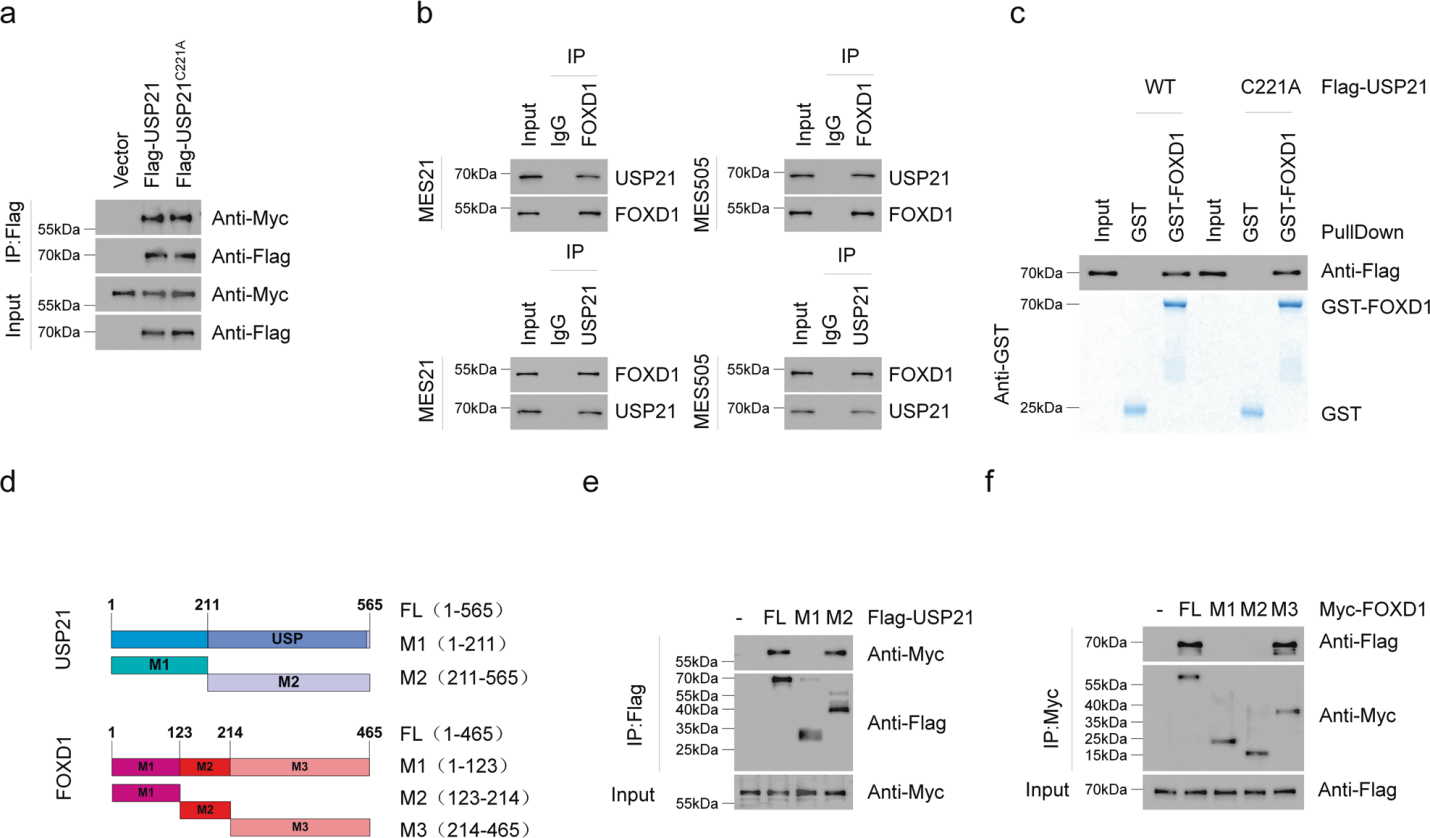
USP21 directly interacts with FOXD1. **a** Co-IP showing FOXD1 interaction with USP21 WT and C221A mutant. Anti-Flag antibody was used to bind Flag-tagged USP21 WT or C221A. Anti-Myc antibody was used to bind Myc-tagged FOXD1. **b** Co-IP validation of the interaction between USP21 and FOXD1 in MES 21 and 505 GSCs. **c** The GST-Pulldown assay showing the direct interaction between USP21 and FOXD1. **d** Schematic structures of USP21 and FOXD1, together with their truncated mutants. **e** Co-IP showing that the C terminal of USP21 is essential for the interaction with FOXD1. **f** Co-IP shows that the C terminal of FOXD1 is indispensable for the interaction with USP21.

To further confirm the association between USP21 and FOXD1, we carried out co-immunoprecipitation (Co-IP) experiments. The results showed that Myc-FOXD1 could be readily detected in either Flag-USP21 WT or Flag-USP21 C221A immunoprecipitates in HEK293T cells (Fig. 2a). Moreover, reciprocal Co-IP assays were performed in MES 21 and 505 GSCs, confirming a physical association between endogenous USP21 and FOXD1 (Fig. 2b). Furthermore, we conducted in vitro GST pull-down assay by mixing purified GST-FOXD1 with purified recombinant protein Flag-USP21WT or Flag-USP21C221A. Either USP21WT or its C221A mutant was able to bind to immobilized GST-FOXD1 but not to GST alone, thus confirming that there is a direct interaction between the two proteins (Fig. 2c).

FOXD1 consists of a DNA-binding domain, an NH_2_-terminal domain, and a COOH-terminal domain. USP21 is composed of an NH_2_-terminal domain, and a C terminal USP domain. To map the minimal essential domains required for their interaction, a series of deletion mutants of FOXD1 together with Flag-USP21 constructs were co-transfected into HEK293T cells (Fig. 2d). Co-IP analysis showed that the COOH-terminal domain of FOXD1 (amino acids 214-465) is essential for its interaction with USP21 (amino acids 211-565) (Fig. 2e, f). Together, these results indicate that USP21 directly interacts with FOXD1 in a manner that is independent of its DUB activity.

### USP21 deubiquitinates FOXD1

**Fig. 3:**
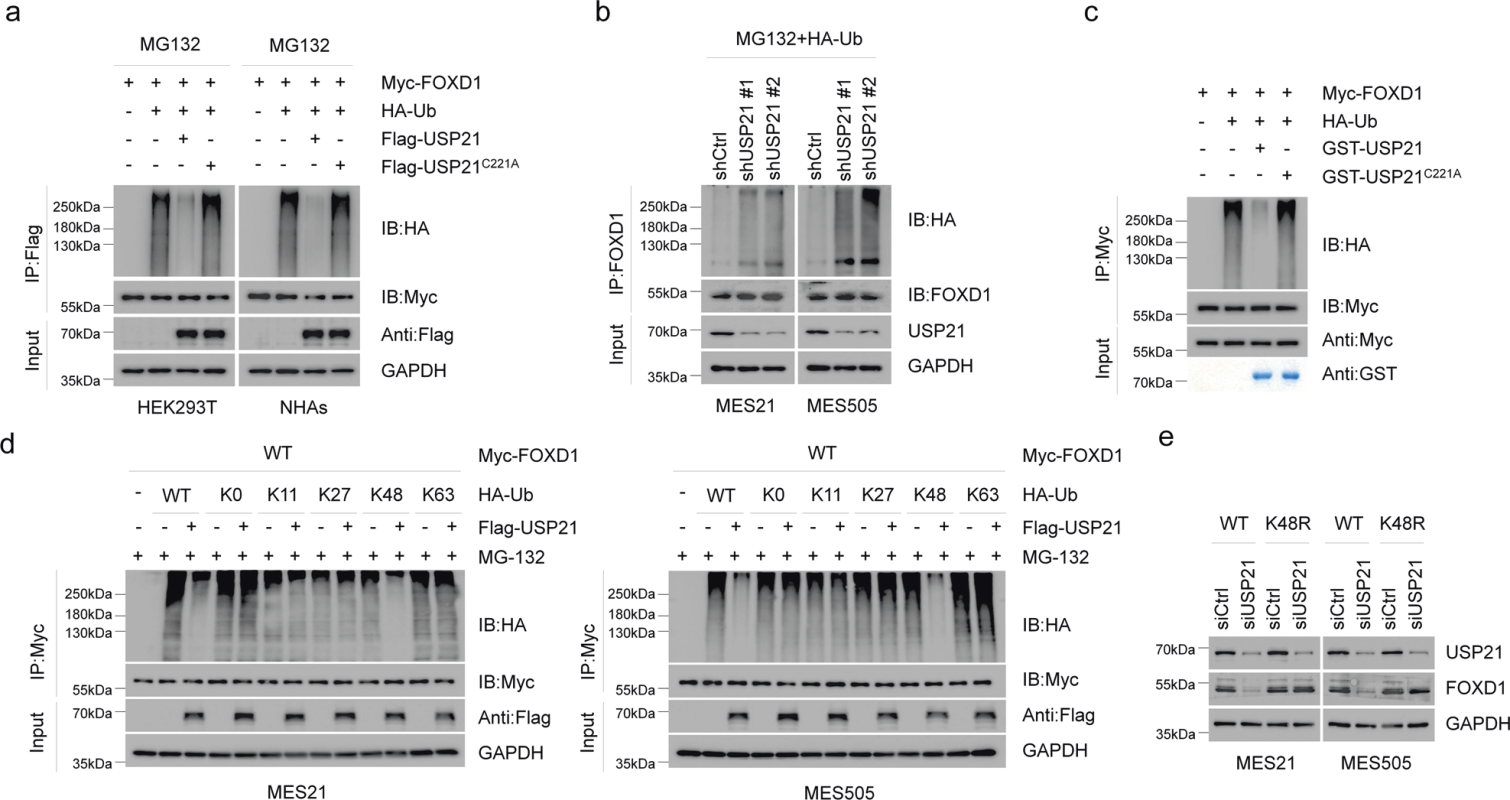
USP21 deubiquitinates FOXD1. **a** Co-IP showing that USP21 WT, but not C221A, reduced polyubiquitinated FOXD1. Anti-HA antibody was used to bind HA-tagged Ub to indicate ubiquitination. **b** Co-IP showing that the knockdown of USP21 increased accumulation of polyubiquitinated FOXD1 in MES 21 and 505 GSCs. Anti-HA antibody was used to bind HA-tagged Ub to indicate ubiquitination. **c** GST-Pulldown assay showing that FOXD1 is a direct deubiquitinated substrate of USP21. **d** Co-IP showing that USP21 efficiently disassembled K48-linked polyubiquitylation of FOXD1 but had no significant effect on monoubiquitylation or the K11, K27, K63-linked polyubiquitylation of FOXD1. Anti-HA antibody was used to bind HA-tagged Ub to indicate ubiquitination. **e** Western blotting showing that enforced expression of Lys48R ubiquitin attenuated USP21 depletion–induced FOXD1 downregulation.

To examine whether USP21 catalyzes the deubiquitination of FOXD1,we transduced Flag-USP21 WT or Flag-USP21 C221A into HEK293T cells and NHAs cells, and found that WT, but not C221A USP21, specifically removed FOXD1 ubiquitylation (Fig. 3a). By contrast, silencing of USP21 by two independent shRNAs enhanced FOXD1 ubiquitylation in MES 21 and 505 GSCs (Fig. 3b). To prove that FOXD1 is a direct deubiquitinated substrate of USP21, we purified Flag-USP21WT, Flag-USP21C221A, and ubiquitylated FOXD1, and incubated them under cell-free conditions. Purified Flag-USP21WT, but not Flag-USP21C221A, which is still able to interact with FOXD1, reduced the polyubiquitin chain of FOXD1 in vitro (Fig. 3c), suggesting that USP21 could directly deubiquitylate FOXD1. Moreover, we examined the types of ubiquitin linkage using in the presence of different ubiquitin monomers. As shown in Fig. 3d, USP21 efficiently disassembled K48-linked polyubiquitylation of FOXD1 but had no significant effect on monoubiquitylation or the K11, K27, K63-linked polyubiquitylation of FOXD1. To further verify that K48-linked polyubiquitination is essential for USP21-mediated FOXD1 degradation, we expressed a K48-resistant (K48R) form of ubiquitin into MES 21 and 505 GSCs depleted of USP21. As anticipated, ectopic expression of K48R ubiquitin attenuated downregulation of FOXD1 induced by USP21 knockdown (Fig. 3e). Collectively, these results suggest that USP21 deubiquitylates K48-linked polyubiquitylation of FOXD1.

### USP21 regulates self-renewal and tumorigenicityof MES GSCs

**Fig. 4:**
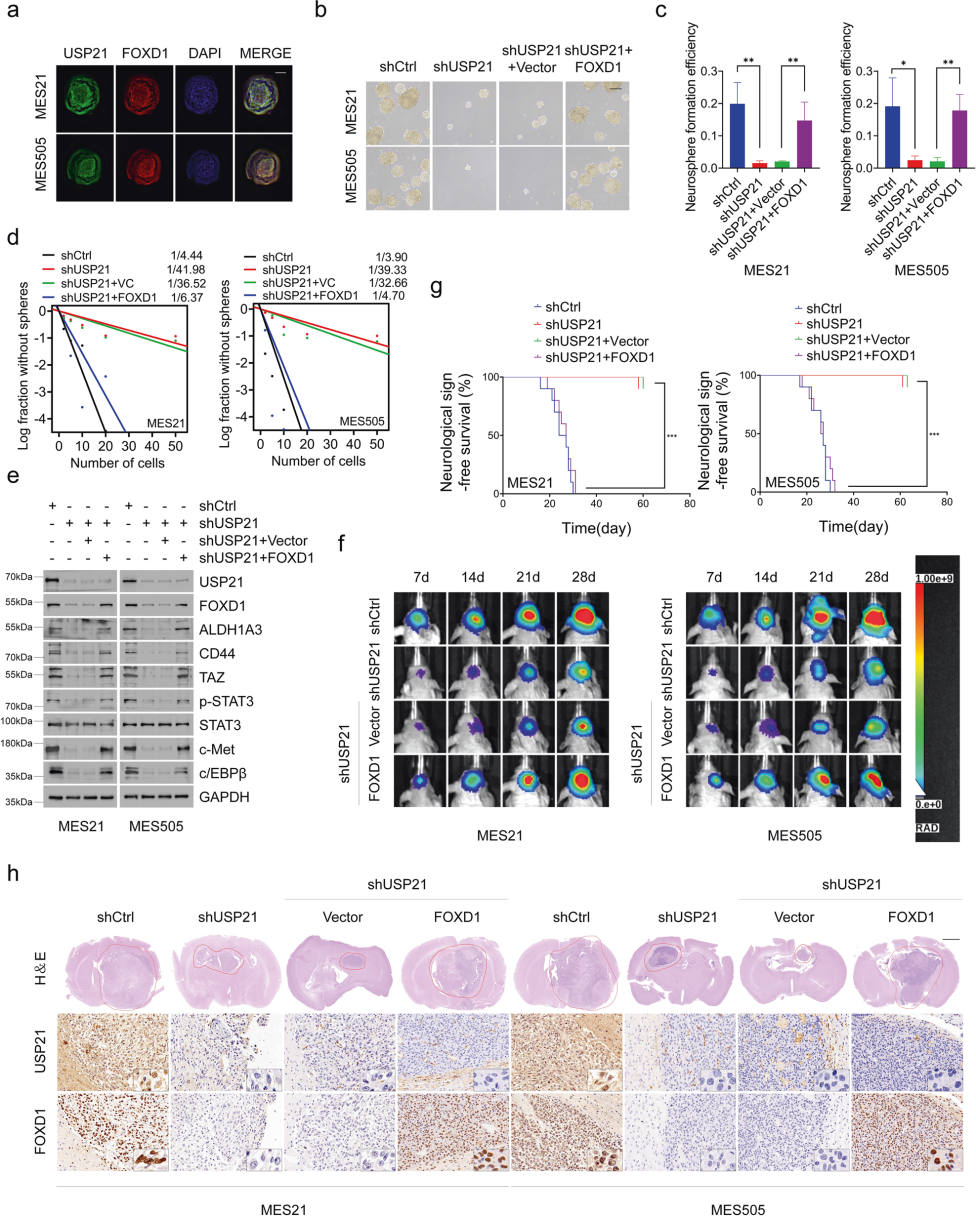
USP21 regulates self-renewal and tumorigenicity of MES GSCs. **a** Immunofluorescence assays showing that USP21 colocalizes with FOXD1 in the nuclei of MES 21 and 505 GSCs. Scale bar: 30 μm (**b**, **c**). Primary neurosphere formation showing that the silencing of USP21 considerably attenuated cell growth, and the effect of USP21-knockdown could be largely rescued by FOXD1. Right panel (**c**) showing the quantification of neurosphere formation efficiency (spheres/cells plated). Data are shown as mean ± S.D., *n* = 3, *P < 0.05, **P < 0.01, Student’s *t*-test. **d** In vitro limiting dilution sphere-forming frequency showing that the knockdown of USP21 remarkably reduced the tumorsphere formation frequency of MES21 and 505 GSCs, and the effect of USP21-knockdown could be rescued by FOXD1. Stem cell frequencies were estimated as the ratio 1/x with the upper and lower 95% confidence intervals, where 1 = stem cell and x = all cells. *P < 0.05. **e** Western blotting showing that the USP21/FOXD1 axis has great importance in the maintenance of core MES-specific markers, including ALDH1A3, CD44, C/EBPβ, TAZ, phosphorylated STAT3 (p-STAT3), and c-MET. **f** Representative bioluminescent images showing that USP21-knockdown MES 21 and 505 GSCs had lower tumorigenicity abilities than control GSCs while the tumorigenicity abilities could be rescued by FOXD1. *n* = 10. **g** Kaplan–Meier survival curves showing that mice bearing xenograft tumors formed by USP21-knockdown GSCs had a longer lifespan than control GSCs, and the survival time in mice intracranially injected with USP21-depleted GSCs significantly decreased after FOXD1 overexpression. *n* = 10, ***P < 0.001, Log-rank test. **h** Representative H&E and IHC images showing that the xenografts carrying USP21 shRNA GSCs displayed restricted tumor growth, whereas this effect could be reversed by overexpression of FOXD1.

Next, we used immunofluorescence to determine the cellular localization of USP21 and FOXD1. The results showed that USP21 colocalizes with FOXD1 in the nuclei of MES 21 and 505 GSCs (Fig. 4a). Given this finding, coupled with the role of USP21 in regulating FOXD1 stability, we wondered whether USP21 plays a role in regulating the biological behaviors of GSCs. To that end, we used a distinct Dox-inducible USP21 short hairpin RNA (shRNA) and effectively inhibited the high-level expression of endogenous USP21 in luciferase-labeled MES 21 and 505 GSCs (Supplementary Fig. 2a). Limiting dilution assays showed that depletion of USP21 dramatically reduced the tumorsphere formation frequency of two GSCs (Fig. 4b–d). Moreover, immunoblotting of dissociated tumorspheres revealed that USP21 knockdown decreased the expression of FOXD1 and the core MES GSC markers including ALDH1A3, CD44, TAZ, p-STAT3, c-MET and c-EBPβ (Fig. 4e). Furthermore, exogenous expression of FOXD1 in USP21-depleted MES 21 and 505 GSCs led to enhanced sphere-forming ability and expression of those MES GSC markers (Fig. 4b–e). These data suggest that USP21 regulates the MES properties of GSCs.

To explore the role of USP21 in MES GBM tumorigenicity, we established in vivo MES GBM nude mouse model. The results showed that the xenografts carrying control shRNA GSCs exhibited more rapid tumor formation, while, in stark contrast, tumor growth was significantly repressed in the xenografts carrying GSCs with USP21 depletion (Fig. 4f). Subsequently, the mice were intracranially injected with USP21-depleted and FOXD1-overexpressed GSCs. We observed that inhibition of tumor growth was alleviated (Fig. 4f). Accordingly, the results of H&E staining indicated that the xenografts carrying USP21 shRNA GSCs displayed restricted tumor growth, whereas this effect is reversed by overexpression of FOXD1 (Fig. 4h). Furthermore, Kaplan-Meier survival curve showed that the mice injected with USP21-depleted GSCs lived much longer than those injected with control shRNA GSCs, and the survival time in mice intracranially injected with USP21-depleted GSCs significantly decreased after FOXD1 overexpression (Fig. 4g). Collectively, these results indicate that USP21 facilitates MES GBM tumorigenicity via FOXD1 to some extent.

### Pharmacological inhibition of USP21 by disulfiram promotes FOXD1 ubiquitination and hinders tumor growth

**Fig. 5:**
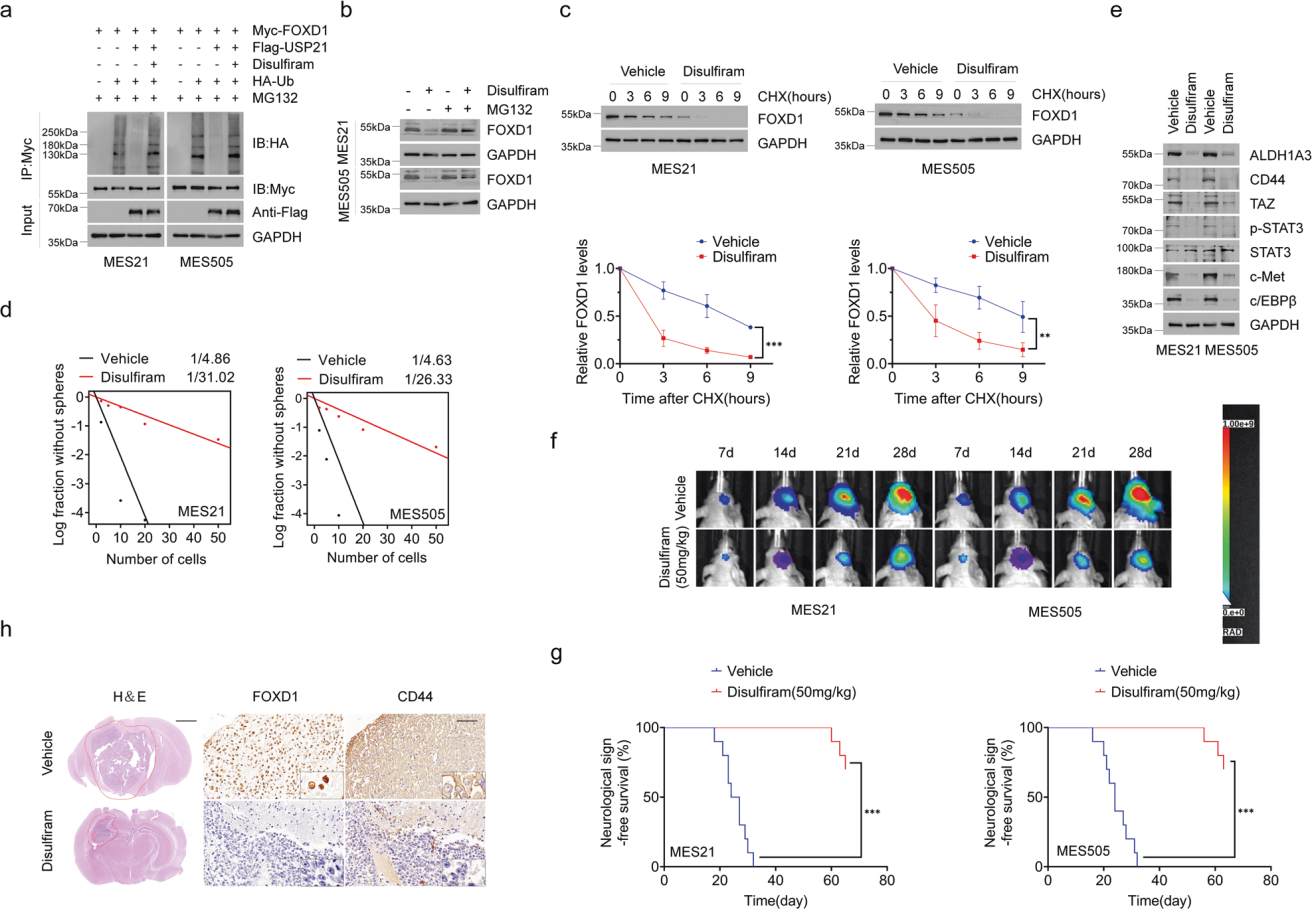
Pharmacological inhibition of USP21 by disulfiram promotes FOXD1 ubiquitination and retards tumor growth. **a** Co-IP showing that the ability of USP21 to remove ubiquitin moieties from polyubiquitinated FOXD1 was almost completely abrogated by disulfiram. **b** Western blotting showing that disulfiram, like USP21 knockdown, promotes FOXD1 ubiquitination and degradation. **c** Western blotting showing that disulfiram induced a marked decrease in the stabilization of FOXD1 protein. ***P < 0.001, **P < 0.01. **d** In vitro limiting dilution sphere-forming frequency showing that the disulfiram treatment reduced the tumorsphere formation frequency of MES21 and 505 GSCs. **P < 0.01. **e** Western blotting showing that disulfiram treatment notably inhibited the core MES GSC markers including ALDH1A3, CD44, TAZ, p-STAT3, c-MET and c/EBPβ. **f** Representative bioluminescent images showing that tumor-bearing mice receiving disulfiram treatment showed retarded tumor growth compared with vehicle-treated mice. *n* = 10. **g** Kaplan–Meier survival curves showing that mice bearing xenograft tumors receiving disulfiram treatment had a shorter lifespan than vehicle-treated mice. *n* = 10, ***P < 0.001, Log-rank test. **h** Representative H&E and IHC images showing that disulfiram attenuated the tumor growth and the expression of FOXD1 and CD44 in tumor tissues.

A recent study has shown that disulfiram, a clinically used anti-alcoholism drug, could function as a potent inhibitor of USP21 [31]. In order to corroborate our USP21-knockdown findings, we assessed whether disulfiram also abrogates USP21-mediated FOXD1 stabilization.We first determined whether disulfiram could block the deubiquitinating activities of USP21. As shown in Fig. 5a, the ability of USP21 to remove ubiquitin moieties from FOXD1 was markedly attenuated by disulfiram. Accordingly, the protein, but not mRNA, levels of FOXD1 were found to be reduced in MES 21 and 505 GSCs after disulfiram treatment, which were partially restored by treating with MG132 (Fig. 5b and Supplementary Fig. 4a). Moreover, co-treatment with disulfiram and CHX induced a marked decrease in the half-life of FOXD1 protein (Fig. 5c). These data indicate that disulfiram, similar with USP21 depletion, facilitates FOXD1 ubiquitination and its subsequent proteasomal degradation.

We then examined the in vitro and in vivo anti-tumor effects of disulfiram on patient-derived MES GSCs. Disulfiram treatment markedly inhibited tumorsphere-forming ability of MES 21 and 505 GSCs (Fig. 5d), accompanied by loss of MES GSC markers ALDH1A3, CD44, TAZ, p-STAT3, c-MET and c-EBPβ (Fig. 5e). Furthermore, we treated mice harboring intracranial tumors derived from luciferase-expressing MES 21 and 505 GSCs with 50 mg/kg disulfiram. Compared with vehicle-treated mice, tumor-bearing mice receiving disulfiram treatment showed reduced tumor incidence and extended survival (Fig. 5f, g). Interestingly, we found that the combination of disulfiram and TMZ, a first-line chemotherapy drug for GBM, had a significant synergistic effect on tumor-bearing mice (Supplementary Fig. 5a, b). Consistent with these findings, IHC analysis showed that disulfiram attenuated the expression of FOXD1 and CD44 in tumor tissues obtained from orthotopic GBM xenografts (Fig. 5h). Together, these results suggest that disulfiram-induced FOXD1 ubiquitination contributes to the inhibition of tumor growth of patient-derived MES GSCs.

### USP21 positively correlates with FOXD1 protein levels and is associated with the poor prognosis of GBM patients

**Fig. 6:**
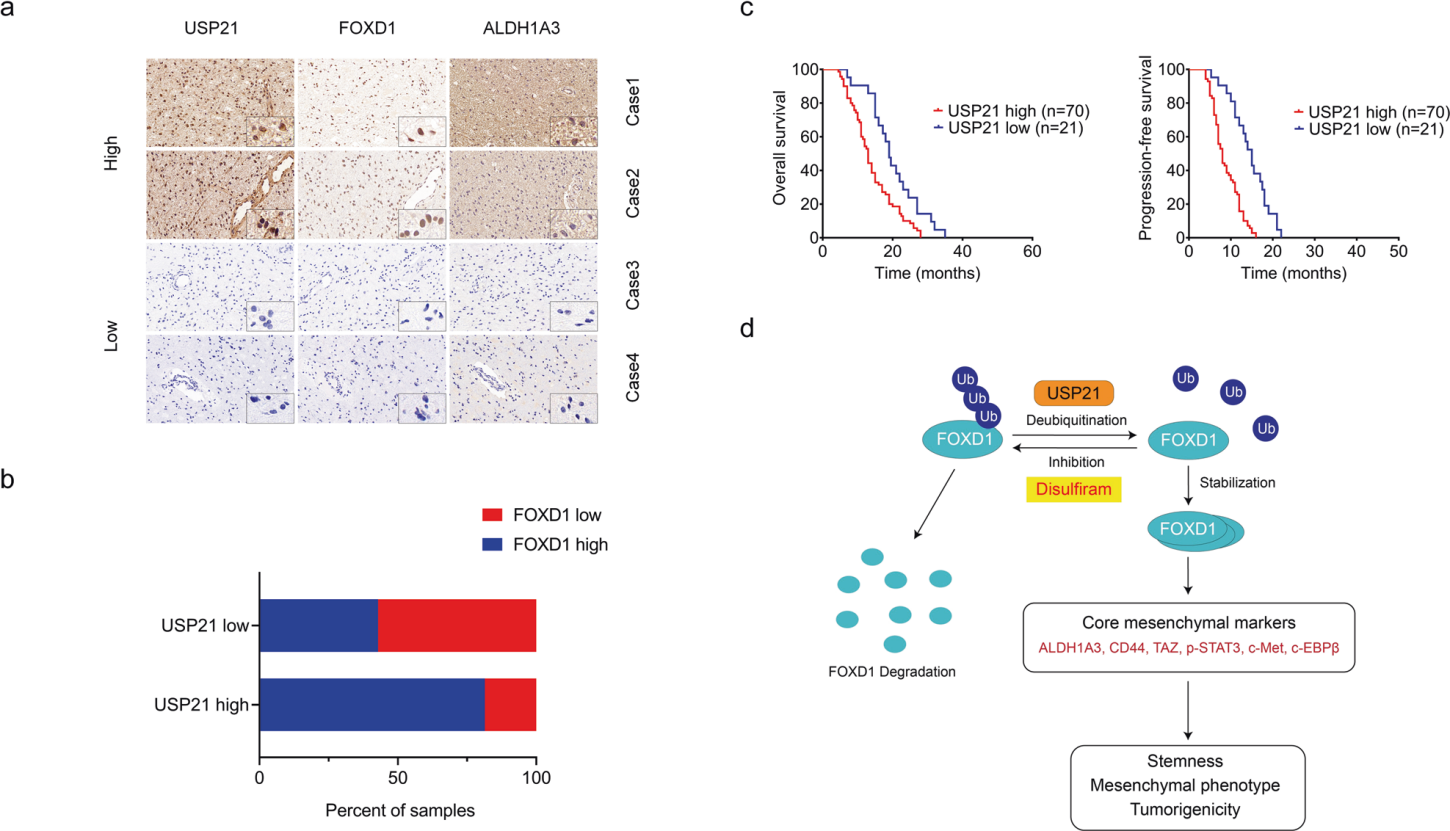
USP21 positively correlates with FOXD1 protein levels and is associated with the poor prognosis of MES GBM patients. **a** Representative IHC images of USP21 and FOXD1 expression in MES GBM specimens. **b** Correlation between USP21 and FOXD1 proteins in 91 MES GBM IHC results. **c** Kaplan–Meier plots of overall survival and progression-free survival of 91 MES GBM patients stratified by protein expression of USP21 showing that USP21^hi^ MES GBM patients displayed significantly shorter overall survival and progression-free survival than USP21^low^ MES GBM patients. **P < 0.01. **d** Schematic illustration of USP21-mediated FOXD1 stabilization, promoting self-renewal and tumorigenicity of MES GSCs.

To determine the possible clinical relevance between USP21 and FOXD1, we examined the expression levels of USP21 and FOXD1 in four clinical MES GBM tissues. In case 1 and 2, a high expression of USP21 correlated with a high expression of FOXD1. Conversely, in case 3 and 4, a low expression of USP21 correlated with a low expression of FOXD1 (Fig. 6a). In addition, the other 91 IHC results supported the positive correlation between USP21 and FOXD1 (Fig. 6b). Moreover, Kaplan–Meier survival curve analysis of the 91 GBM tissues showed that the survival rate of patients with high USP21 expression was significantly lower than that of patients with low USP21 expression (Fig. 6c). In addition, similar results were obtained from the CGGA database (Supplementary Fig. 6a, b). Overall, our results suggest that there is a strong positive clinical relevance between the levels of USP21 and FOXD1 and that high USP21 expression in patients indicates a poor prognosis.

## Discussion

Despite multimodal therapy strategies, GBM remains the most aggressive and challenging brain tumors. GSCs are at the apex of the GBM cellular hierarchy, which inevitably mediate disease recurrence and therapeutic resistance [32]. Therefore, eliminating GSCs is critical for improving GBM treatment [33, 34]. As a key member of the FOX family of transcription factors, FOXD1 has been recently found to be crucial for maintaining the MES properties of GSCs by modulating the downstream target ALDH1A3 [13]. However, the molecular basis for sustained protein levels of FOXD1 in GSCs remains unclear. In this study, by adopting the unbiased DUB-focused siRNA screen, we identified USP21 as a novel DUB that governs FOXD1 stability. We found that USP21 interacted with FOXD1 and stabilized its protein levels by reducing its K48-linked polyubiquitination, thereby sustaining high FOXD1 expression and activating its downstream signaling (Fig. 6d). Recent studies have shown that USP21 is frequently dysregulated in multiple types of cancer and plays a critical role in cancer development and progression. Indeed, our clinical data showed a strong positive correlation between the expression levels of USP21 and FOXD1 in GBM patients and demonstrated that elevated USP21 expression was negatively associated with GBM patient survival. The functional results reveal that genetic inhibition of USP21 resulted in a dramatic decrease in FOXD1 expression, with concomitant downregulation of ALDH1A3, CD44 and core MES-associated transcription factors (C/EBPβ, TAZ, and p-STAT3). Accordingly, depletion of USP21 markedly reduced GSC tumorigenicity and prolonged the survival of tumor-bearing mice. All these effects can be reversed by ectopic expression of FOXD1, thus strongly supporting our notion that USP21-mediated FOXD1deubiquitination sustains MES properties of GSCs, and eventually contributes to tumor progression in GBM.

Due to the key role of DUBs in a variety of human cancers, pharmacological inhibition of DUBs has emerged as a promising therapy for cancer treatment. In this regard, an increasing number of DUB inhibitors have been identified, and are currently being tested in preclinical studies and clinical trials [35, 36]. In this study, we identified disulfiram could serve as a USP21-specific inhibitor, which induce robust polyubiquitylation of FOXD1 and elicit substantial antitumor activity in preclinical models of GBMs. Disulfiram, a drug used to treat alcoholism, has emerged as anattractive candidate for drug repurposing in cancer treatment [37]. Thus far, several molecular mechanisms have been proposed for its anti-cancer effects [38]. For instance, disulfiram has been demonstrated to trigger proteasome inhibition that leads to the accumulation of misfolded proteins and possible toxic protein aggregates [39–41]. Moreover, a recent study has identified valosin-containing protein (VCP)/p97 segregase adaptor NLP4 as new molecular target of disulfiram [42, 43]. Furthermore, disulfiram has been shown to induce the oncoprotein MLL degradation, which efficiently kills pediatric glioma cell lines as well as patient-derived GSCs [44]. Based on the aforementioned targets and our findings, we conclude that the anti-tumor effects of disulfiram is multi-modal, which might be more effective than single-target agents.

In summary, our study identifies USP21 as a *bonafide* DUB for FOXD1, which stabilizes FOXD1 to maintain the MES properties of GSCs. Employing both genetic and pharmacological approaches, we provide evidence that targeting FOXD1 stabilization through USP21 inhibition may thus open an avenue for therapeutic intervention in GBMs, particularly in patients with therapy-resistant MES subtypes.

## Supplementary information

Supplemental Figure 1

Supplemental Figure 2

Supplemental Figure 3

Supplemental Figure 4

Supplemental Figure 5

Supplemental Figure 6

Original Data File

Reproducibility checklist
