# Supplementary figures and images for: USP21 promotes self-renewal and tumorigenicity of mesenchymal glioblastoma stem cells by deubiquitinating and stabilizing FOXD1
Source: Cell Death Dis. 2022 Aug 16;13(8):712. doi: 10.1038/s41419-022-05163-3 (PMC9381540; doi:10.1038/s41419-022-05163-3)

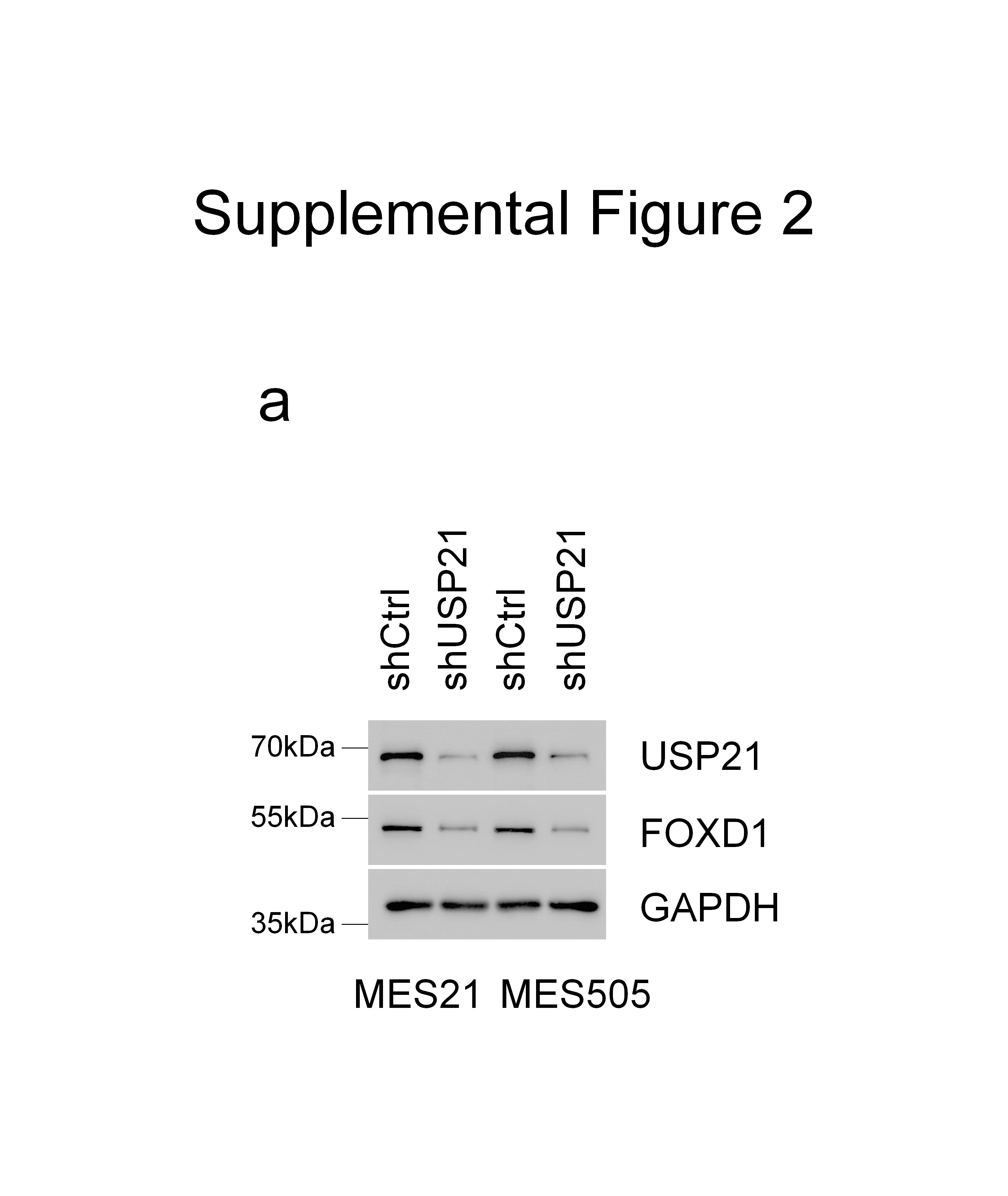

Supplement: Supplementary file 2 — Supplemental Figure 2 [file 41419_2022_5163_MOESM2_ESM.jpg]

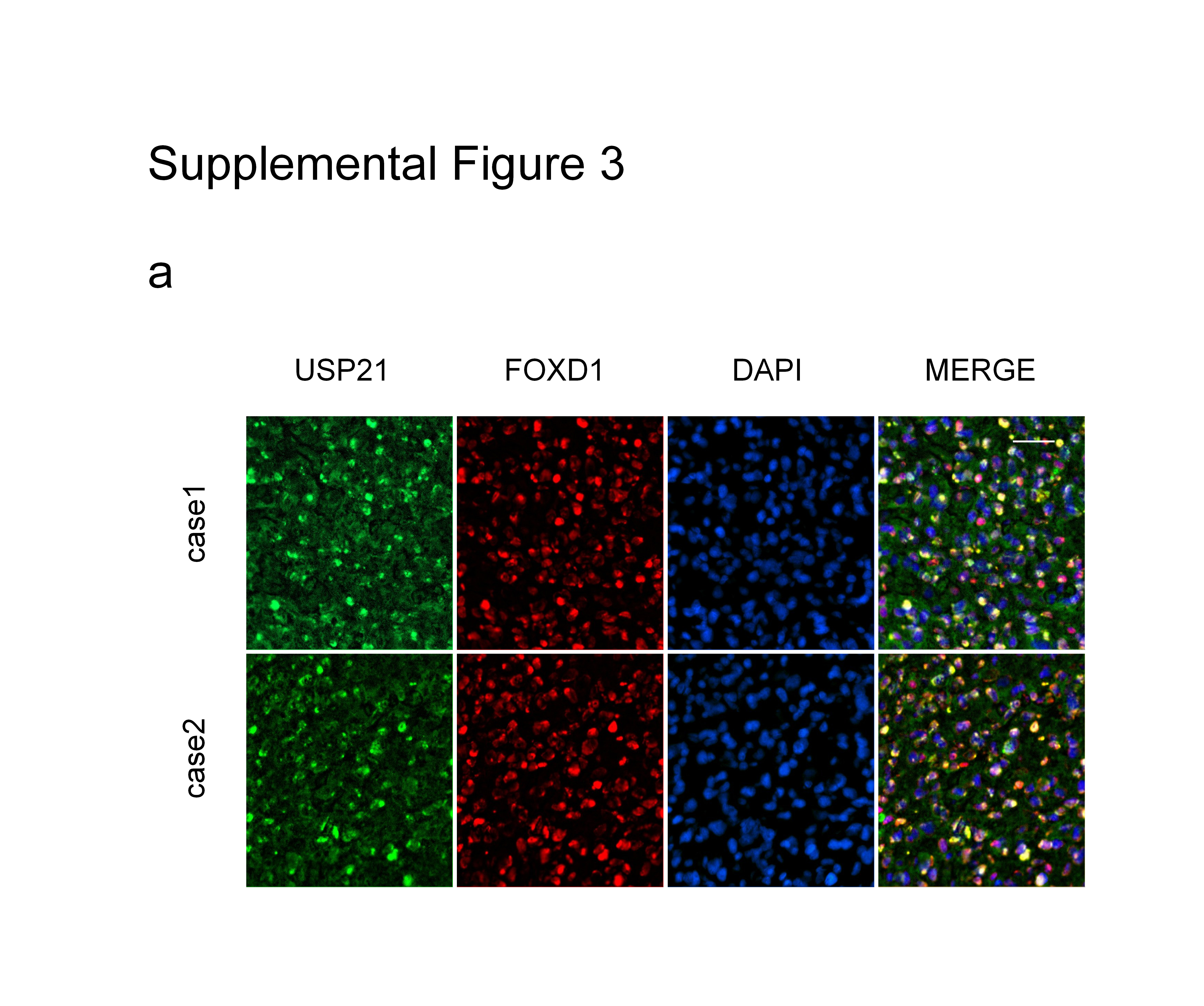

Supplement: Supplementary file 3 — Supplemental Figure 3 [file 41419_2022_5163_MOESM3_ESM.jpg]

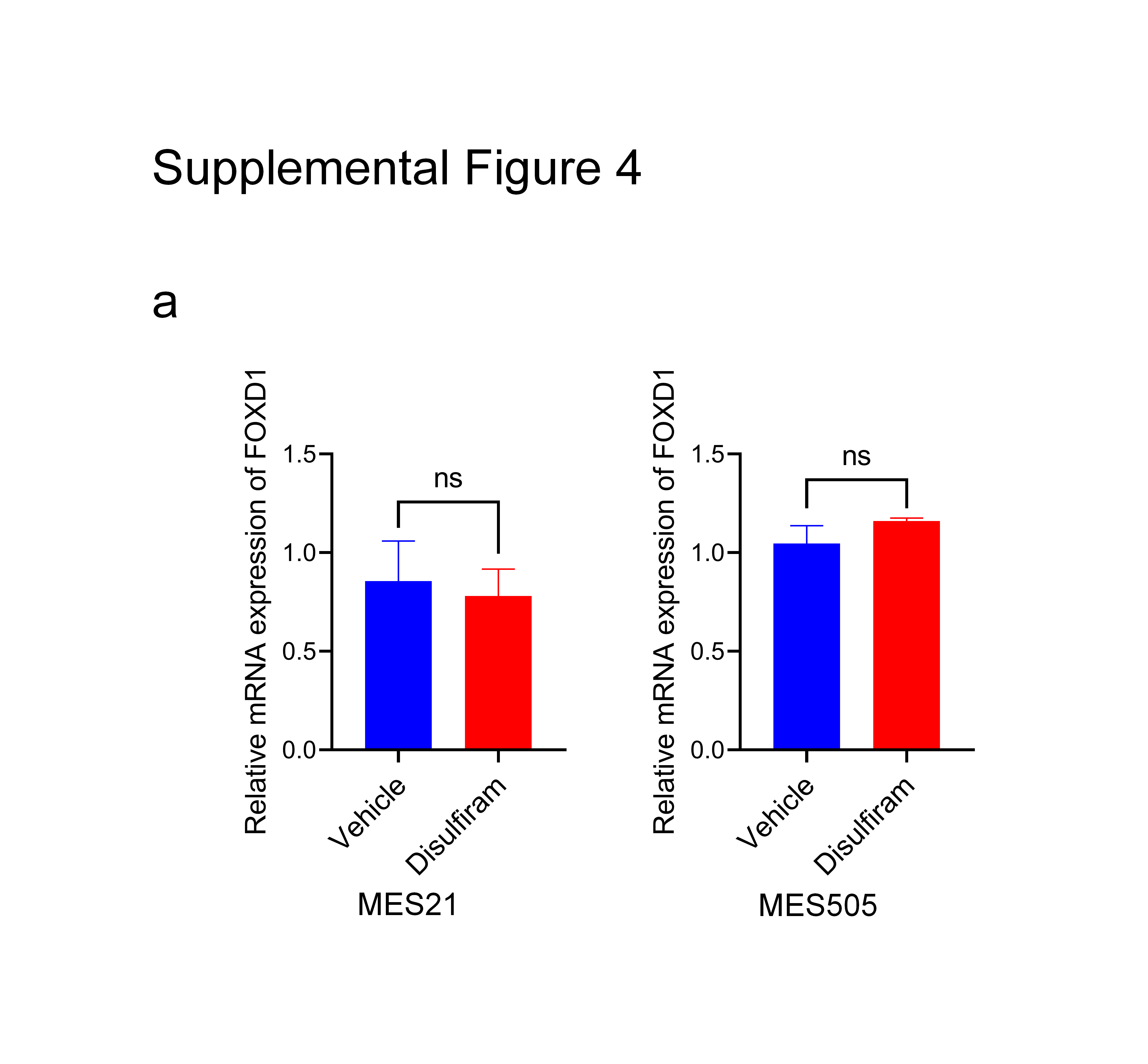

Supplement: Supplementary file 4 — Supplemental Figure 4 [file 41419_2022_5163_MOESM4_ESM.jpg]

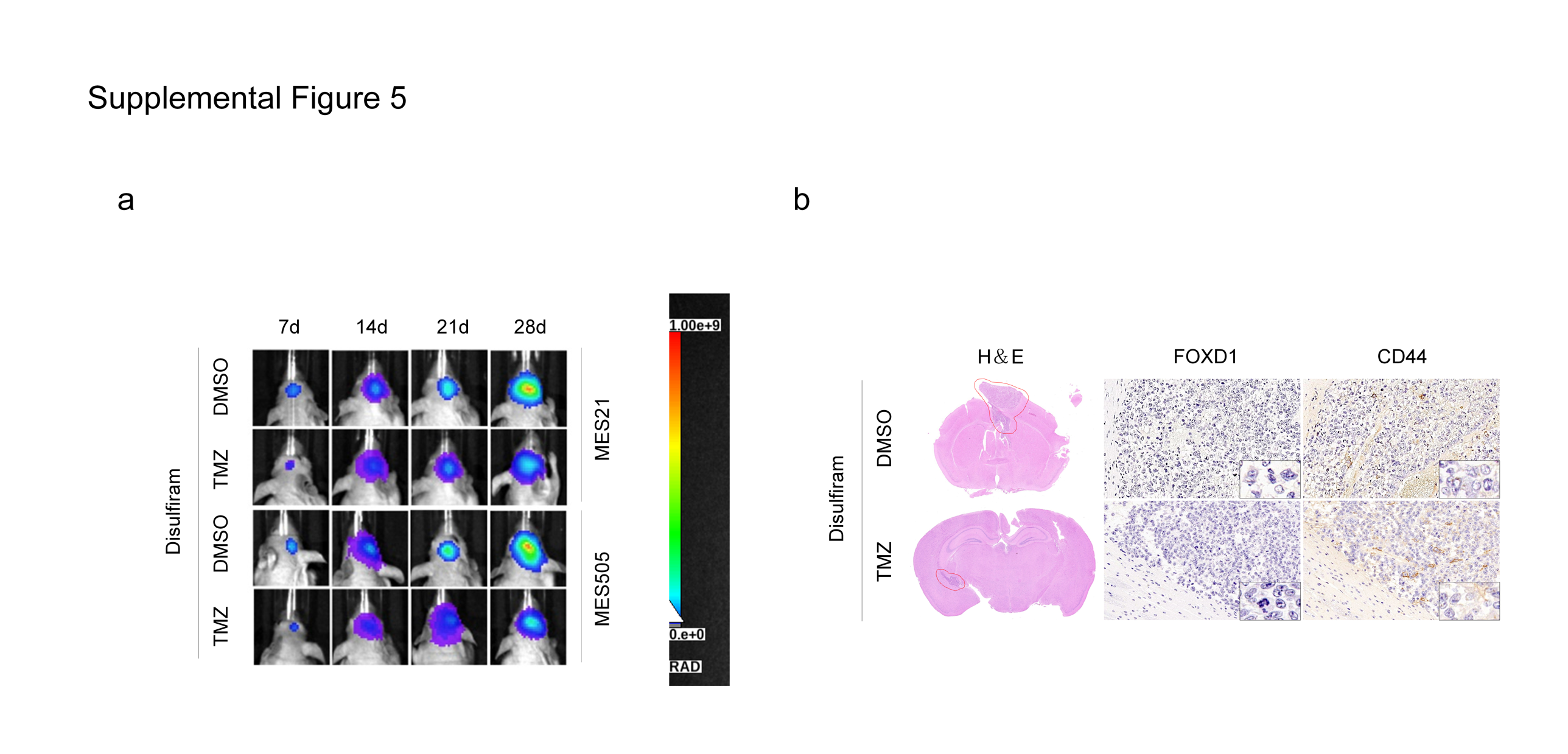

Supplement: Supplementary file 5 — Supplemental Figure 5 [file 41419_2022_5163_MOESM5_ESM.jpg]
