# Supplementary material for: USP21 promotes self-renewal and tumorigenicity of mesenchymal glioblastoma stem cells by deubiquitinating and stabilizing FOXD1
Source: Cell Death Dis. 2022 Aug 16;13(8):712. doi: 10.1038/s41419-022-05163-3 (PMC9381540; doi:10.1038/s41419-022-05163-3)

Figure 1a

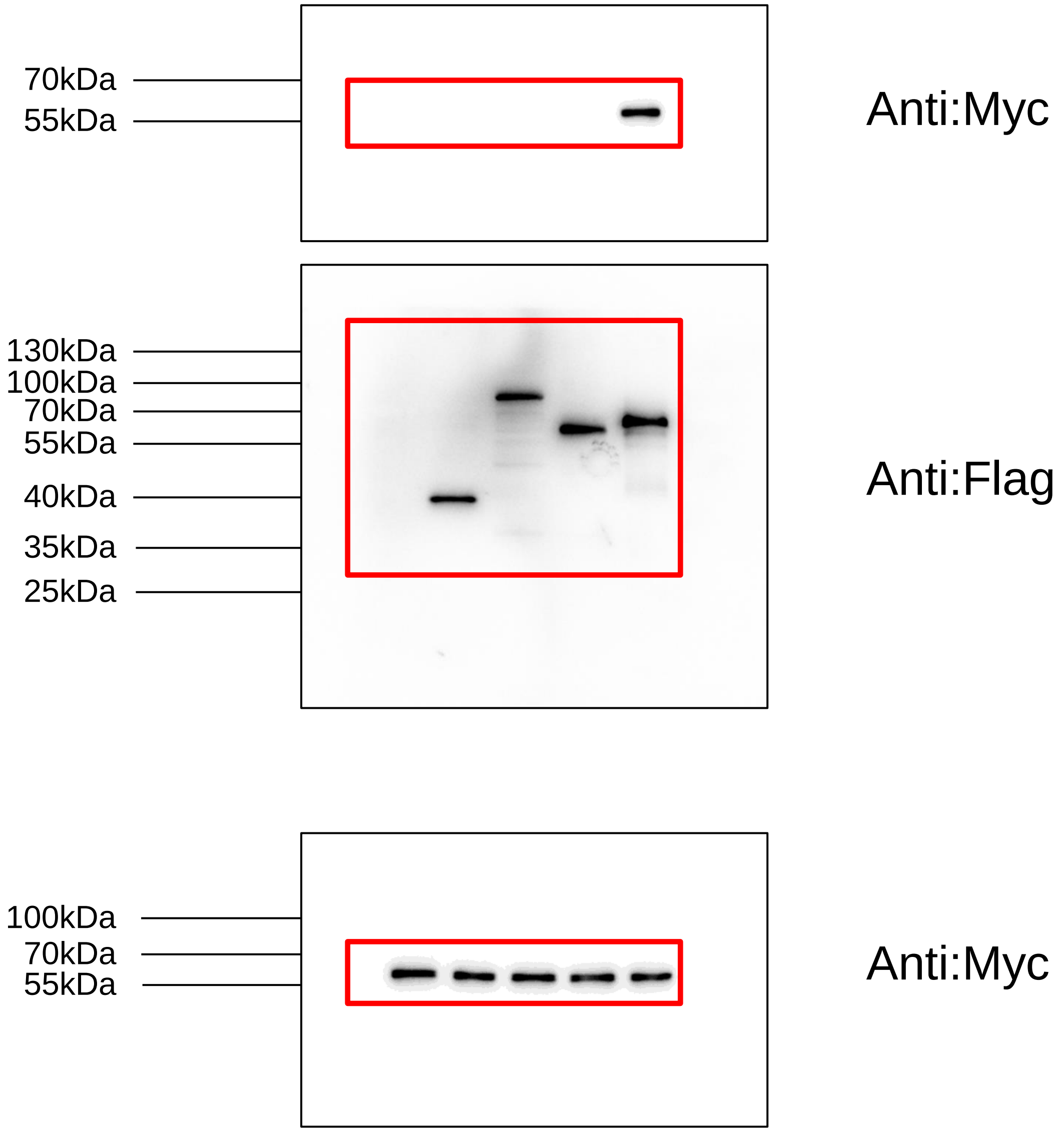
70kDa
55kDa
Anti:Myc
130kDa
100kDa
70kDa
55kDa
40kDa
35kDa
25kDa
Anti:Flag
100kDa
70kDa
55kDa
Anti:Myc

Figure 1b

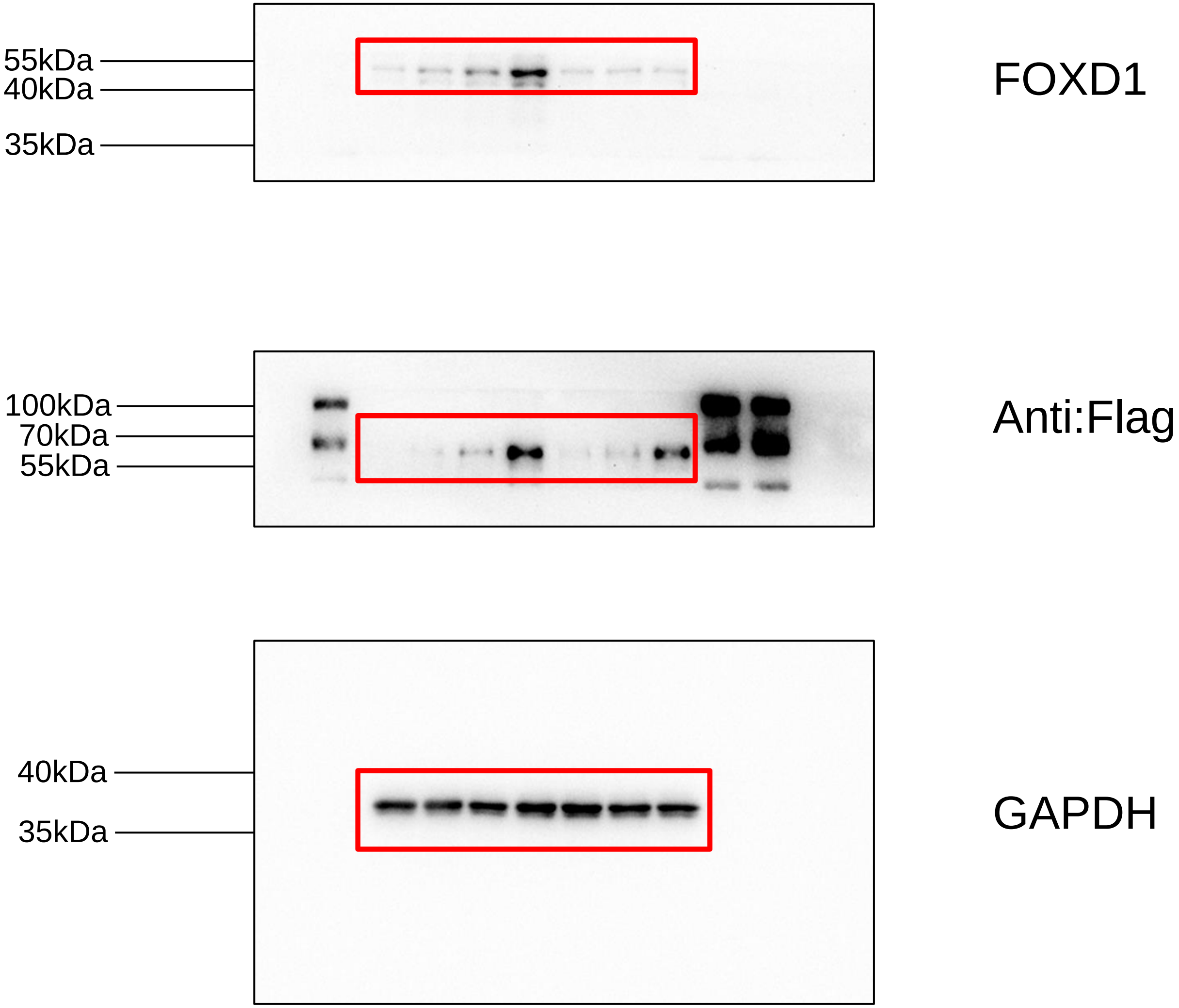
55kDa
40kDa
35kDa
FOXD1
100kDa
70kDa
55kDa
Anti:Flag
40kDa
35kDa
GAPDH

Figure 1c

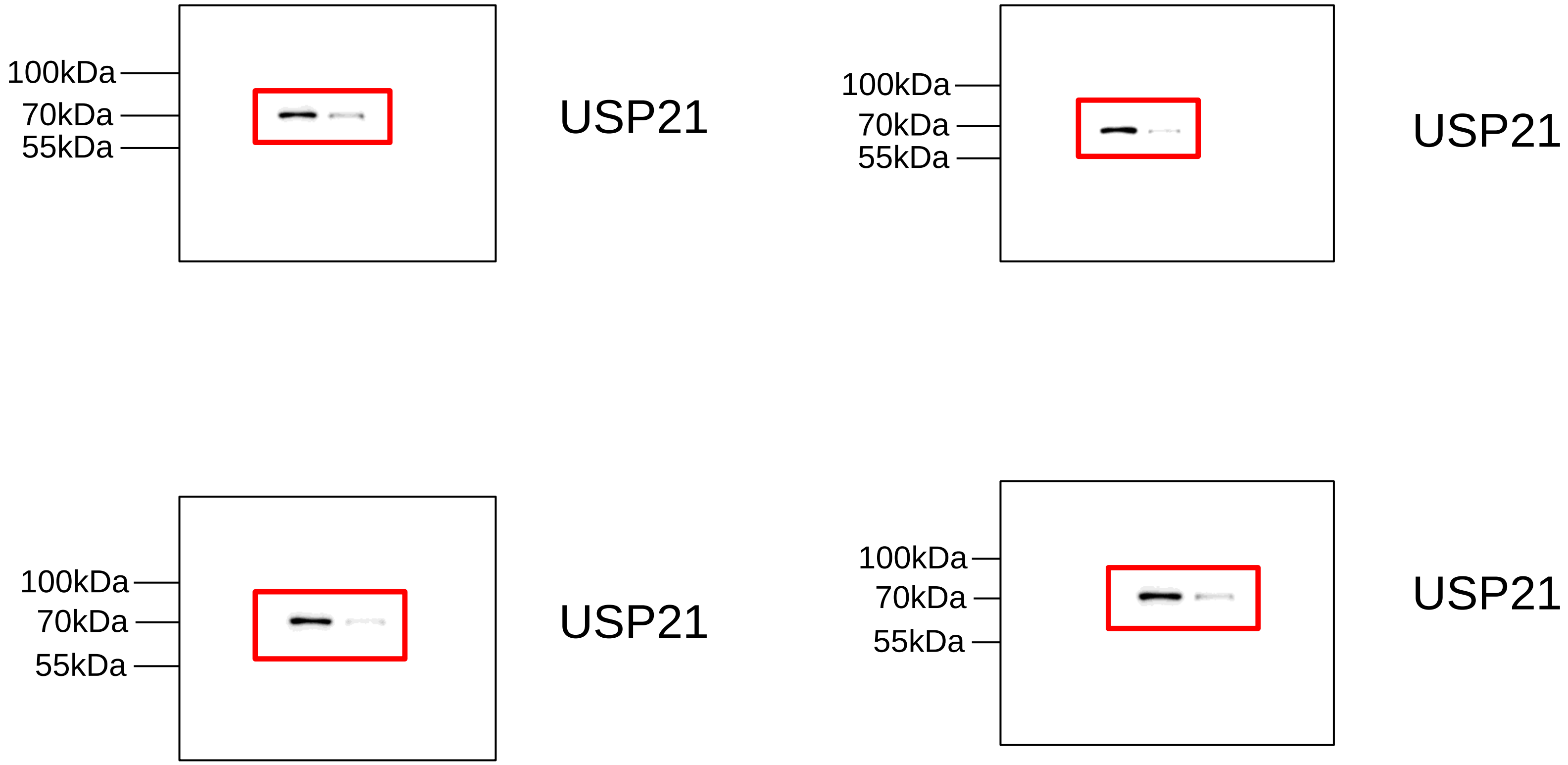
100kDa
70kDa
55kDa
USP21
100kDa
70kDa
55kDa
USP21
100kDa
70kDa
55kDa
USP21
100kDa
70kDa
55kDa
USP21

Figure 1c

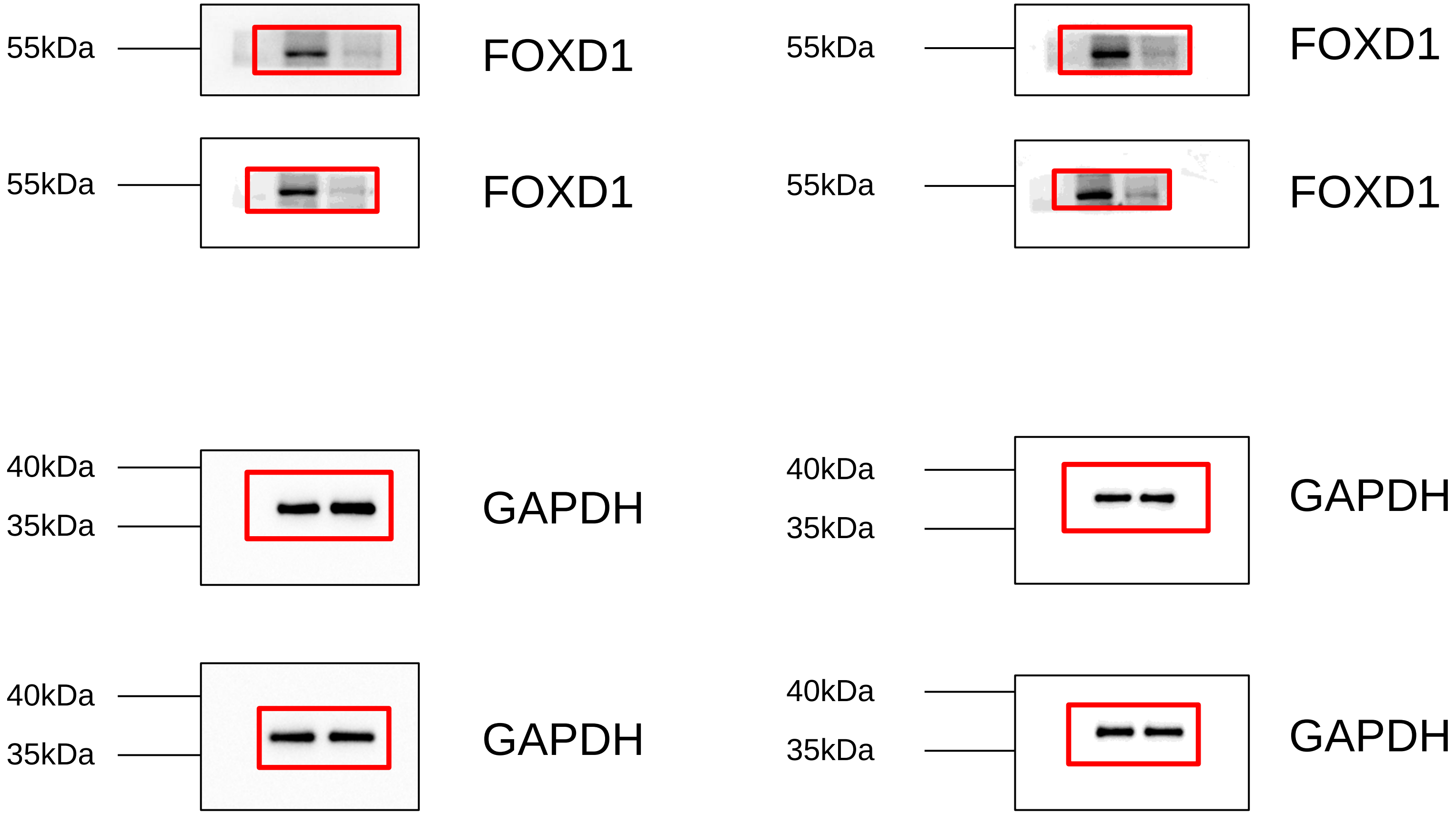
55kDa
FOXD1
55kDa
FOXD1
55kDa
FOXD1
55kDa
FOXD1
40kDa
35kDa
GAPDH
40kDa
35kDa
GAPDH
40kDa
35kDa
GAPDH
40kDa
35kDa
GAPDH

Figure 1d

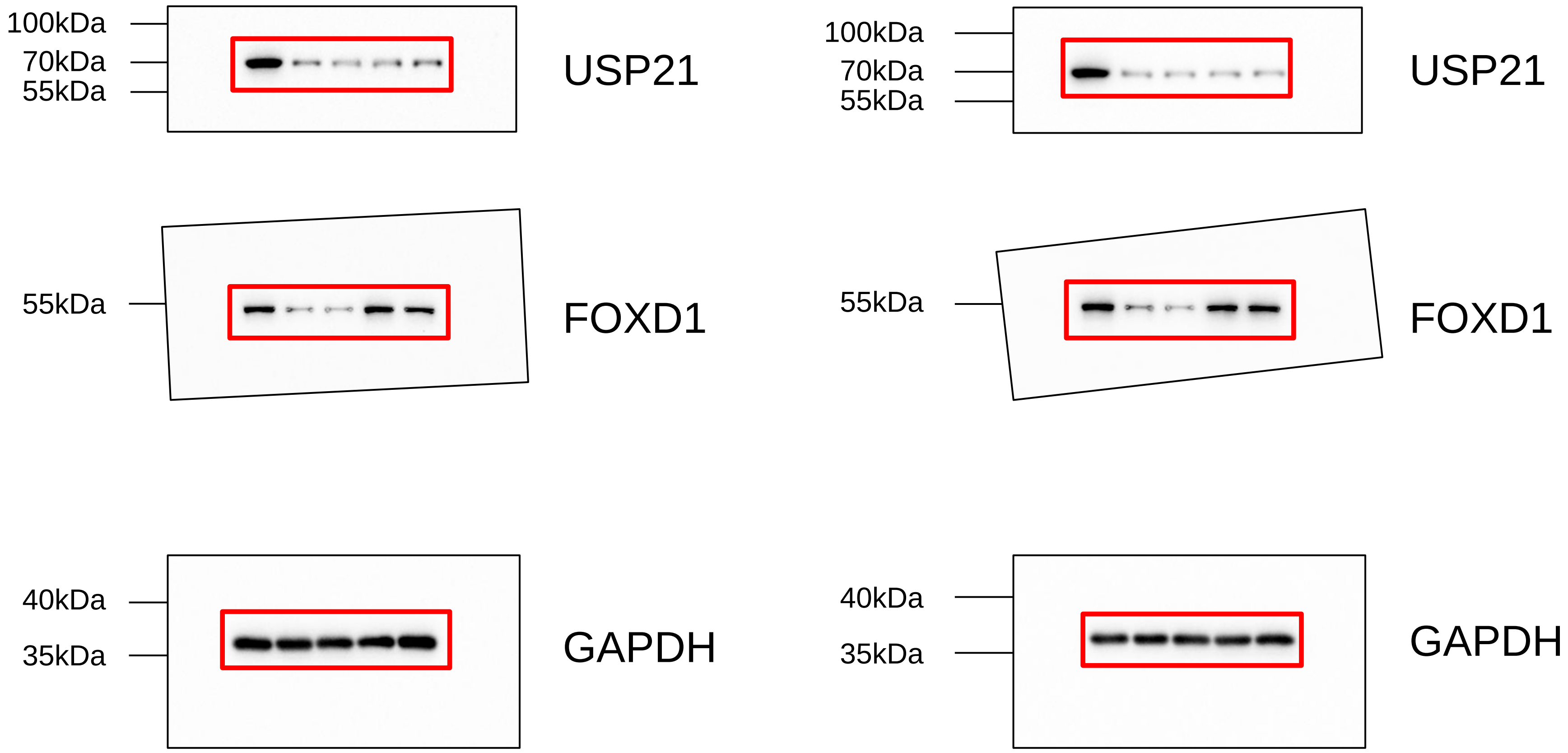
100kDa
70kDa
55kDa
USP21
100kDa
70kDa
55kDa
USP21
55kDa
FOXD1
55kDa
FOXD1
40kDa
35kDa
GAPDH
40kDa
35kDa
GAPDH

Figure 1e

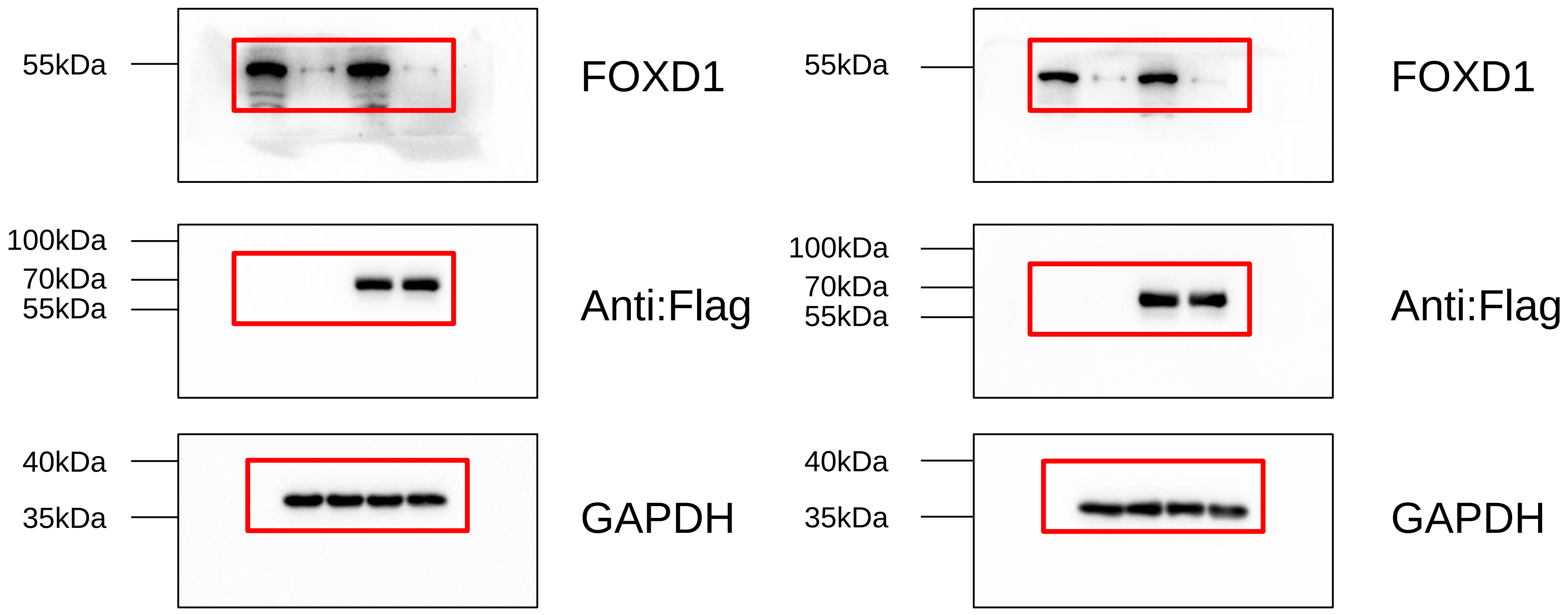
55kDa
FOXD1
100kDa
70kDa
55kDa
Anti:Flag
40kDa
35kDa
GAPDH
55kDa
FOXD1
100kDa
70kDa
55kDa
Anti:Flag
40kDa
35kDa
GAPDH

Figure 1f

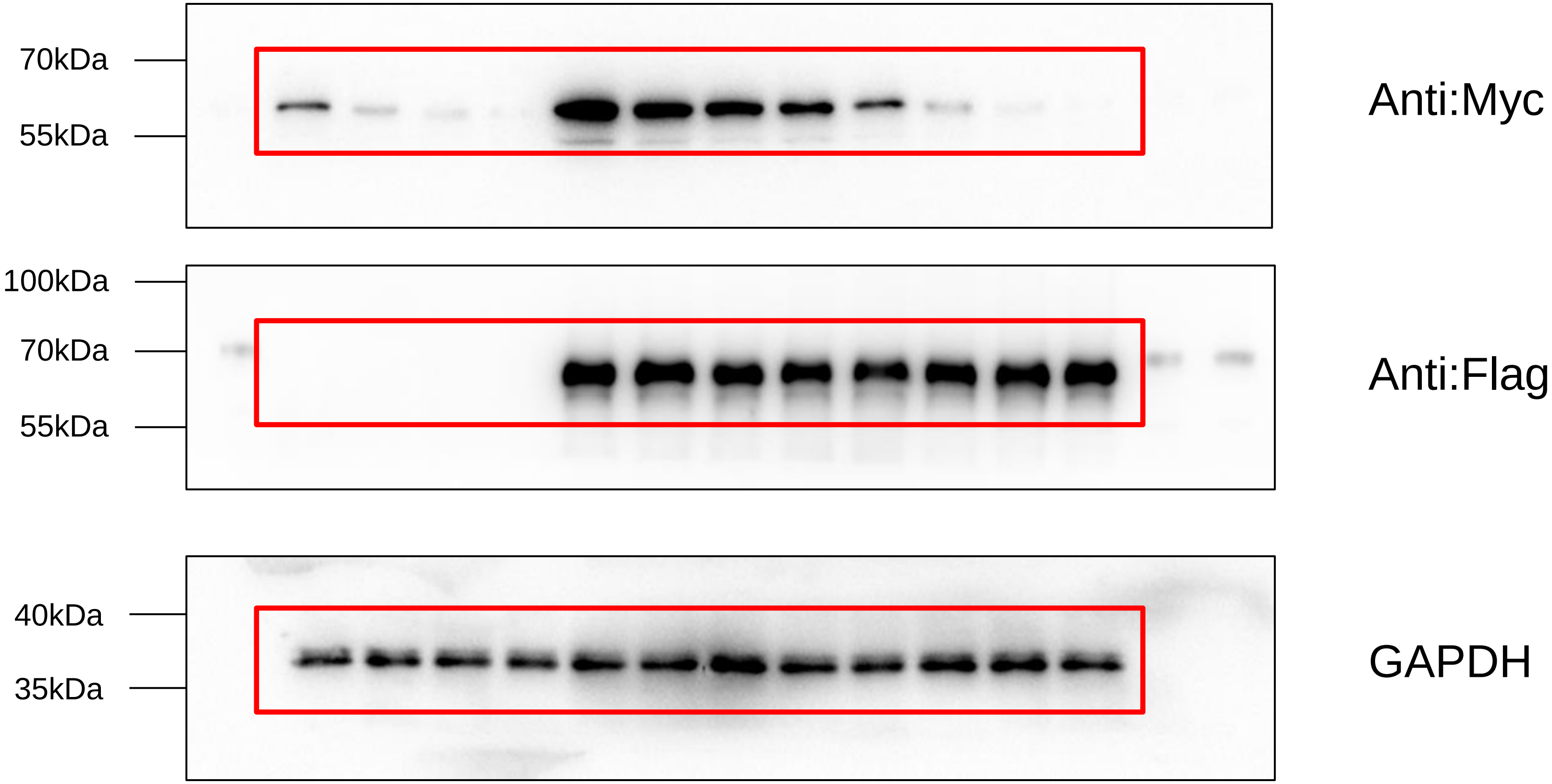

70kDa
55kDa
Anti:Myc
100kDa
70kDa
55kDa
Anti:Flag
40kDa
35kDa
GAPDH

Figure 1g

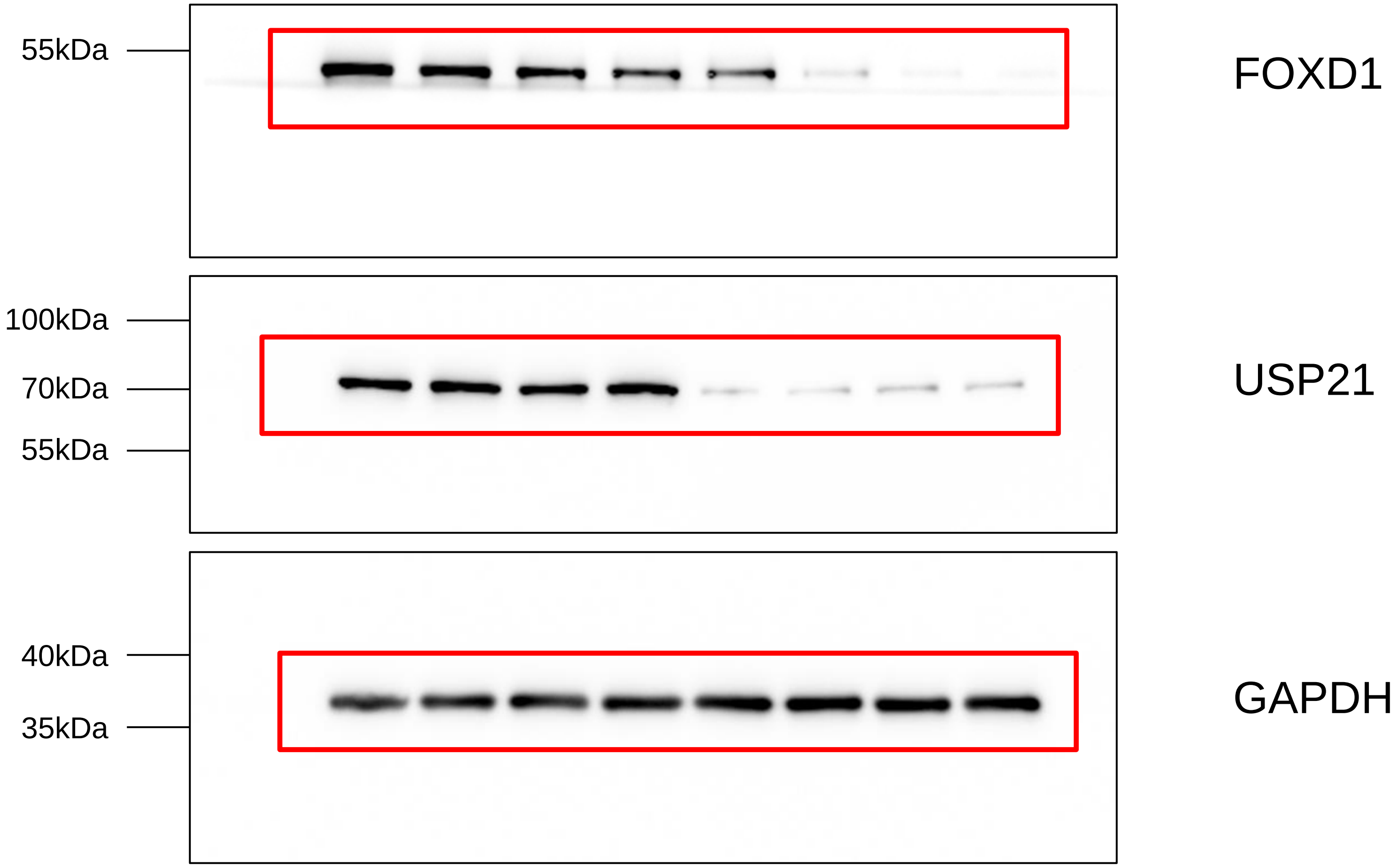

Figure 1h

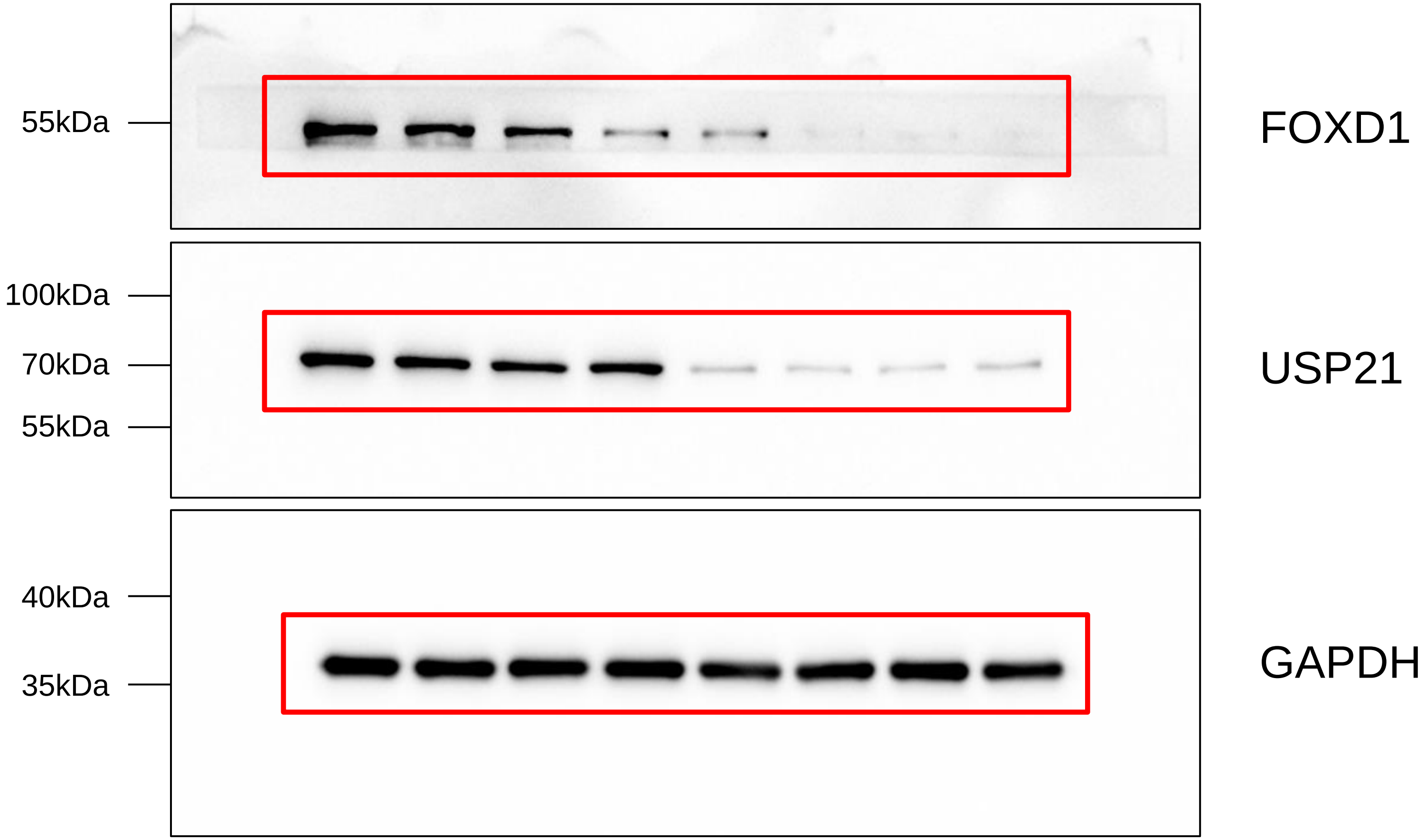

Figure 2a

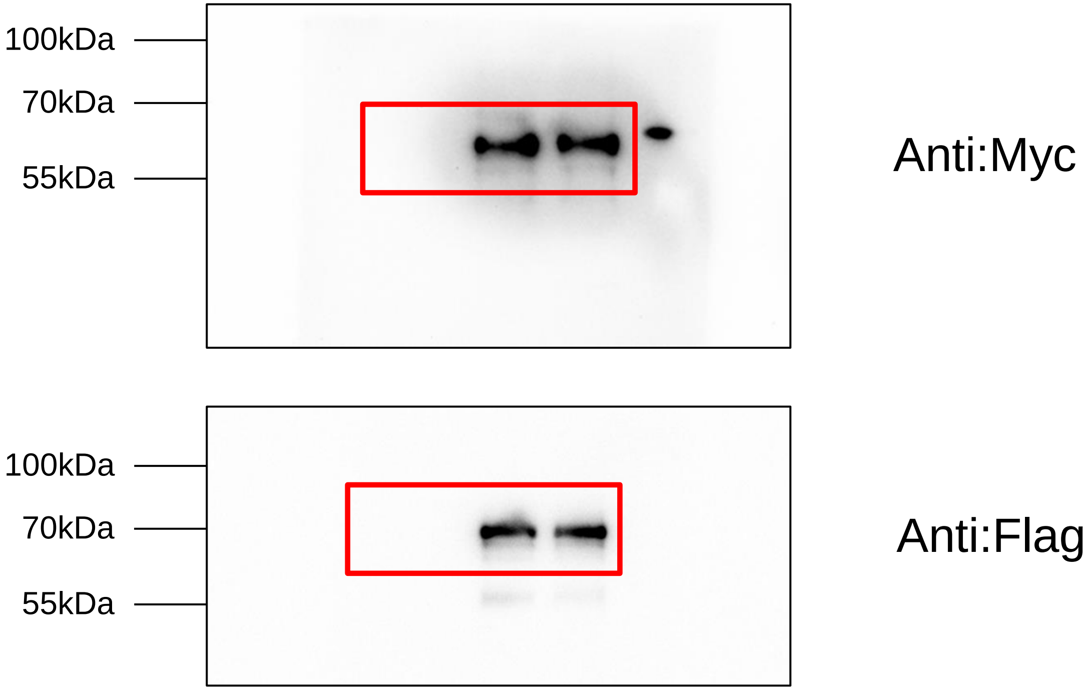

Figure 2a

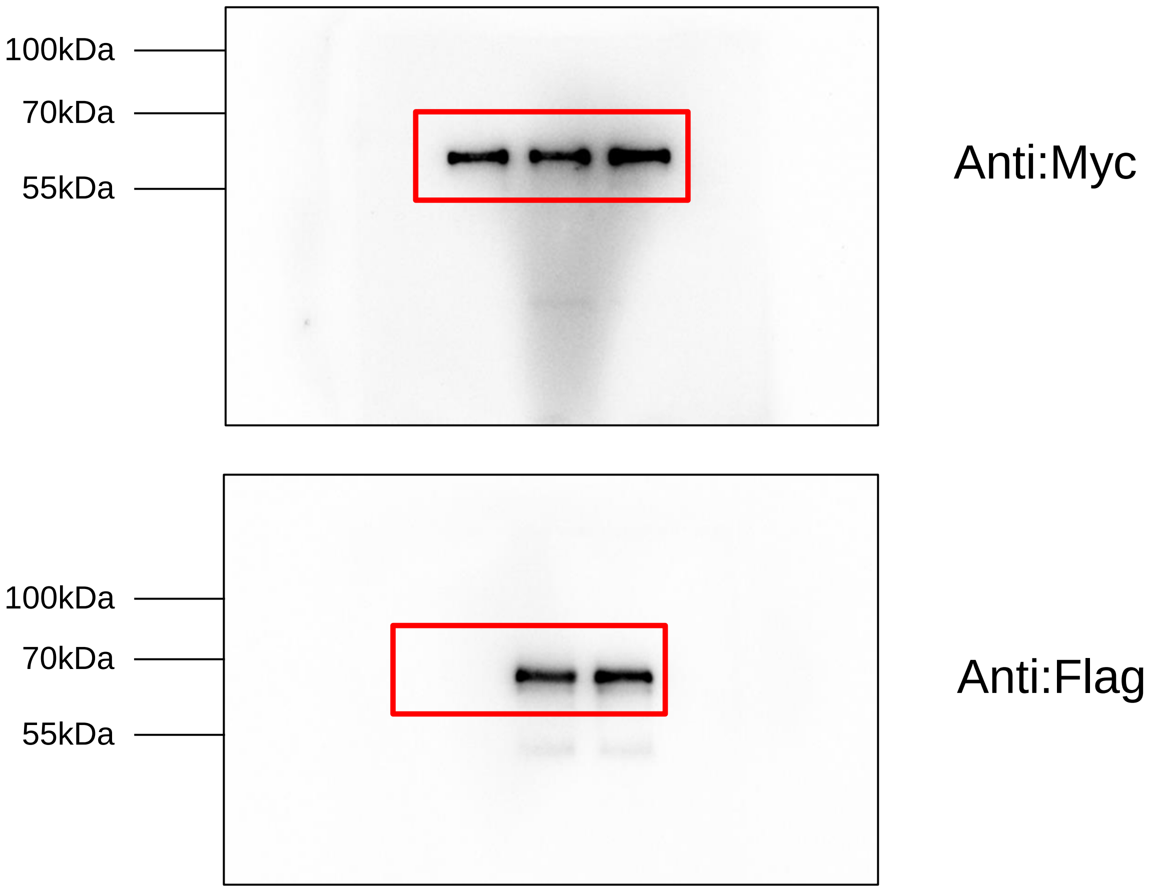

Figure 2b

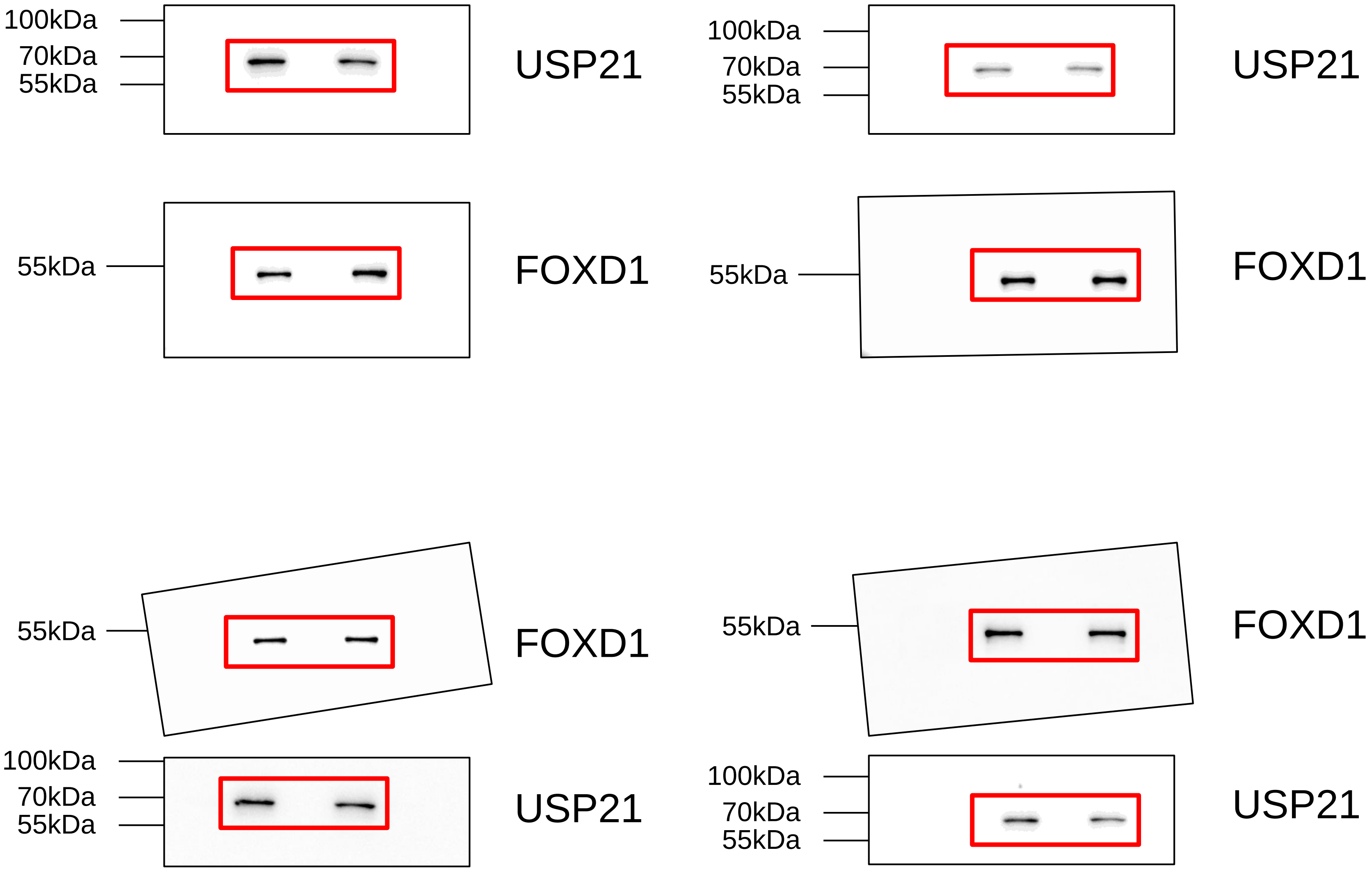
100kDa
70kDa
55kDa
USP21
100kDa
70kDa
55kDa
USP21
55kDa
FOXD1
55kDa
FOXD1
55kDa
FOXD1
55kDa
FOXD1
100kDa
70kDa
55kDa
USP21
100kDa
70kDa
55kDa
USP21

Figure 2c

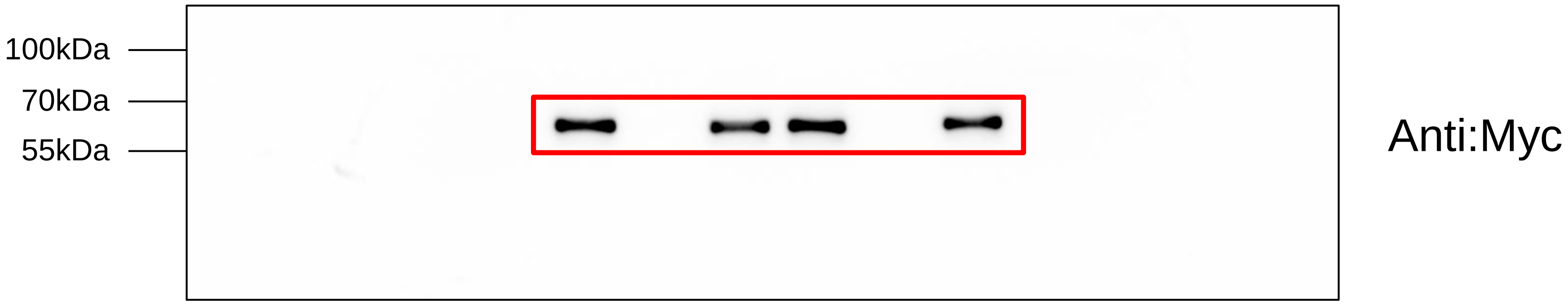

Figure 2e

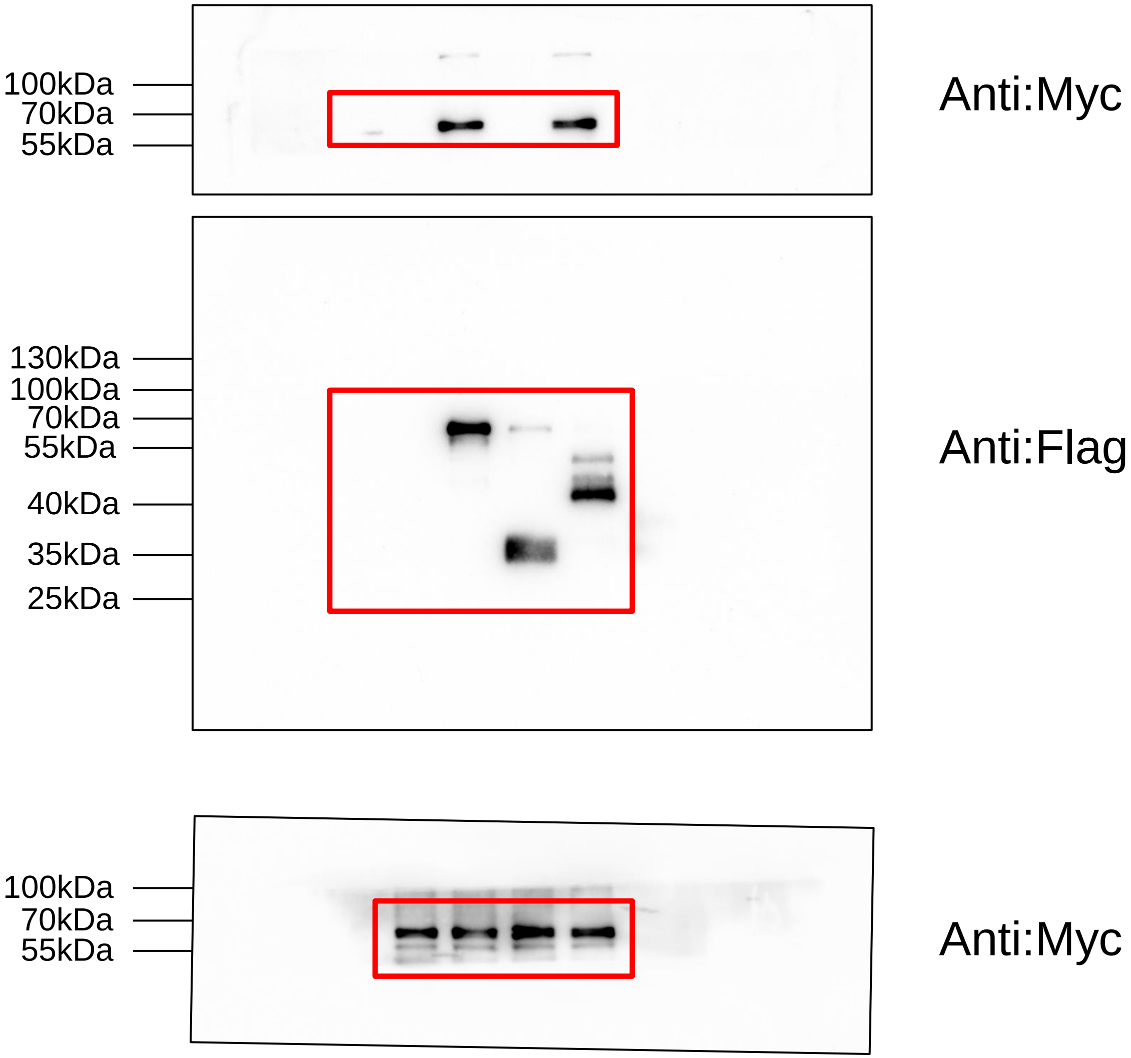
100kDa
70kDa
55kDa
Anti:Myc
130kDa
100kDa
70kDa
55kDa
40kDa
35kDa
25kDa
Anti:Flag
100kDa
70kDa
55kDa
Anti:Myc

Figure 2e

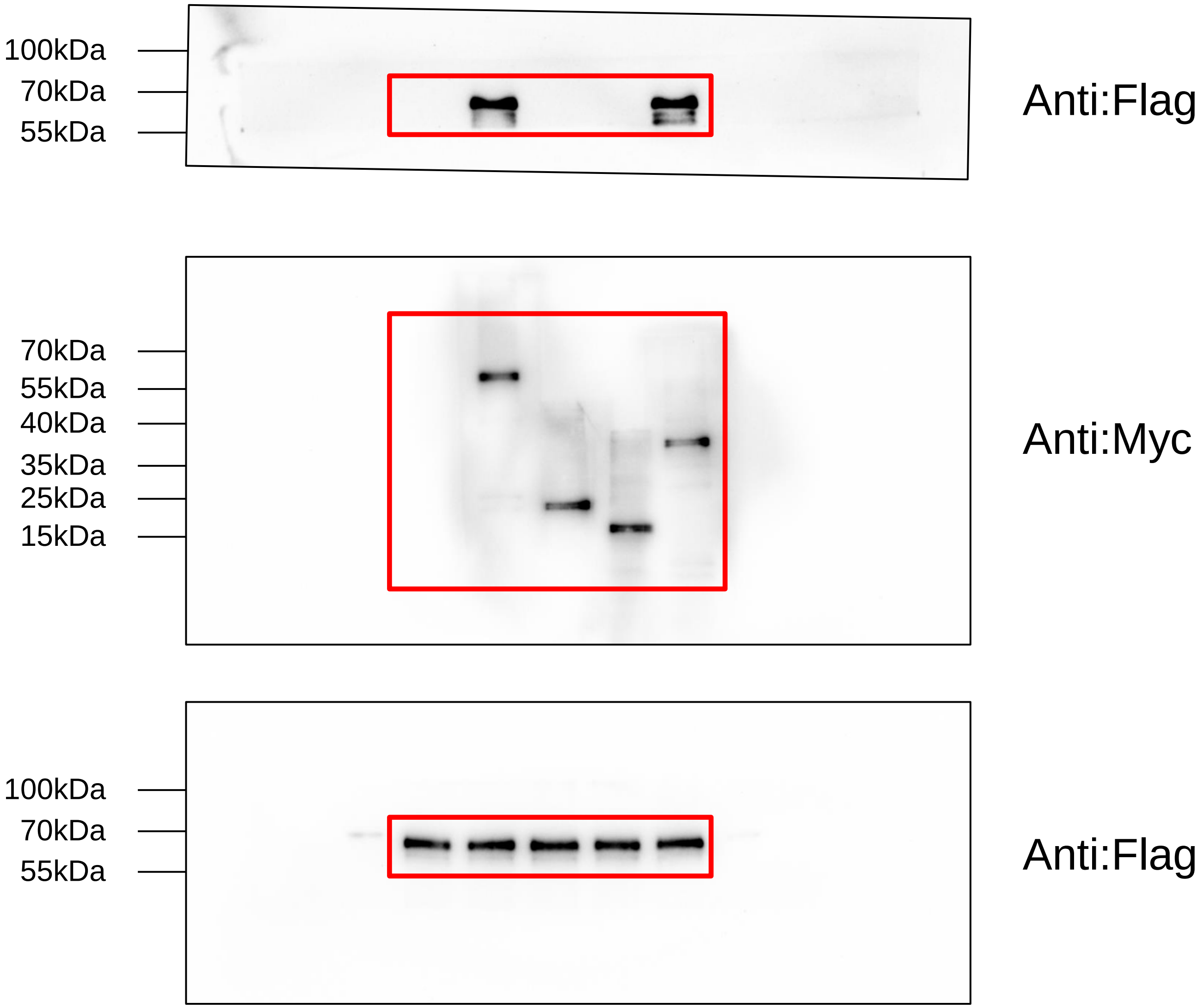
100kDa
70kDa
55kDa
Anti:Flag
70kDa
55kDa
40kDa
35kDa
25kDa
15kDa
Anti:Myc
100kDa
70kDa
55kDa
Anti:Flag

Figure 3a

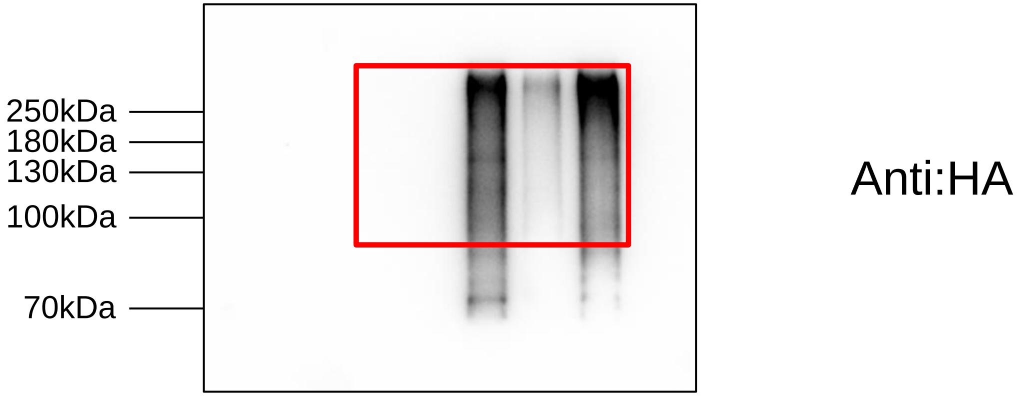
250kDa
180kDa
130kDa
100kDa
70kDa
Anti:HA

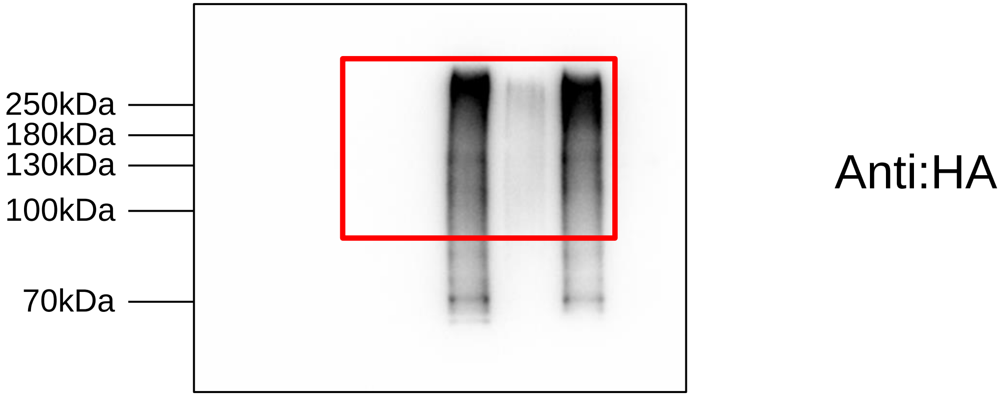
250kDa
180kDa
130kDa
100kDa
70kDa
Anti:HA

Figure 3a

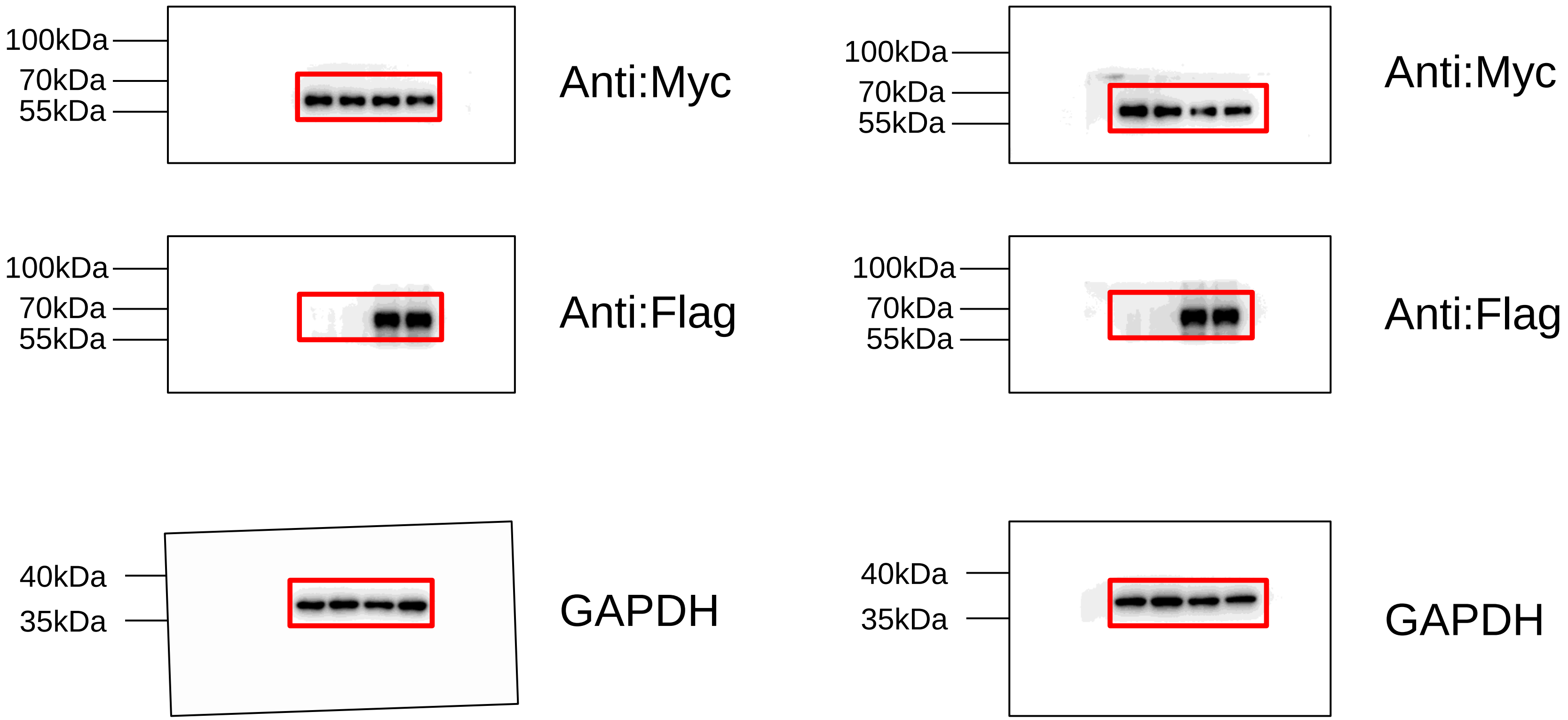
100kDa
70kDa
55kDa
Anti:Myc
100kDa
70kDa
55kDa
Anti:Myc
100kDa
70kDa
55kDa
Anti:Flag
100kDa
70kDa
55kDa
Anti:Flag
40kDa
35kDa
GAPDH
40kDa
35kDa
GAPDH

Figure 3b

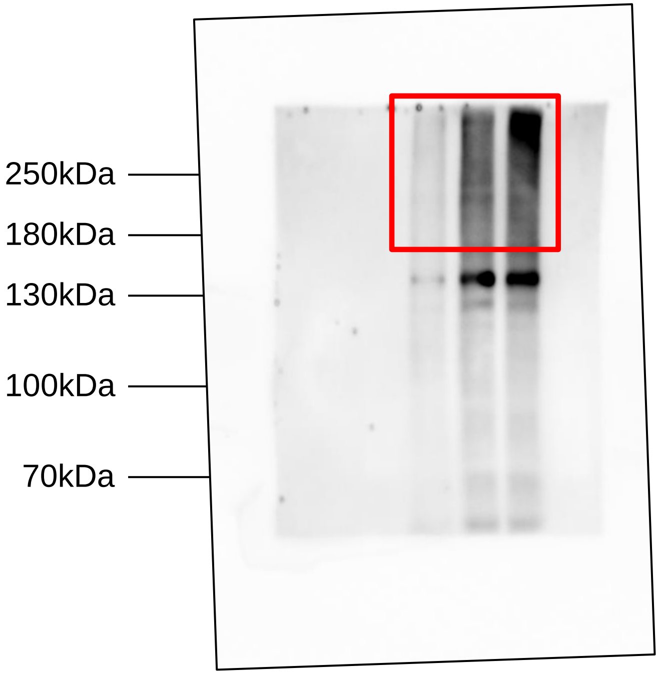

Anti:HA

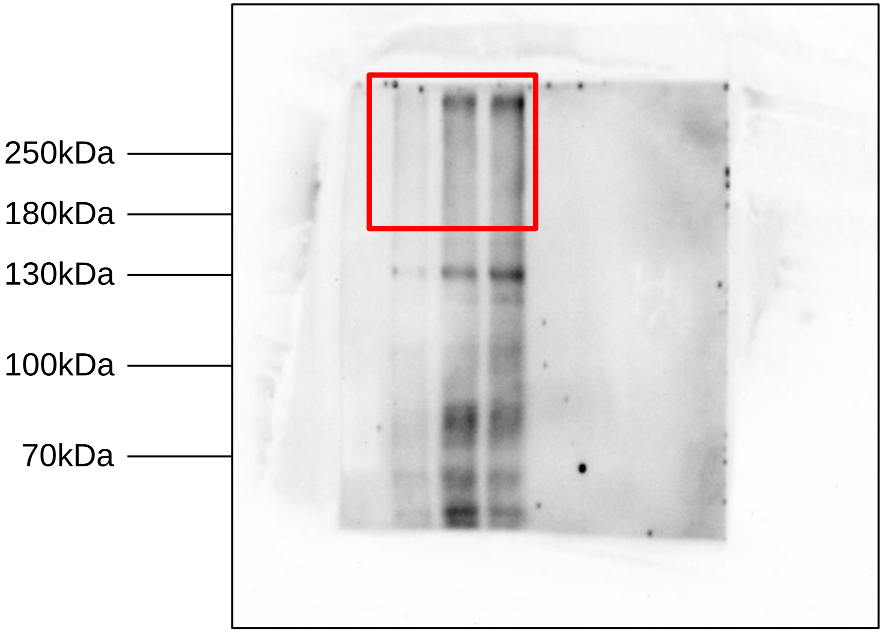

Anti:HA

Figure 3b

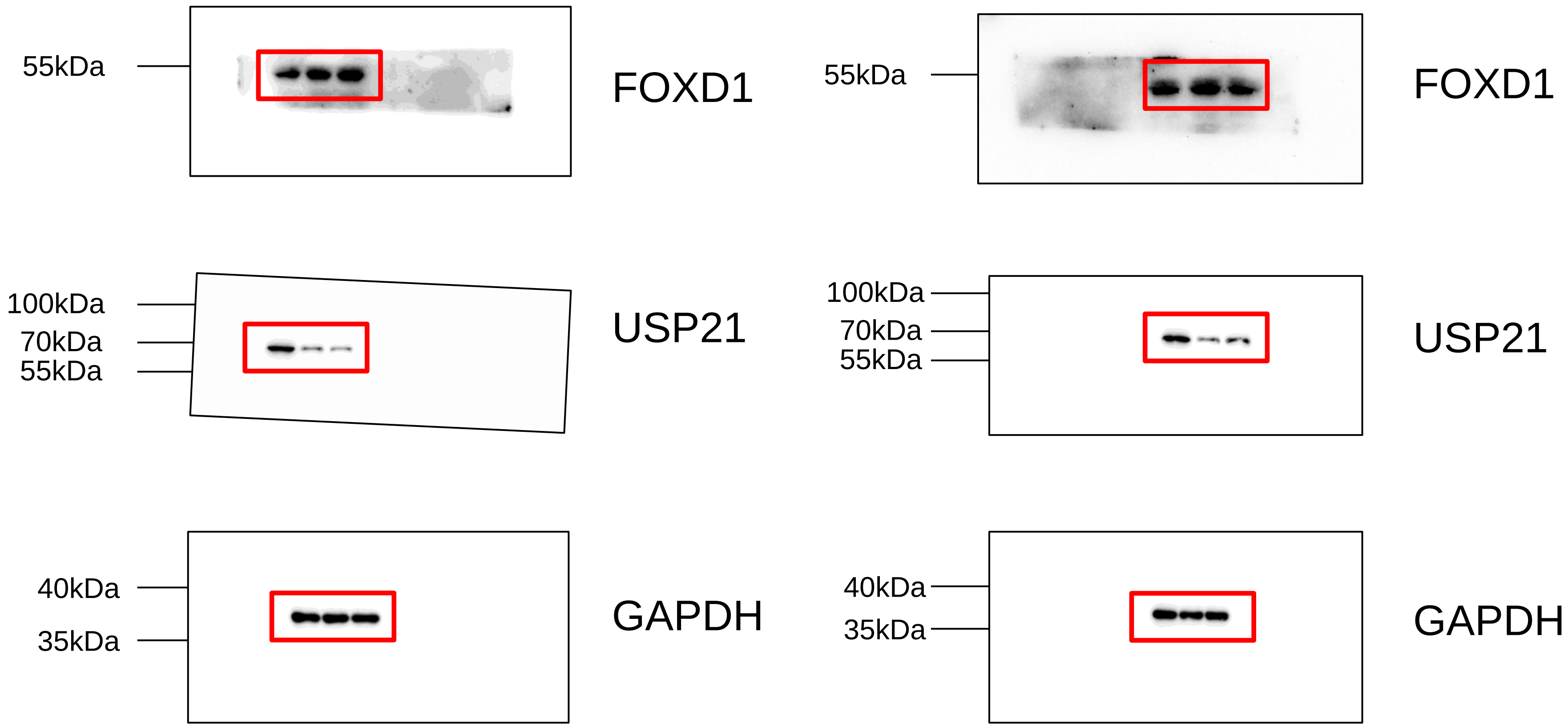
55kDa
FOXD1
55kDa
FOXD1
100kDa
70kDa
55kDa
USP21
100kDa
70kDa
55kDa
USP21
40kDa
35kDa
GAPDH
40kDa
35kDa
GAPDH

Figure 3c

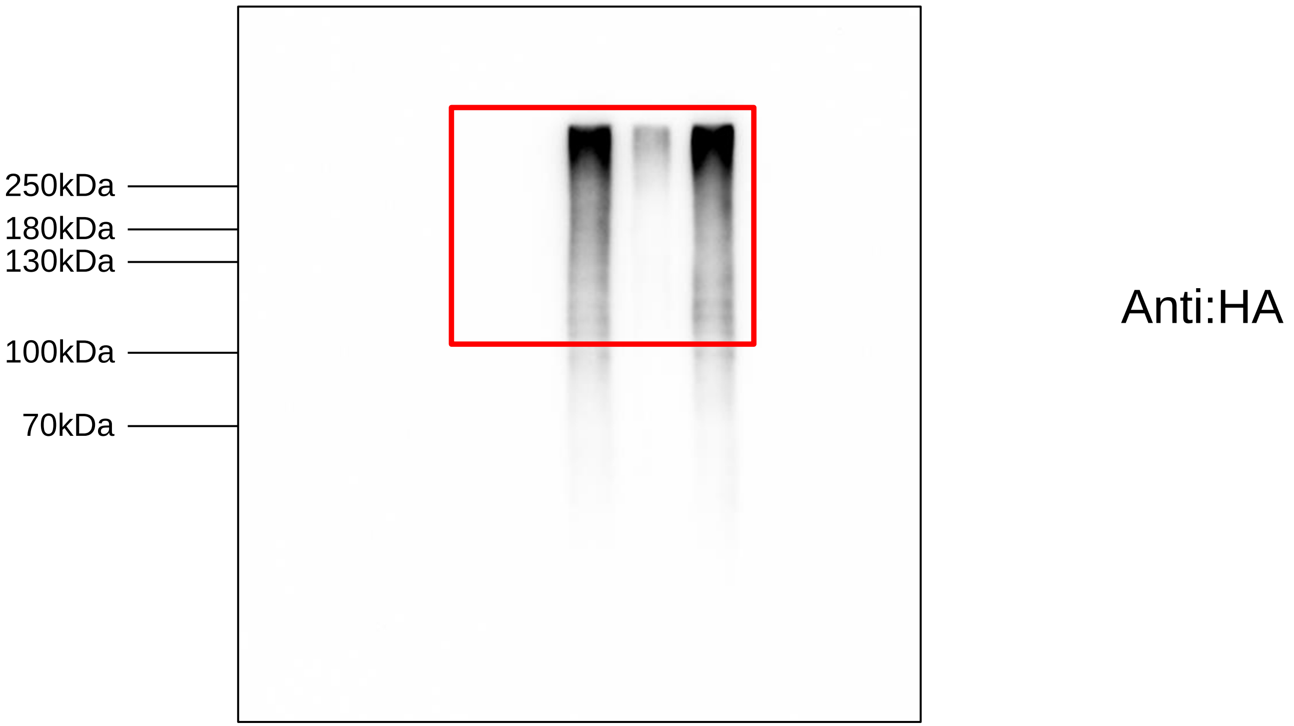

Figure 3c

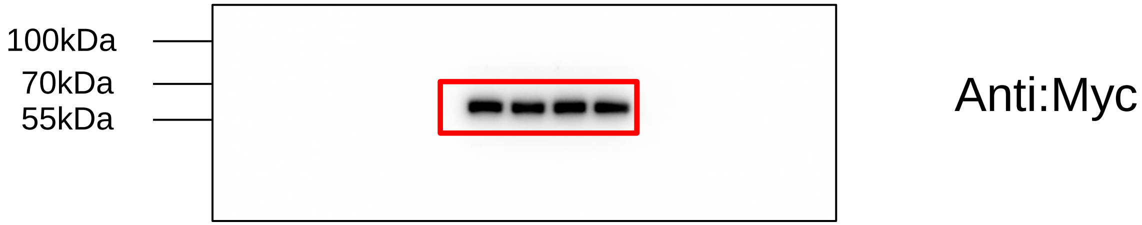

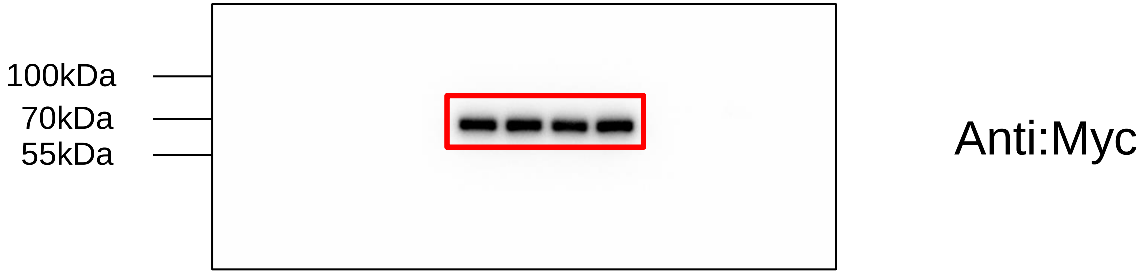

Figure 3d

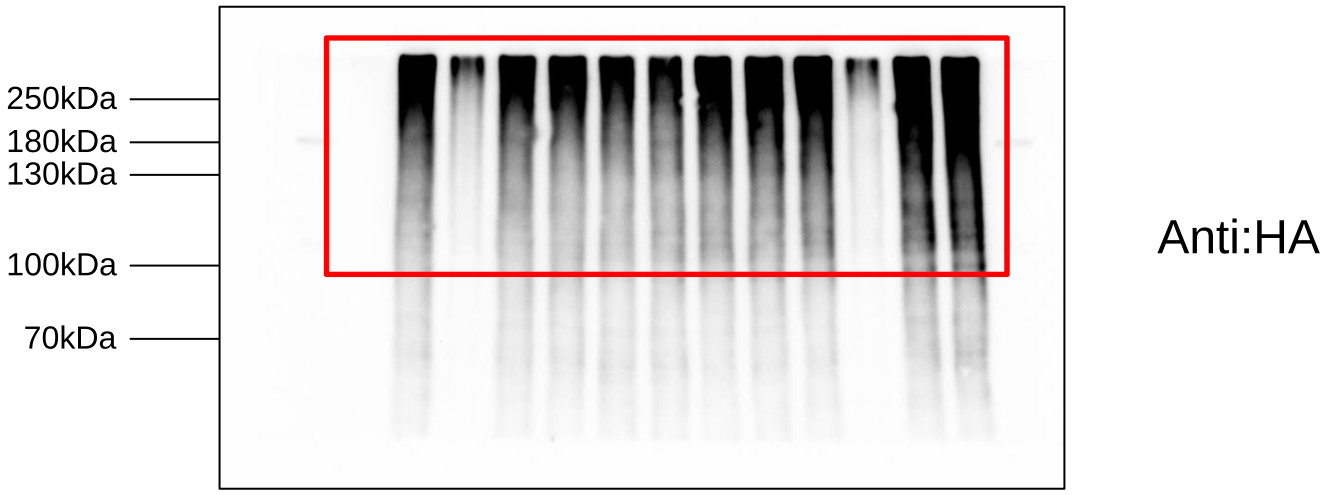

Figure 3d

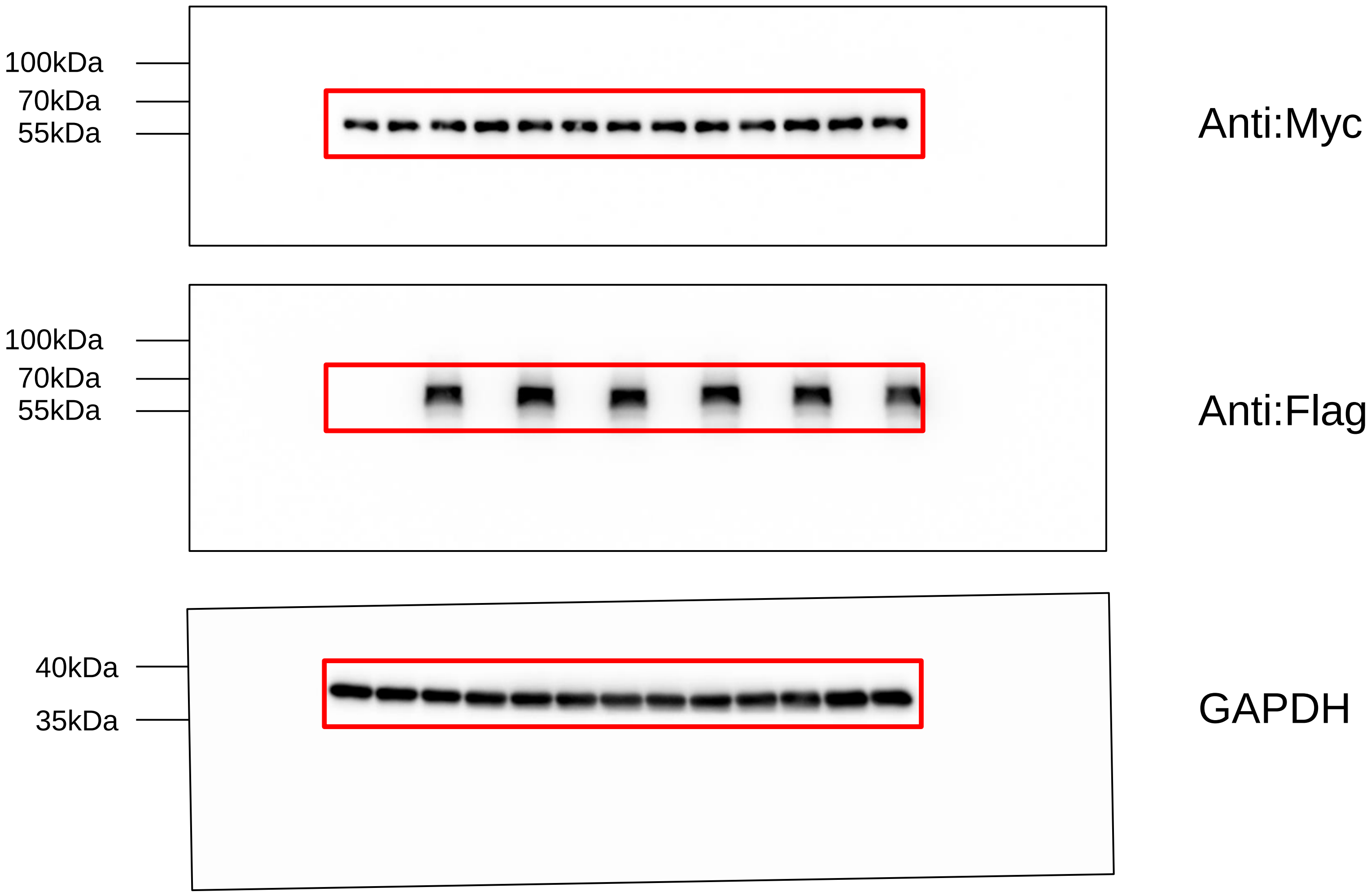
100kDa
70kDa
55kDa
Anti:Myc
100kDa
70kDa
55kDa
Anti:Flag
40kDa
35kDa
GAPDH

Figure 3d

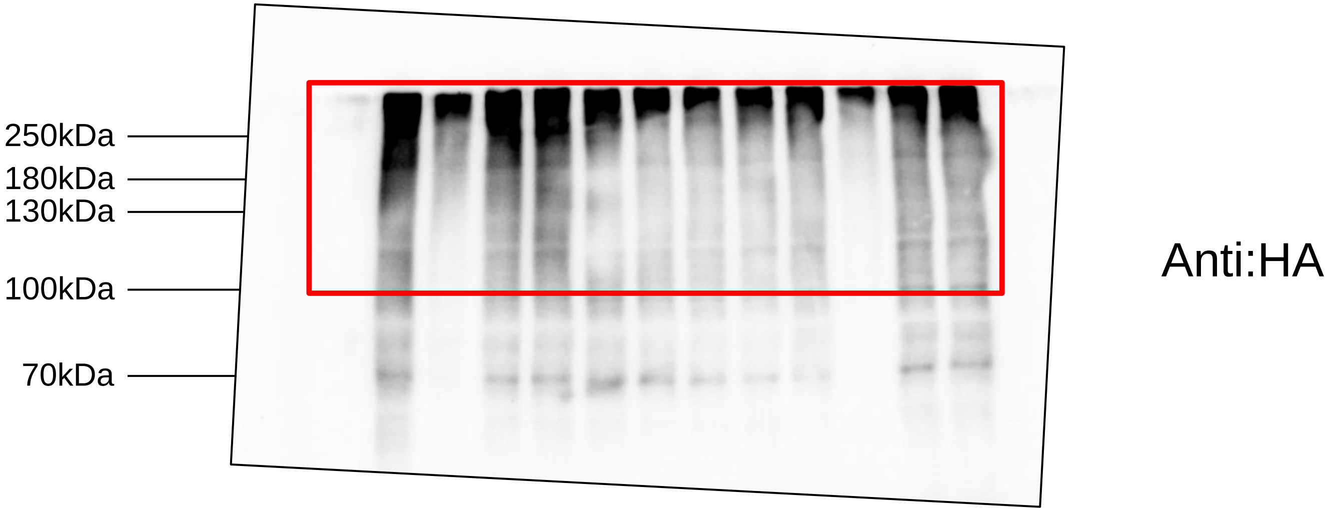

Figure 3d

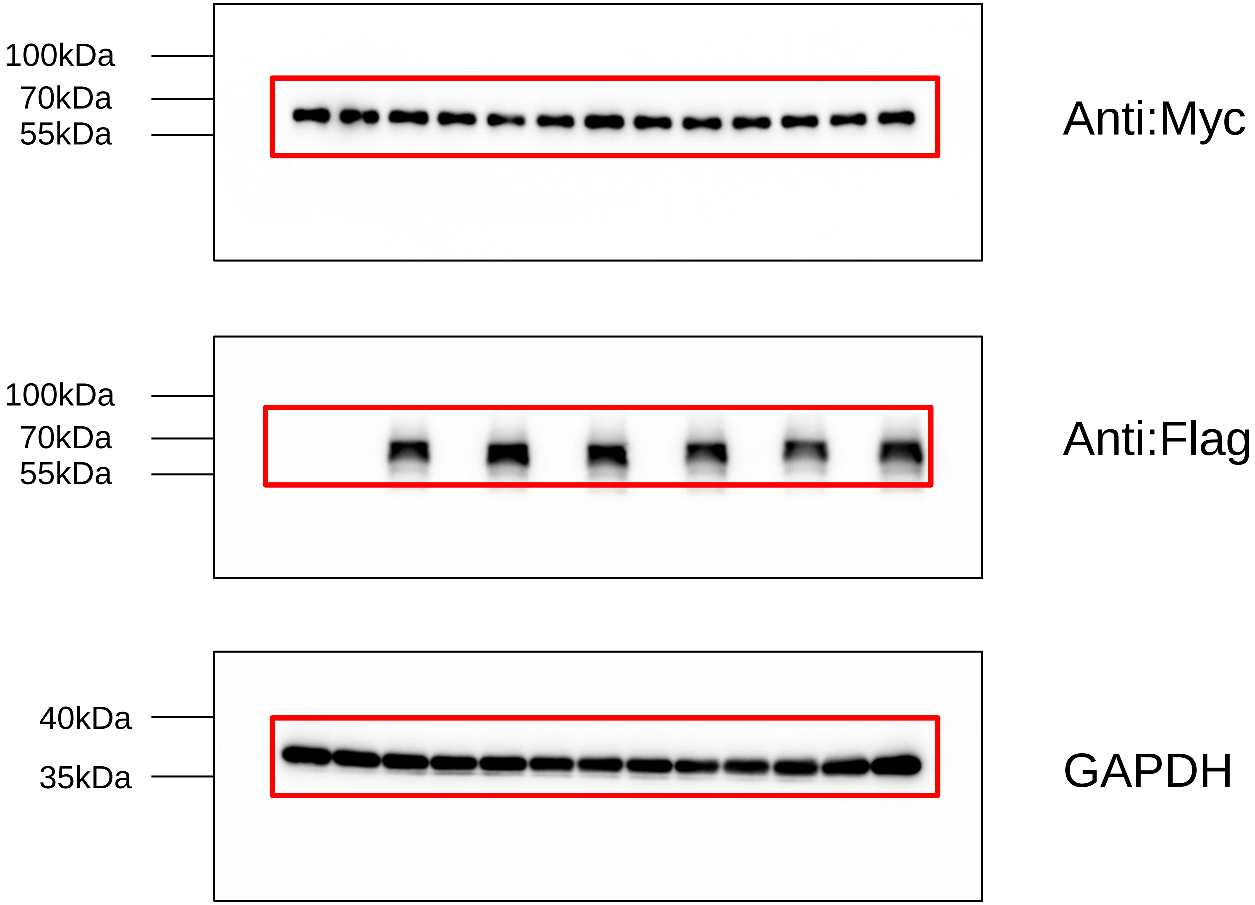

100kDa
70kDa
55kDa
Anti:Myc
100kDa
70kDa
55kDa
Anti:Flag
40kDa
35kDa
GAPDH

Figure 3e

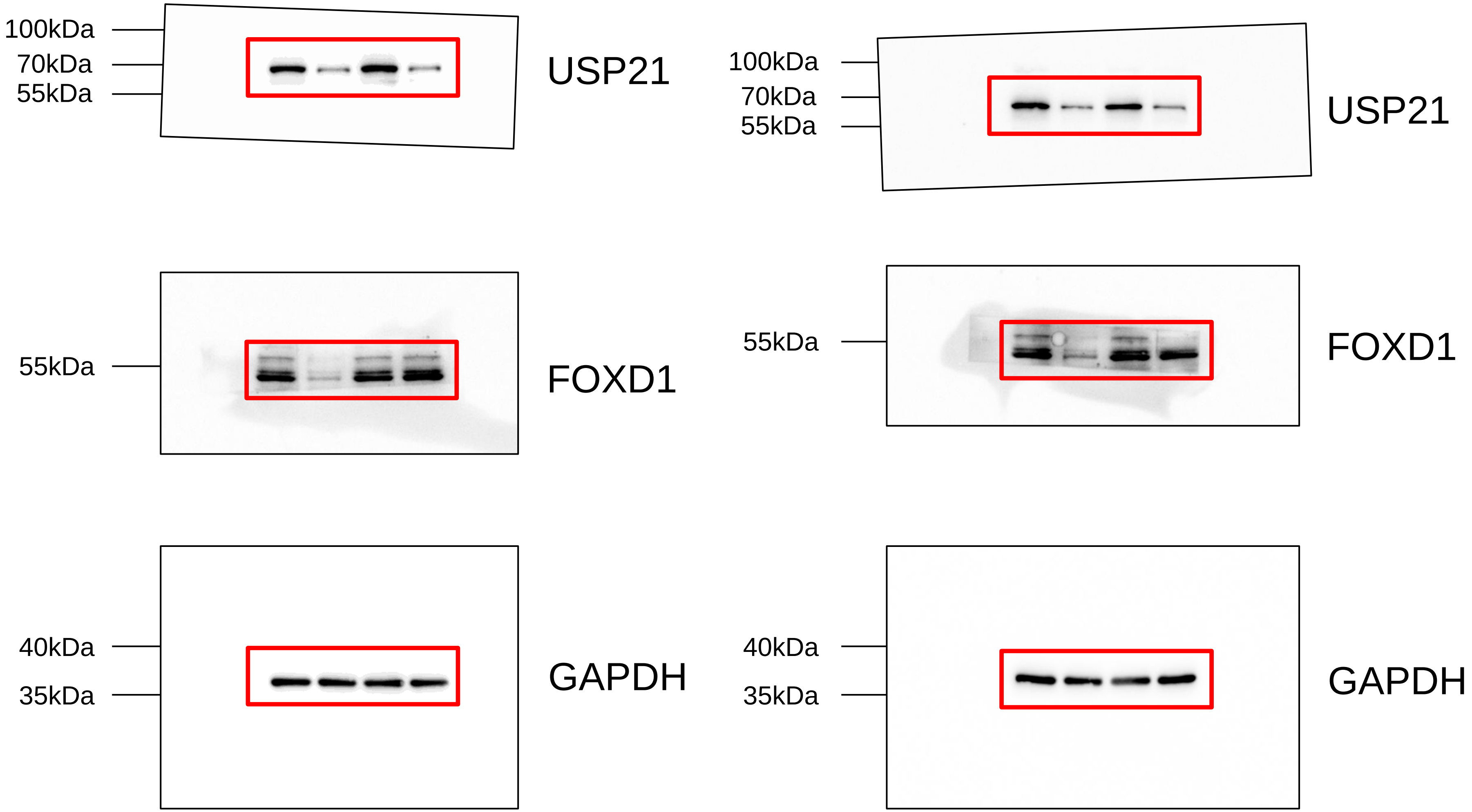
100kDa
70kDa
55kDa
USP21
100kDa
70kDa
55kDa
USP21
55kDa
FOXD1
55kDa
FOXD1
40kDa
35kDa
GAPDH
40kDa
35kDa
GAPDH

Figure 4e

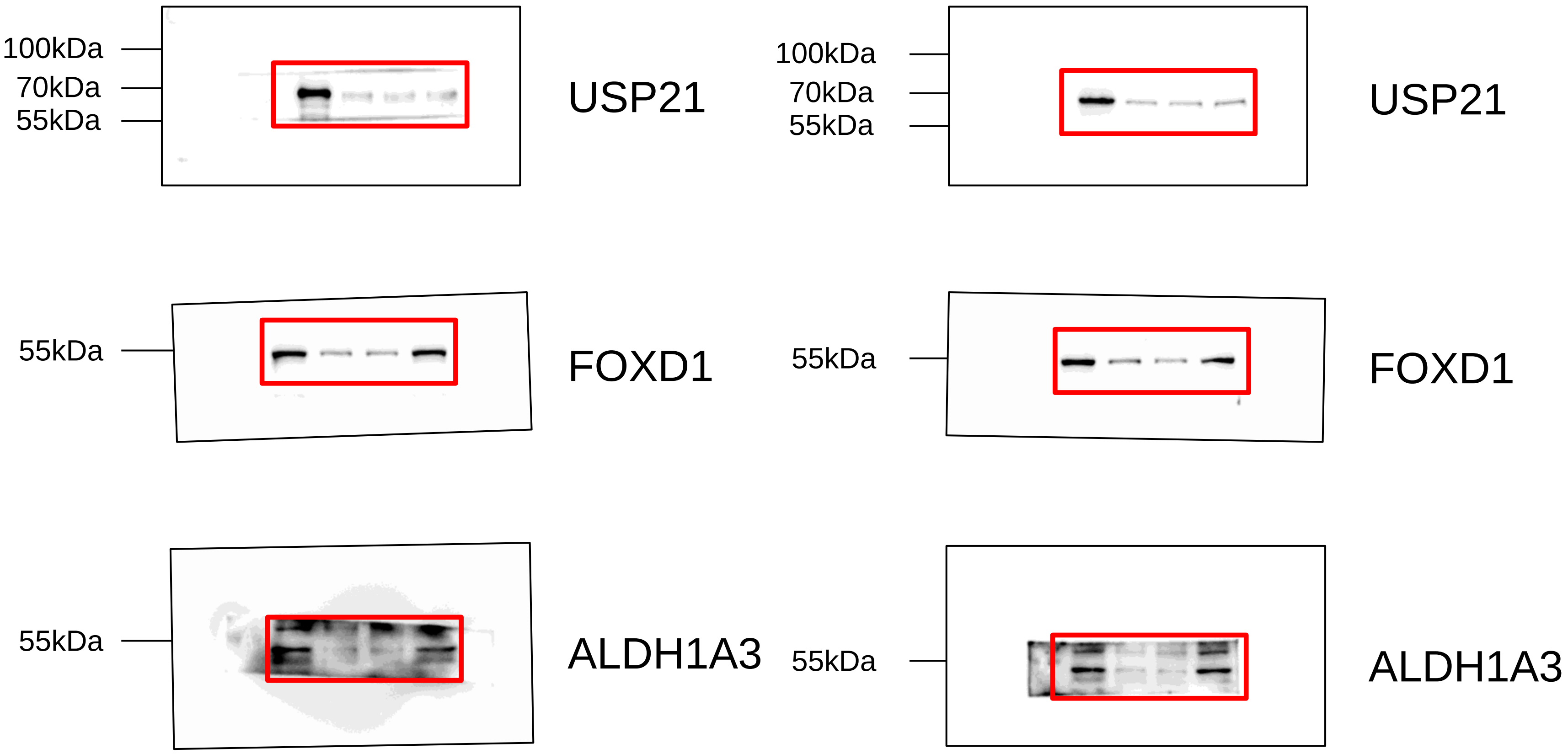
100kDa
70kDa
55kDa
USP21
100kDa
70kDa
55kDa
USP21
55kDa
FOXD1
55kDa
FOXD1
55kDa
ALDH1A3
55kDa
ALDH1A3

Figure 4e

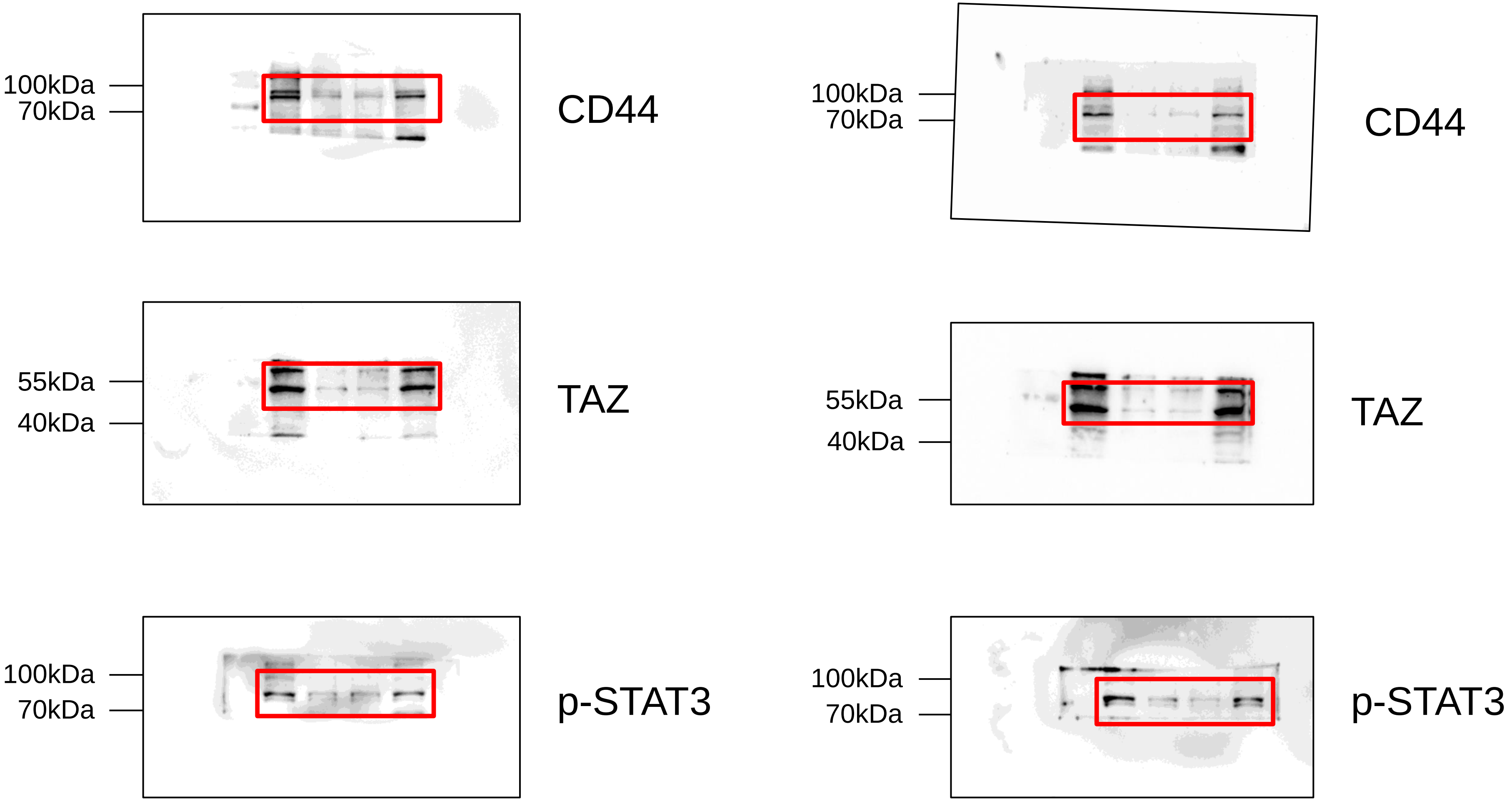
100kDa
70kDa
CD44
100kDa
70kDa
CD44
55kDa
40kDa
TAZ
55kDa
40kDa
TAZ
100kDa
70kDa
p-STAT3
100kDa
70kDa
p-STAT3

Figure 4e

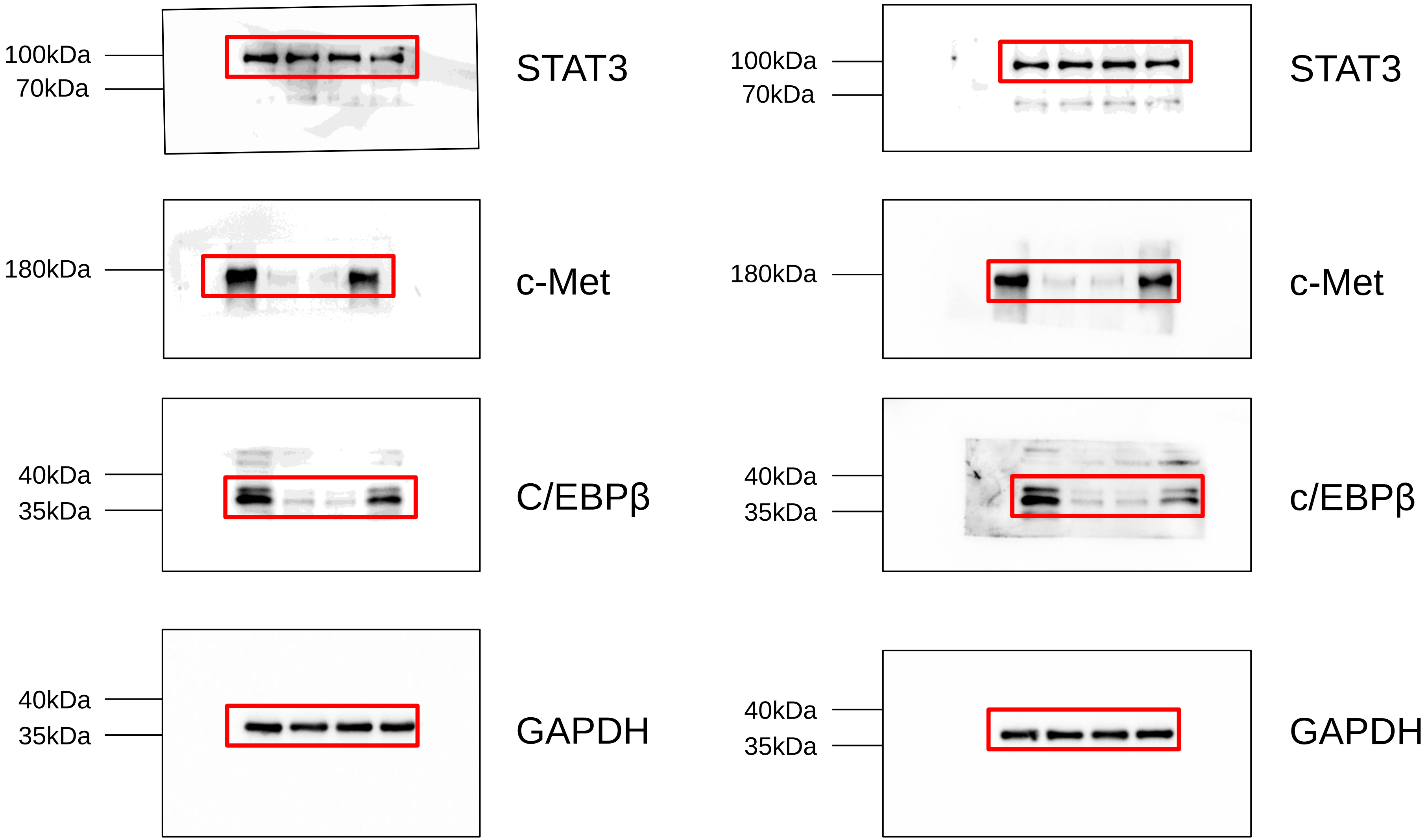
100kDa
70kDa
STAT3
100kDa
70kDa
STAT3
180kDa
c-Met
180kDa
c-Met
40kDa
35kDa
C/EBPβ
40kDa
35kDa
c/EBPβ
40kDa
35kDa
GAPDH
40kDa
35kDa
GAPDH

Figure 5a

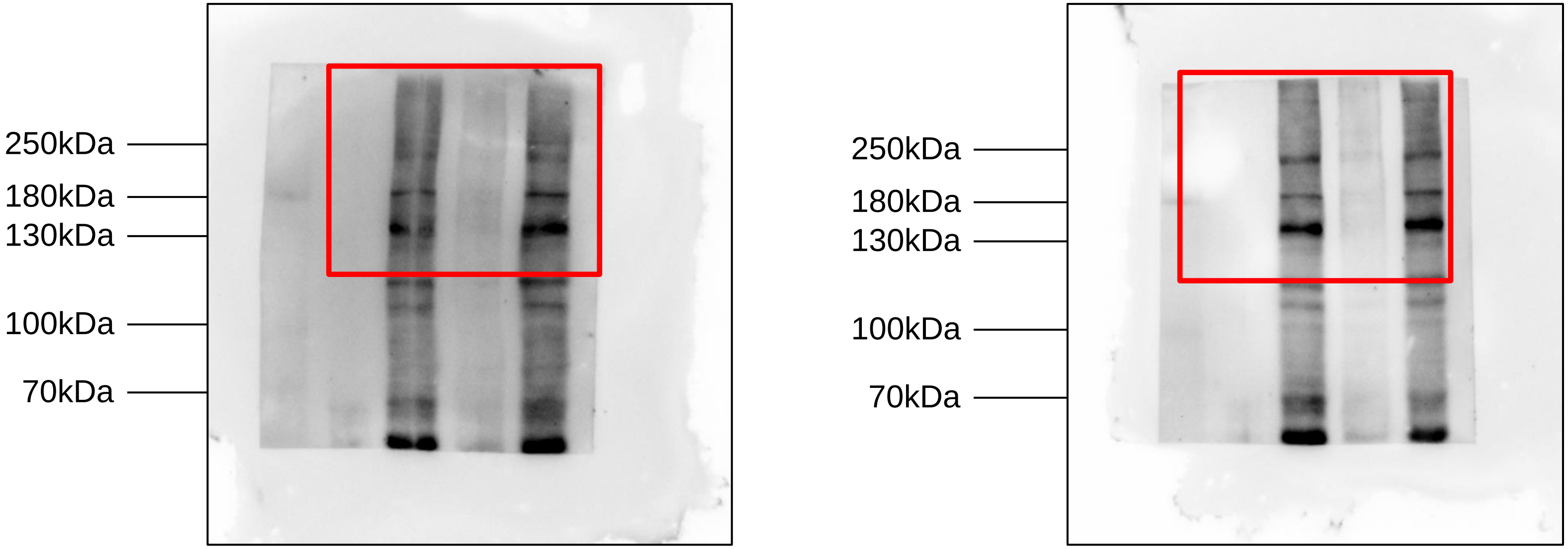

Anti:HA

Anti:HA

Figure 5a

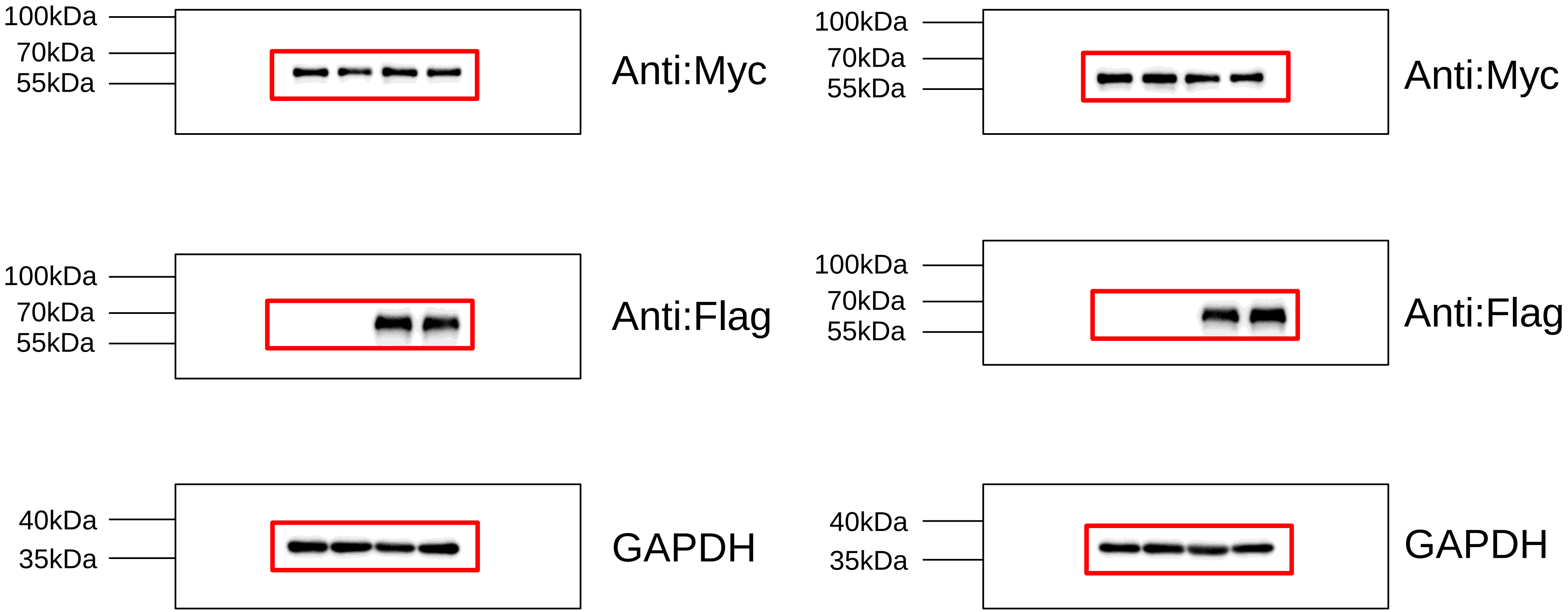

100kDa
70kDa
55kDa
Anti:Myc
100kDa
70kDa
55kDa
Anti:Myc
100kDa
70kDa
55kDa
Anti:Flag
100kDa
70kDa
55kDa
Anti:Flag
40kDa
35kDa
GAPDH
40kDa
35kDa
GAPDH

Figure 5b

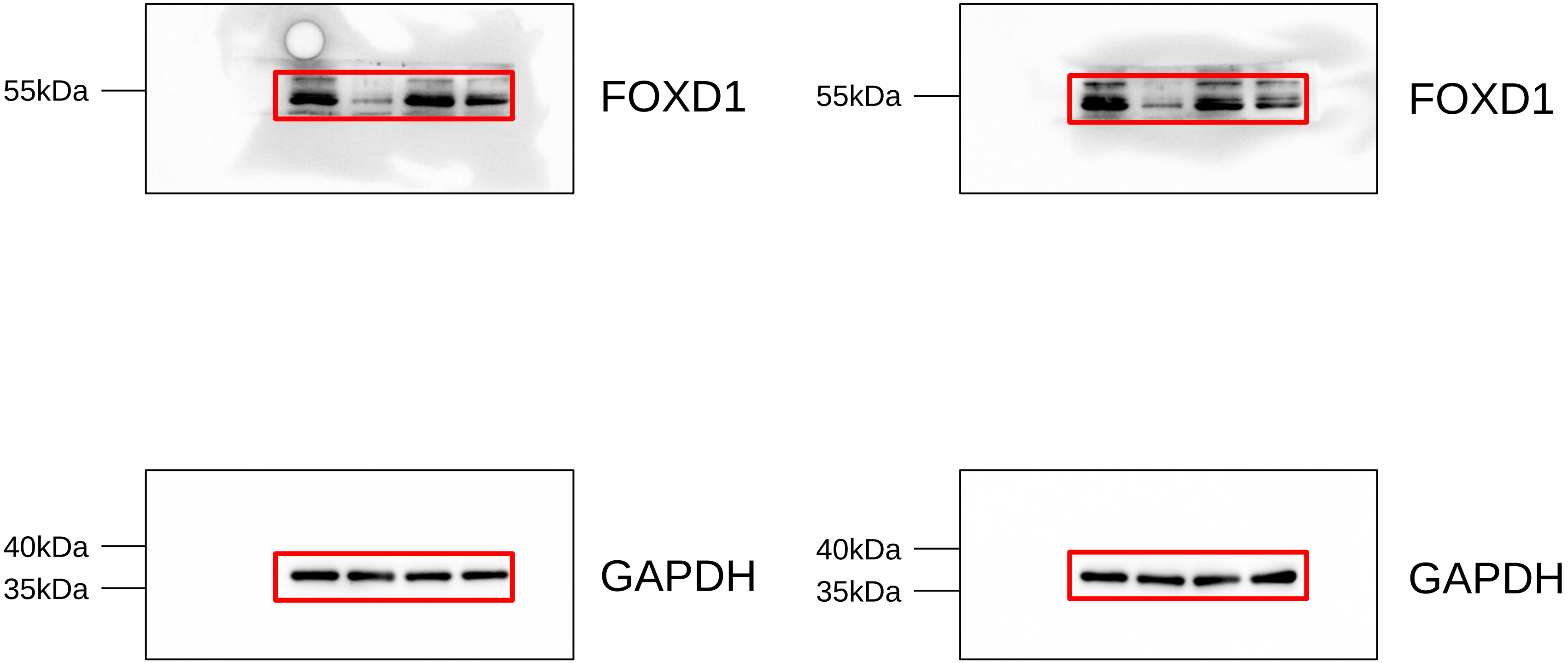

55kDa
FOXD1
55kDa
FOXD1
40kDa
35kDa
GAPDH
40kDa
35kDa
GAPDH

Figure 5c

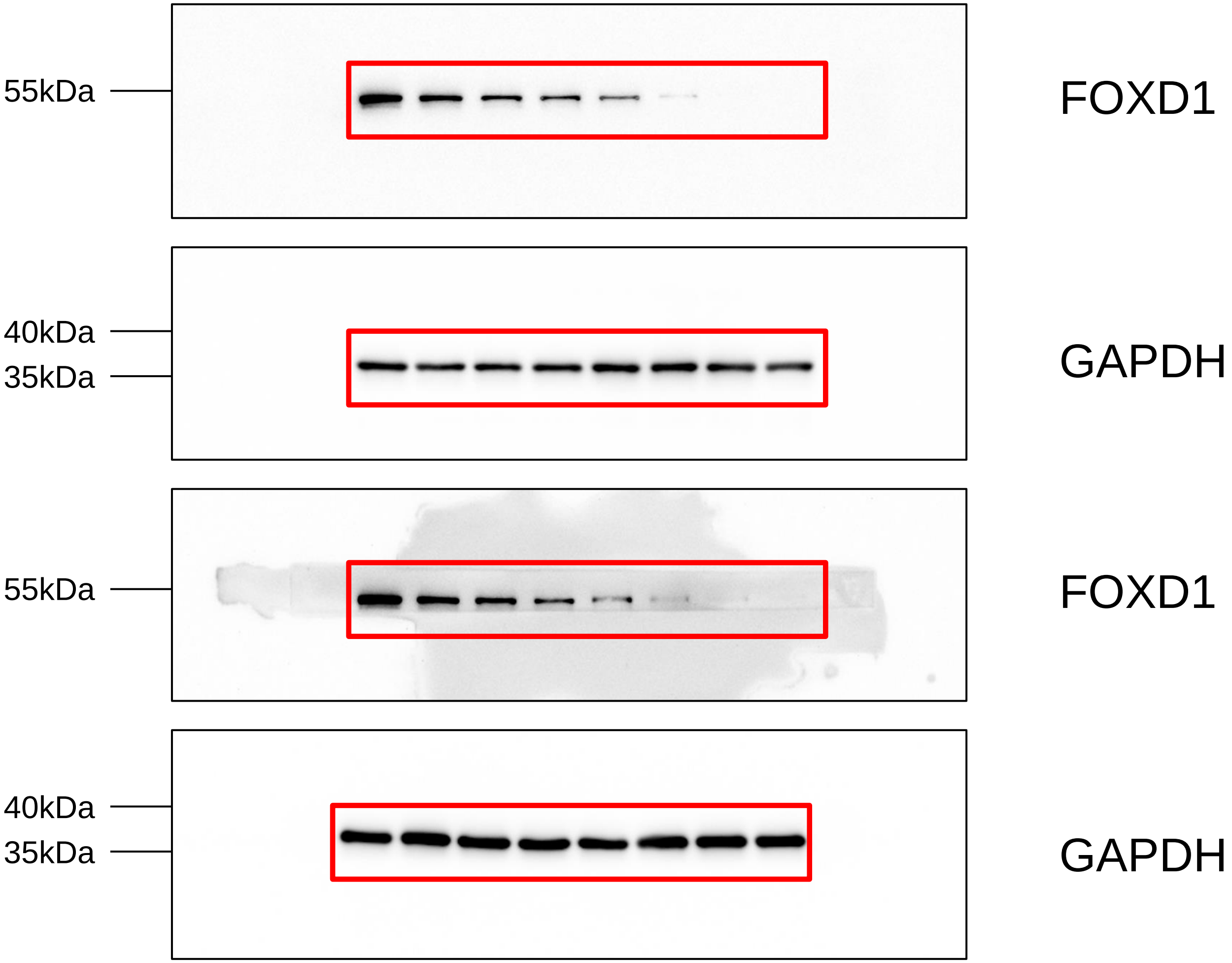
55kDa
FOXD1
40kDa
35kDa
GAPDH
55kDa
FOXD1
40kDa
35kDa
GAPDH

Figure 5d

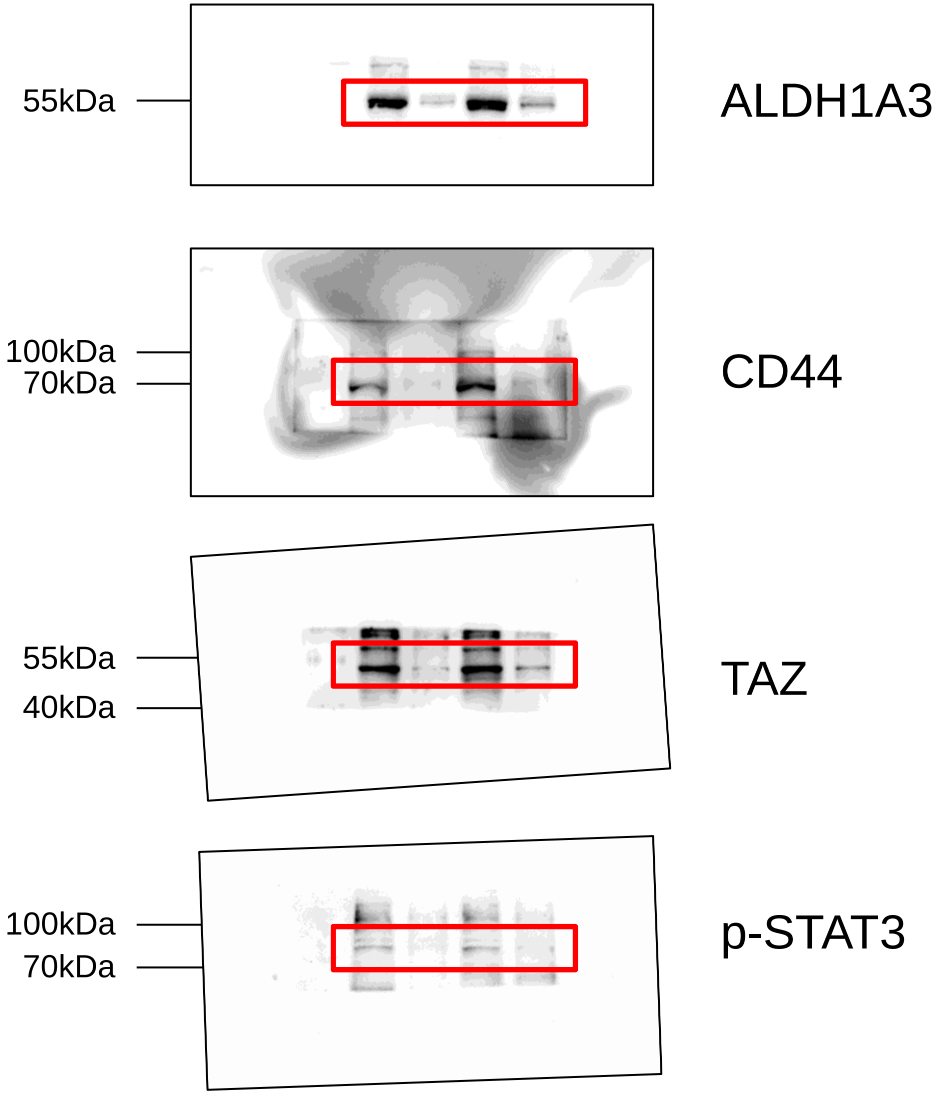

55kDa
ALDH1A3
100kDa
70kDa
CD44
55kDa
40kDa
TAZ
100kDa
70kDa
p-STAT3

Figure 5d

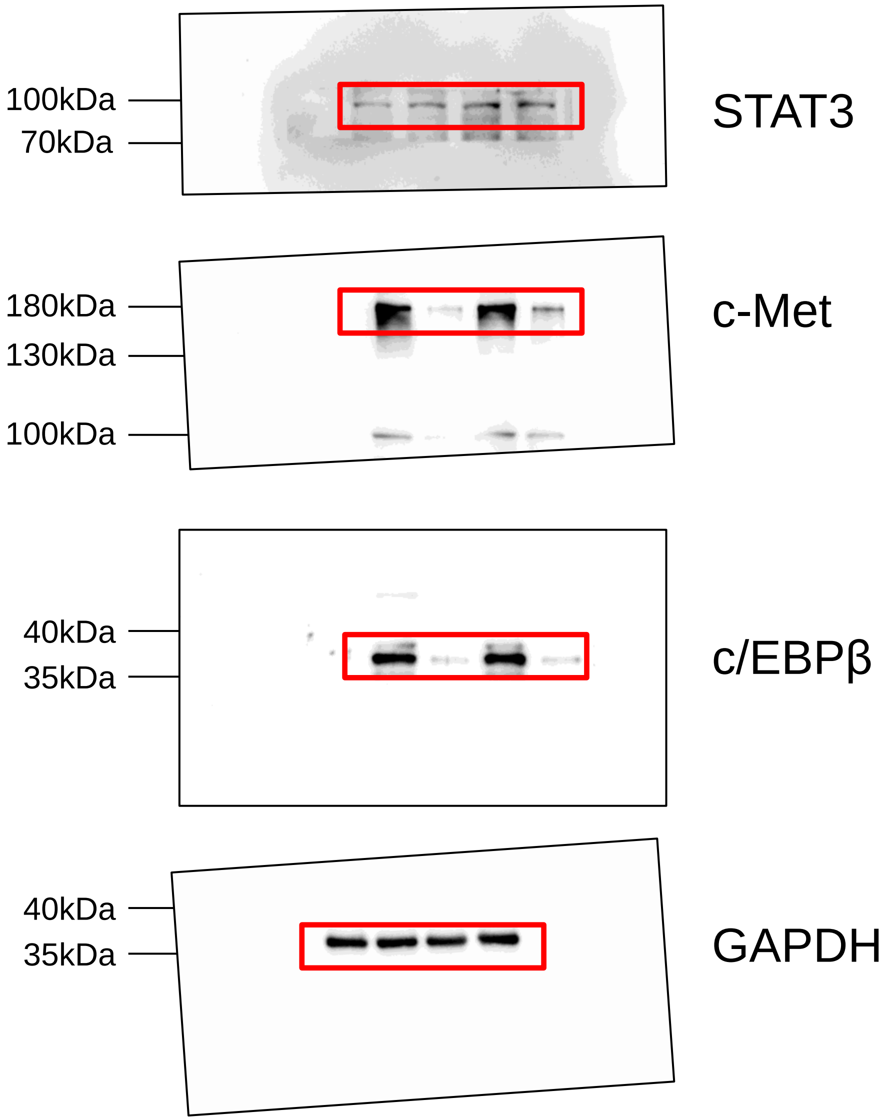
100kDa
70kDa
STAT3
180kDa
130kDa
100kDa
c-Met
40kDa
35kDa
c/EBPβ
40kDa
35kDa
GAPDH

Supplemental Figure 1a

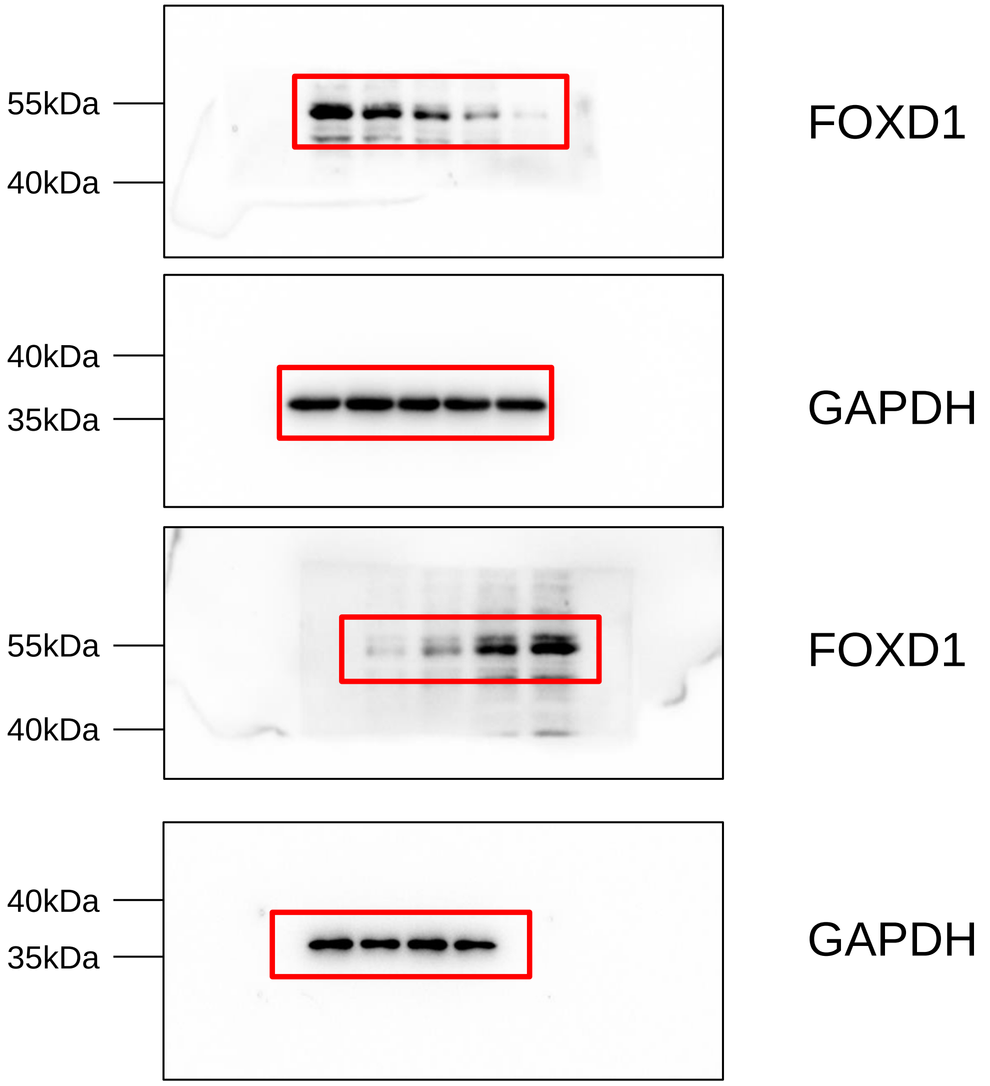

Supplemental Figure 1a

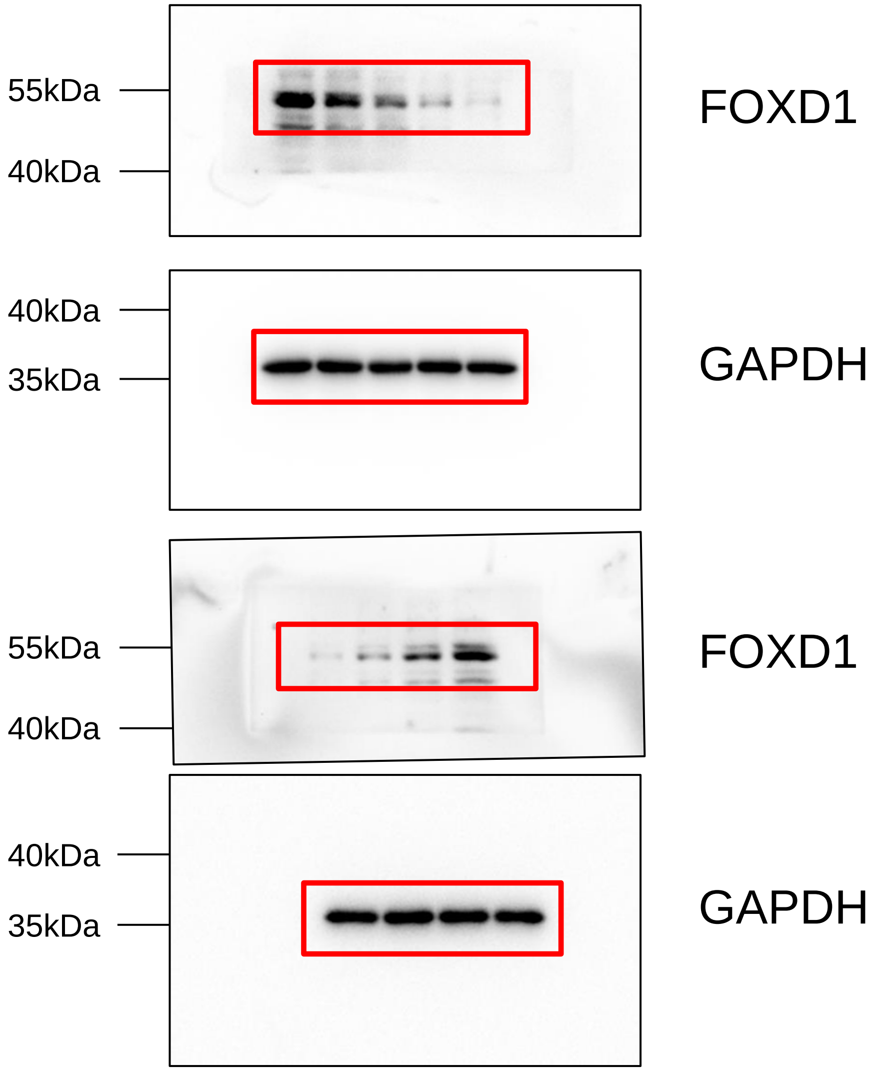

Supplemental Figure 1b

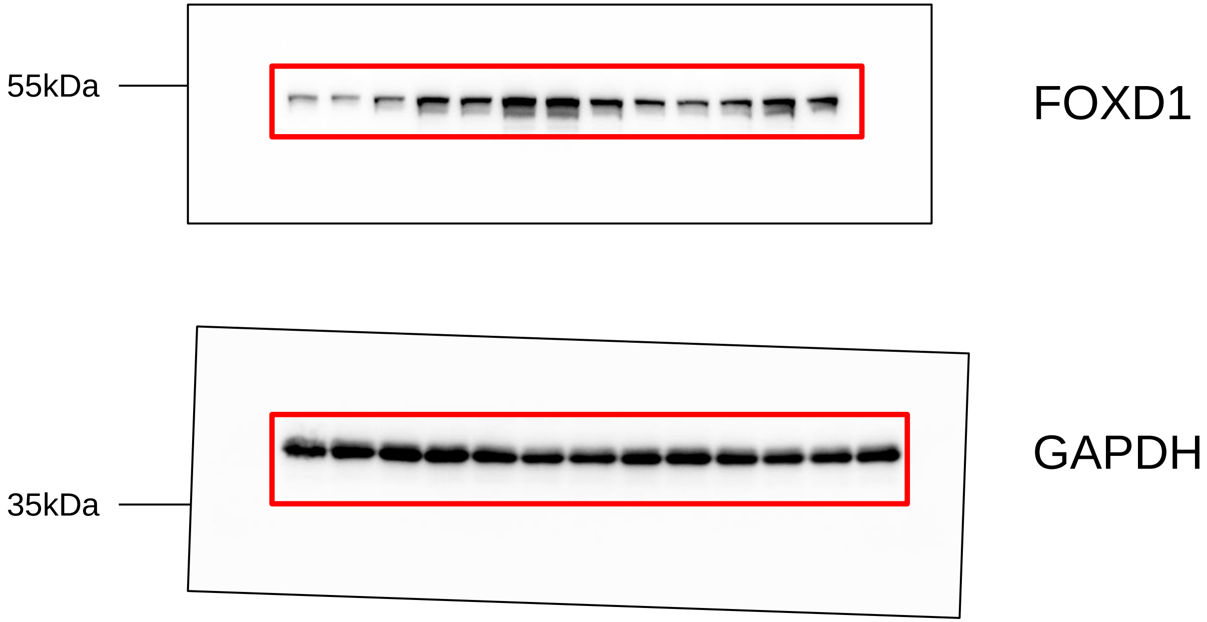

Supplemental Figure 1b

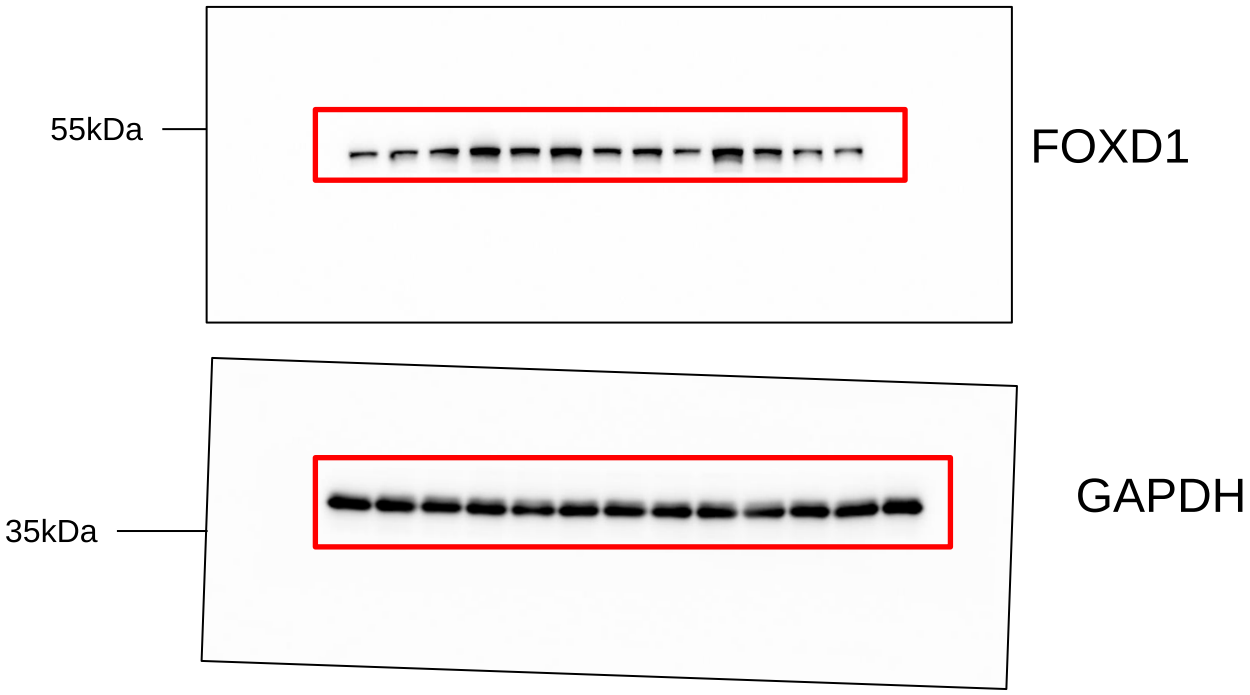

Supplemental Figure 1b

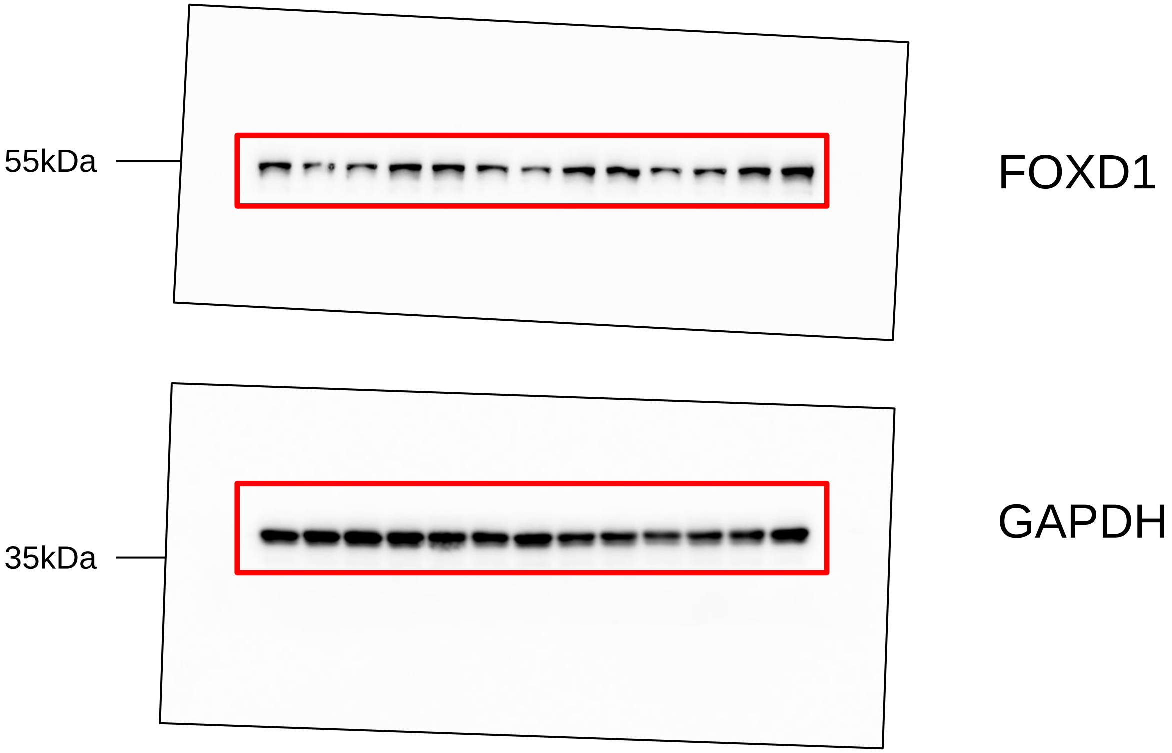

Supplemental Figure 1b

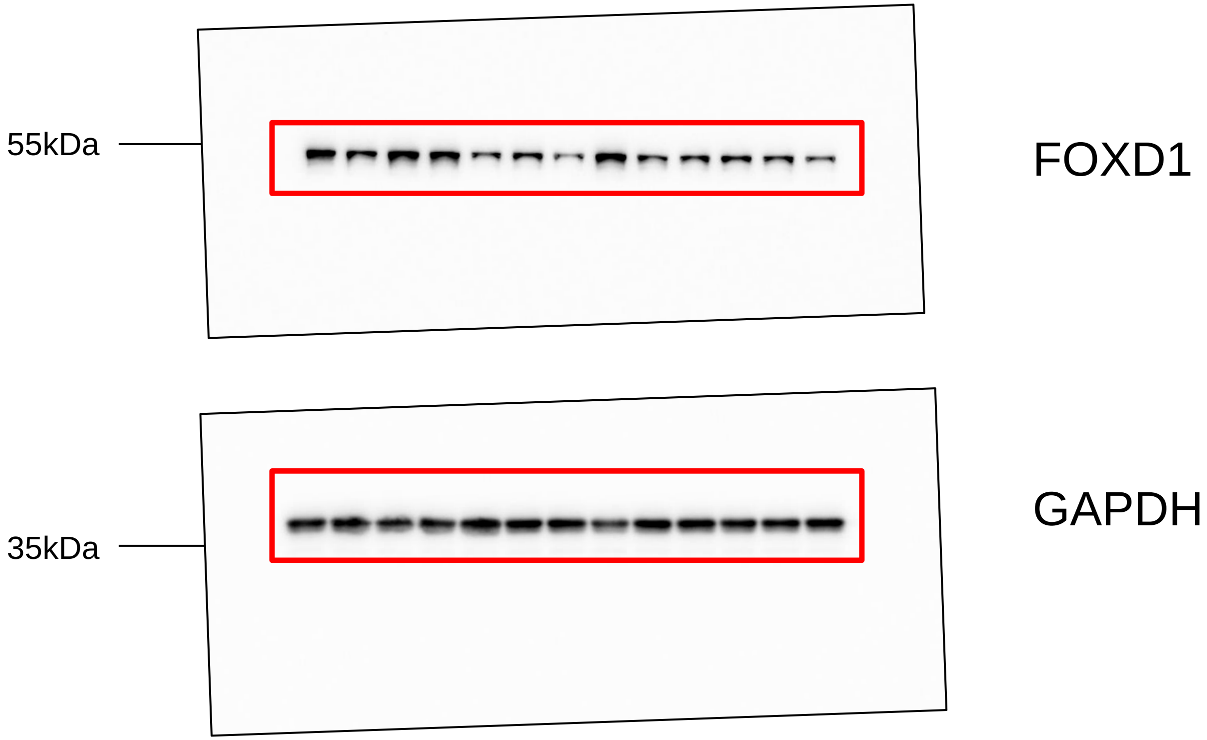

Supplemental Figure 1b

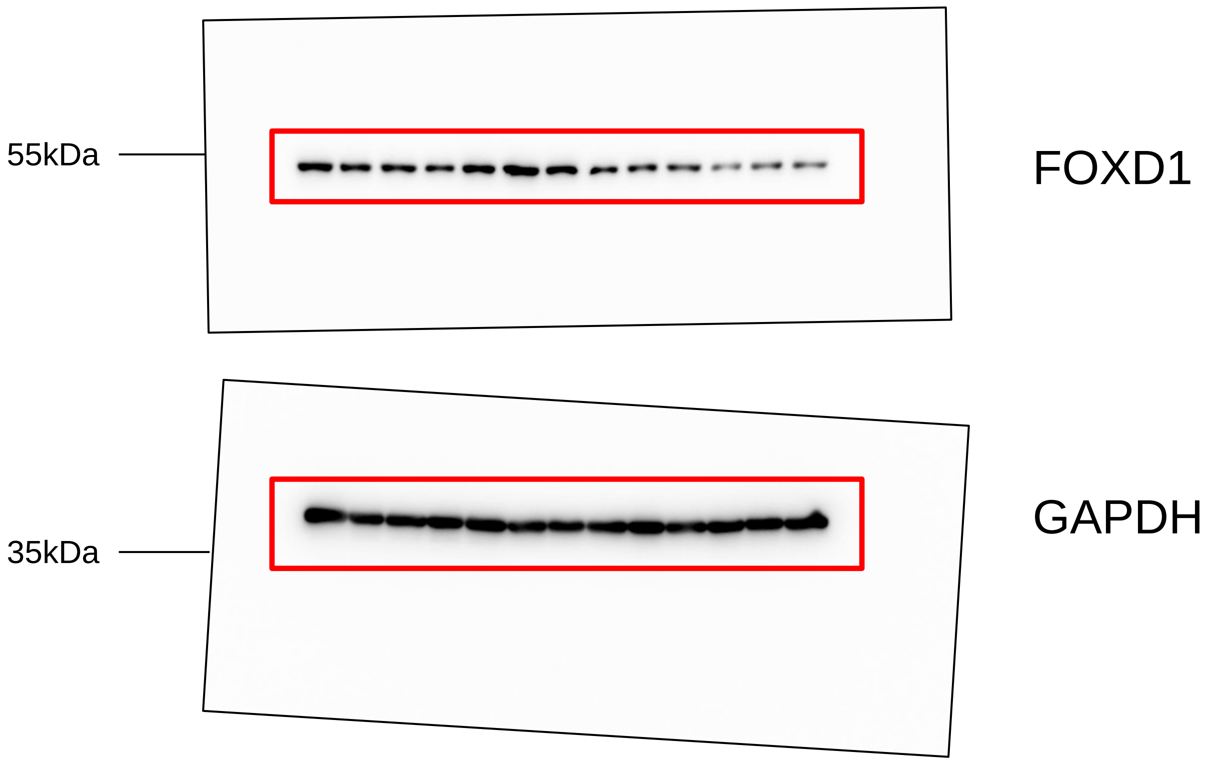

Supplemental Figure 1b

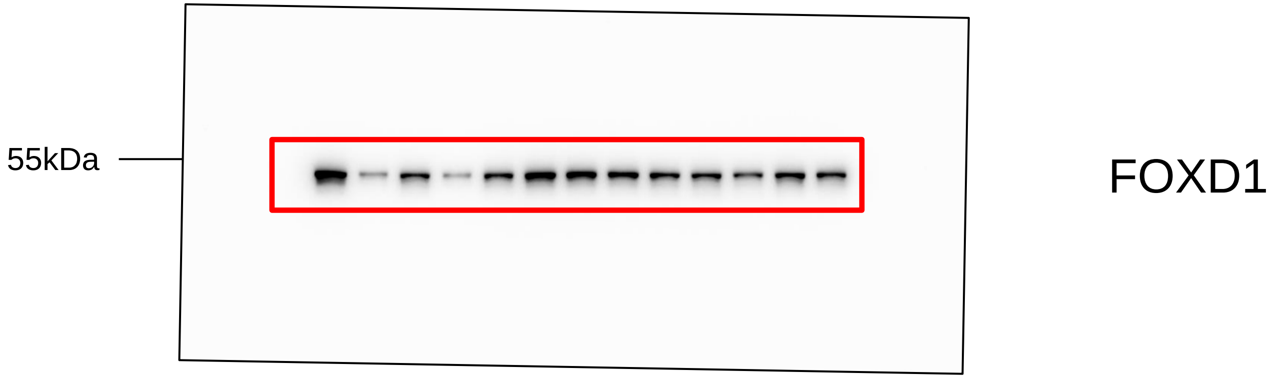

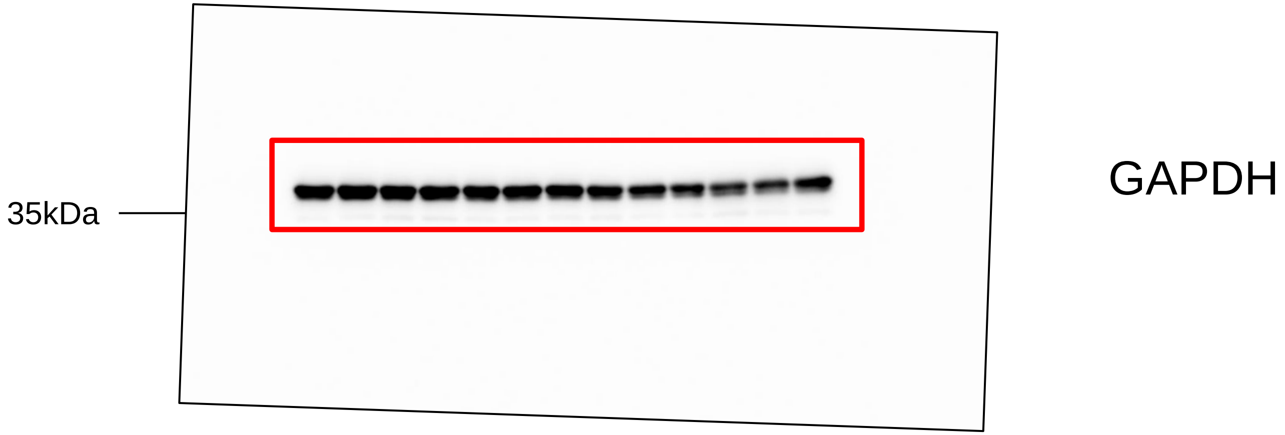

Supplemental Figure 1b

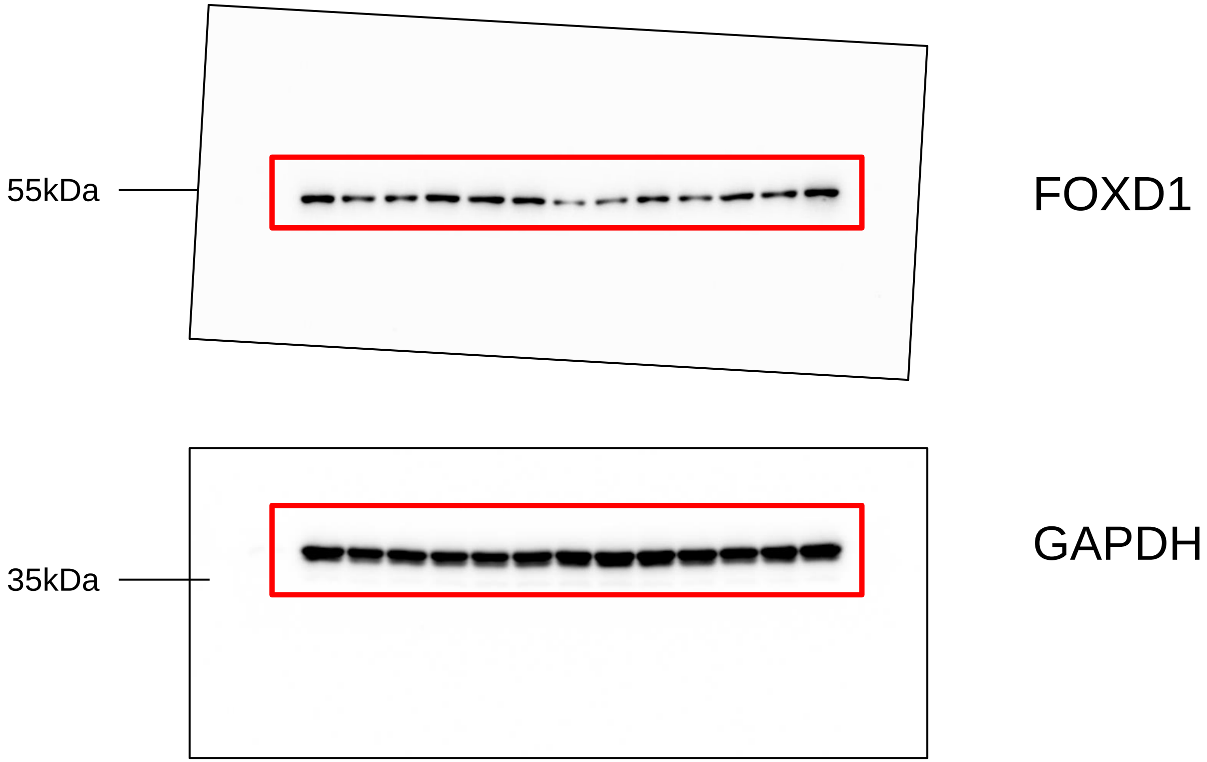

Supplemental Figure 1b

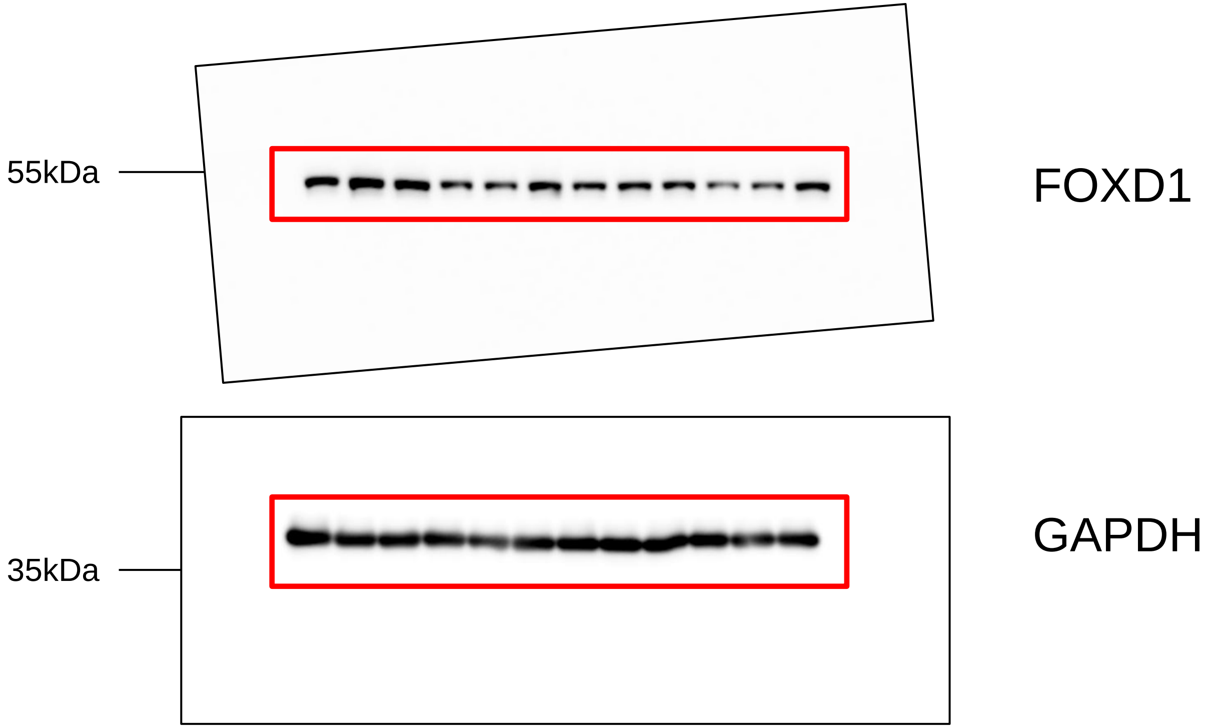

Supplemental Figure 1b

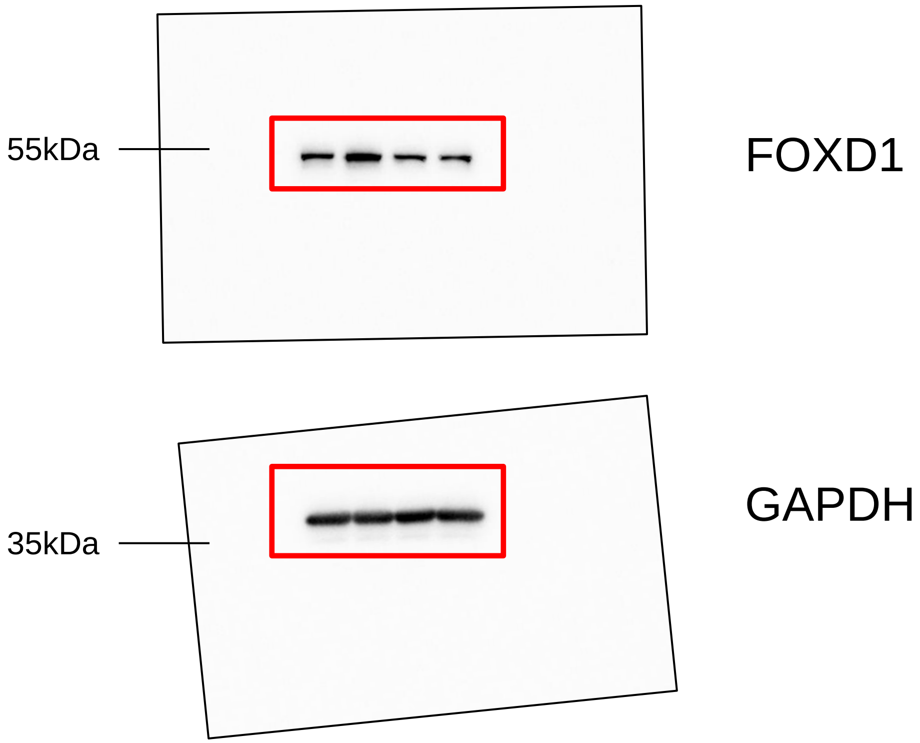

Supplemental Figure 1e

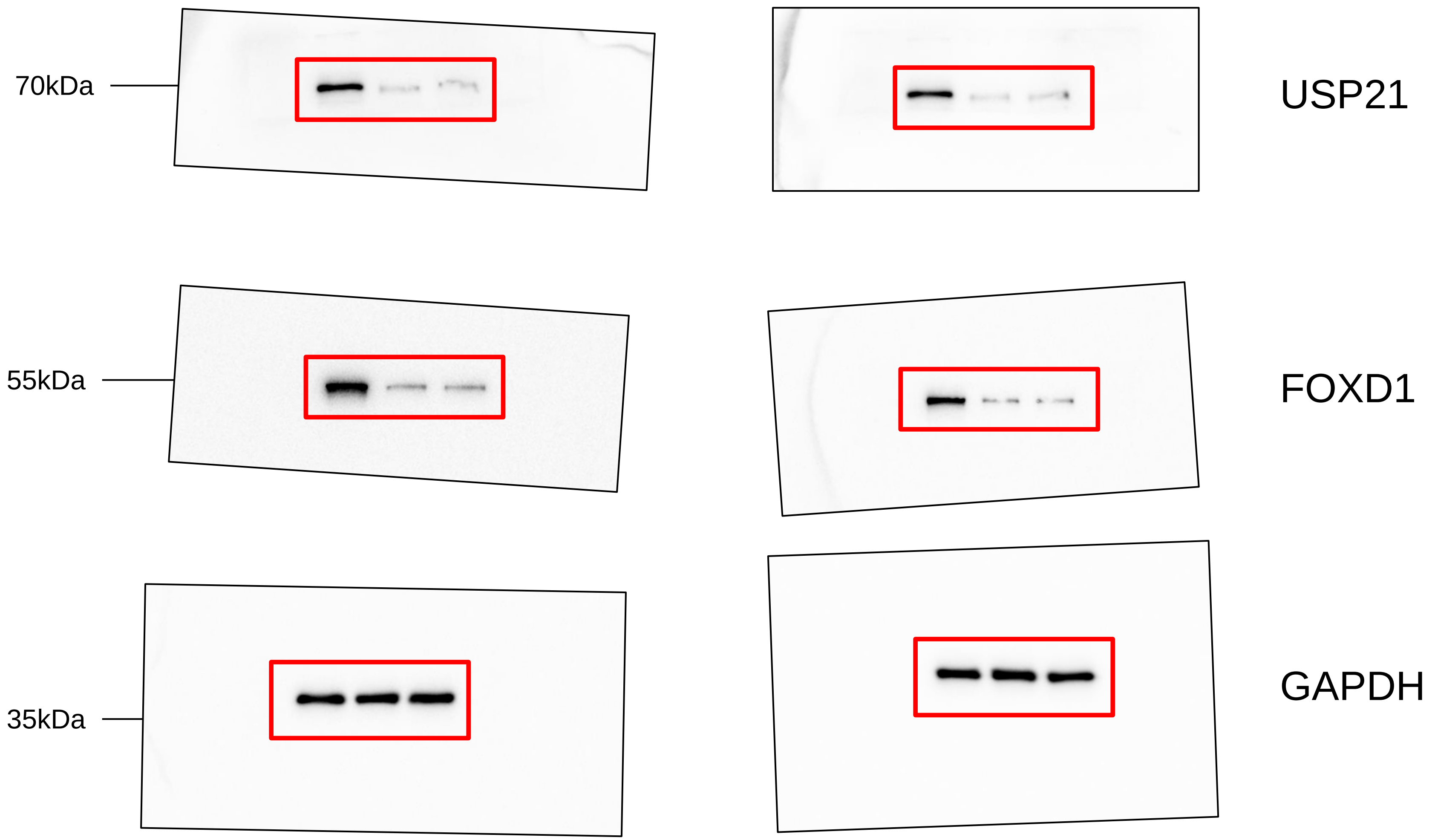

Supplemental Figure 1f

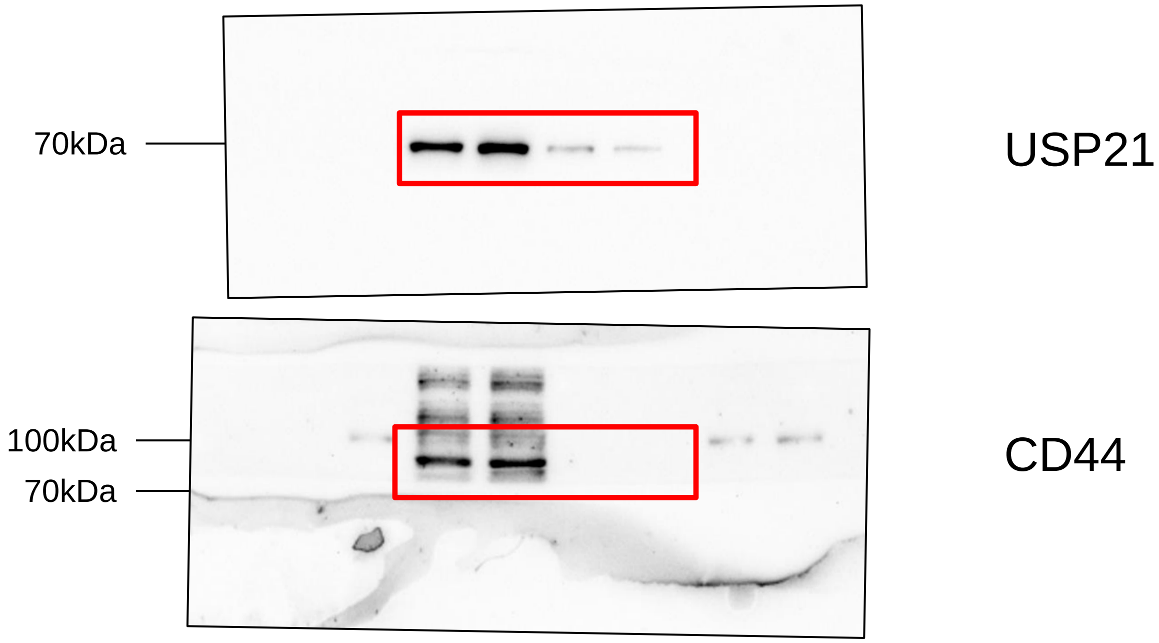

Supplemental Figure 1f

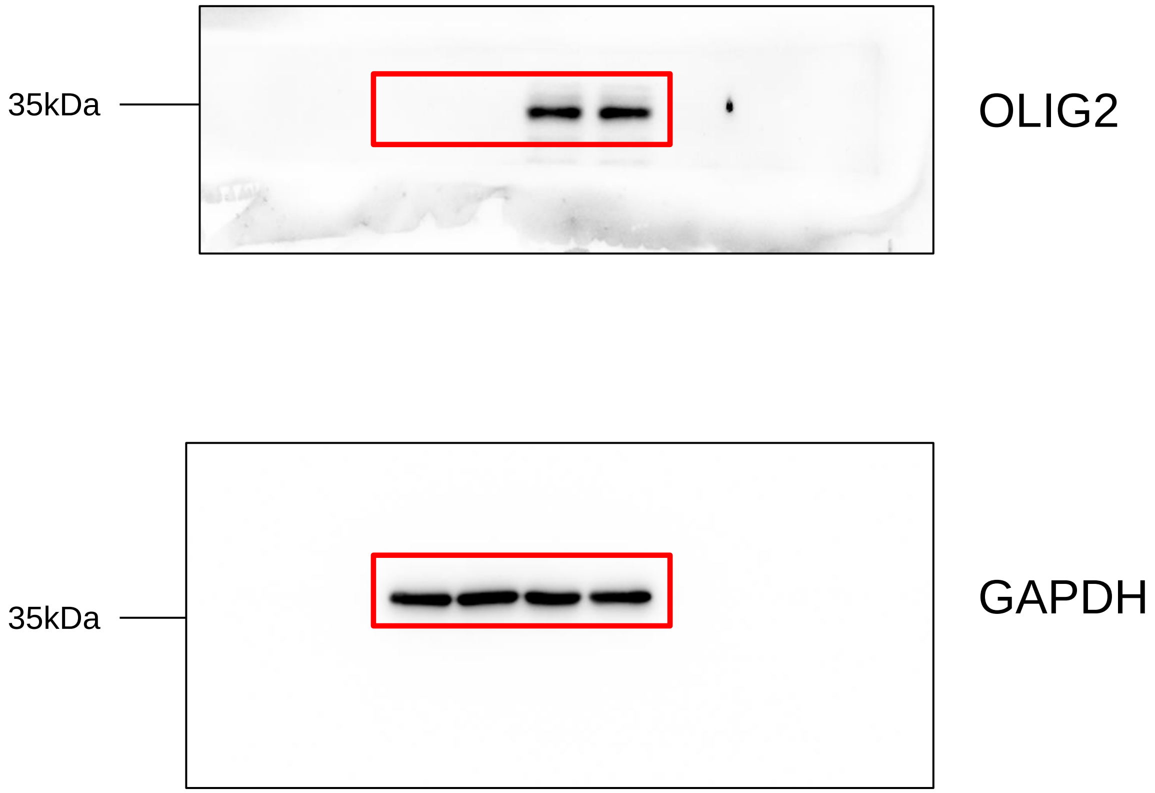

Supplemental Figure 2a

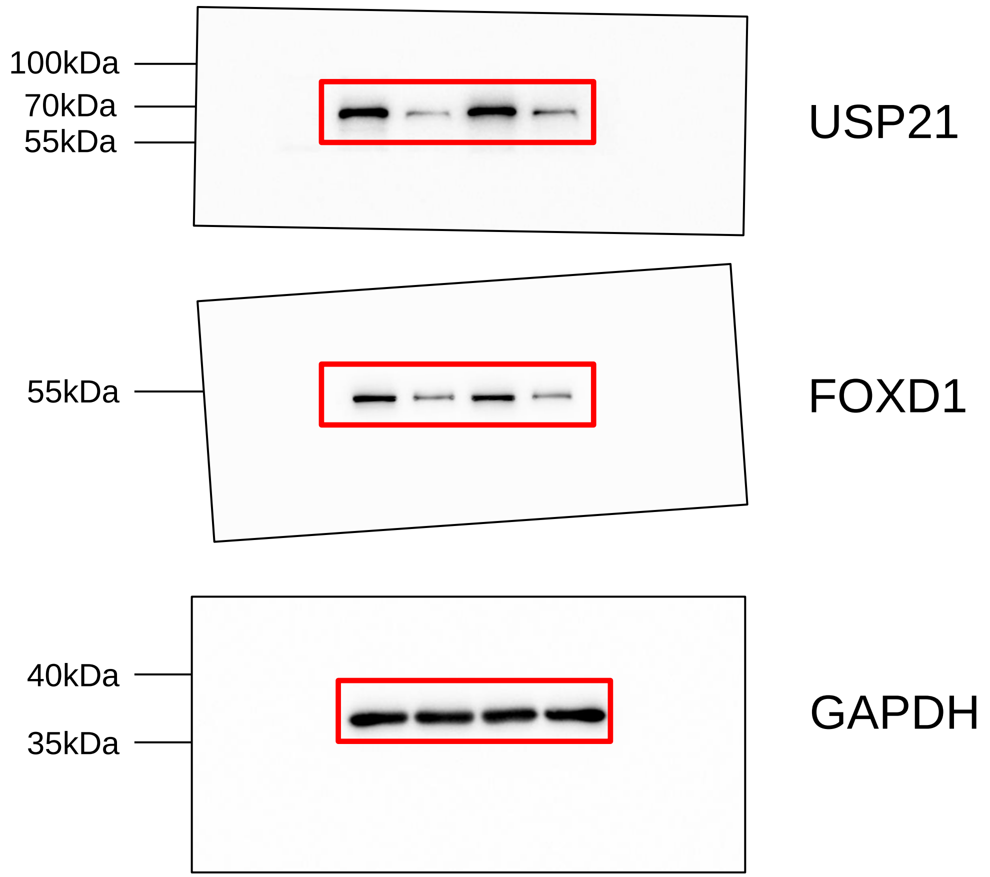

Supplement: Supplementary file 7 — Original Data File [file 41419_2022_5163_MOESM7_ESM.pdf]
