# Supplementary material for: USP21 promotes self-renewal and tumorigenicity of mesenchymal glioblastoma stem cells by deubiquitinating and stabilizing FOXD1
Source: Cell Death Dis. 2022 Aug 16;13(8):712. doi: 10.1038/s41419-022-05163-3 (PMC9381540; doi:10.1038/s41419-022-05163-3)
Supplement: Supplementary file 8 — Reproducibility checklist [file 41419_2022_5163_MOESM8_ESM.pdf]

Corresponding Author Name: Huibo Wang
Manuscript Number: CDDIS-22-0616RR

SPRINGER NATURE

Reporting Summary

Springer Nature wishes to improve the reproducibility of the work that we publish. This checklist is used to ensure good reporting standards and to improve the reproducibility. Please respond completely to all questions relevant to your manuscript. For more information, please read the journal's Guide to Authors.

√ ☐ **Check here to confirm that the following information is available in the Material & Methods section:**

- **The exact sample size (*n*)** for each experimental group/condition, given as a number, not a range
- **A description of the sample collection** allowing the reader to understand whether the samples represent technical or biological replicates (including how many animals, litters, culture, etc.)
- **A statement of how many times the experiment shown was replicated in the laboratory**
- **Definitions of statistical methods and measures**: For small sample sizes (n<5) descriptive statistics are not appropriate, instead plot individual data points
    - Very common tests, such as *t*-test, simple χ^2 tests, Wilcoxon and Mann-Whitney tests, can be unambiguously identified by name only, but more complex techniques should be described in the methods section
    - Are tests one-sided or two-sided?
    - Are there adjustments for multiple comparisons?
    - **Statistical test results**, e.g., ***P* values**
    - Definition of **'center values'** as **median or mean**;
    - Definition of **error bars as s.d. or s.e.m. or c.i.**

Please ensure that the answers to the following questions are reported in the manuscript itself. We encourage you to include a specific subsection in the methods section for statistics, reagents and animal models. Below, provide the page number or section and paragraph number.

Statistics and general methods

Statistics and general methods	Reported in section/paragraph or page #
1. How was the sample size chosen to ensure adequate power to detect a pre-specified effect size? (Give section/paragraph or page #)	In the "Human tissue samples" section on page 5, we calculated the sample size and referenced other literature, and finally selected 138 GBM patients as samples, 91 of which were MES GBM patients
For animal studies, include a statement about sample size estimate even if no statistical methods were used.	In the section "Intracranial xenograft tumor models and treatments" on page 8, we calculated based on the sample size and referenced other literature, and finally selected 10 nude mice in each group for the experiment
2. Describe inclusion/exclusion criteria if samples or animals were excluded from the analysis. Were the criteria pre-established? (Give section/paragraph or page #)	In the section "Human tissue samples" on page 5, we selected ALDH1A3 as the MES GBM marker to screen out MES GBM patients.
3. If a method of randomization was used to determine how samples/animals were allocated to experimental groups and processed, describe it. (Give section/paragraph or page #)	In the section "Intracranial xenograft tumor models and treatments" on page 8, we randomized nude mice for further animal experiments.
For animal studies, include a statement about randomization even if no randomization was used.	A statement about randomization is included in the section "Intracranial xenograft tumor models and treatments" on page 8.

4. If the investigator was blinded to the group allocation during the experiment and/or when assessing the outcome, state the extent of blinding. (Give section/paragraph or page #)	A statement is included in the section "Intracranial xenograft Tumor Models and Treatment" on page 8
For animal studies, include a statement about blinding even if no blinding was done.	A statement about blinding is included in the section "Intracranial xenograft tumor models and treatments" on page 8
5. For every figure, are statistical tests justified as appropriate?	Statistical tests are justified as appropriate in every figure.
Do the data meet the assumptions of the tests (e.g., normal distribution)?	The data meet the assumptions of the tests.
Is there an estimate of variation within each group of data?	There is an estimate of variation within each group of data.
Is the variance similar between the groups that are being statistically compared? (Give section/paragraph or page #)	USP21 maintains the stability of FOXD1 protein" on page 10 ; "Pharmacological inhibition of USP21 by disulfiram promotes FOXD1 ubiquitination and hinders tumor growth" on page 13.

Reagents

Reported in section/paragraph or page #

6. Report the source of antibodies (vendor and catalog number)	Antibody vendors and catalog numbers are detailed in the sections "IB" and "Immunofluorescence (IF) and immunohistochemistry (IHC)" on page 6.
7. Identify the source of cell lines and report if they were recently authenticated (e.g., by STR profiling) and tested for mycoplasma contamination	We describe the origin of our cell lines in the section "Cell cultures" section on page 5, and they have all been tested for mycoplasma contamination.

Animal Models

Reported in section/paragraph or page #

8. Report species, strain, sex and age of animals	In the section"Intracranial xenograft tumor models and treatments" on page 8 we describe the use of 8-weeks-old male
9. For experiments involving live vertebrates, include a statement of compliance with ethical regulations and identify the committee(s) approving the experiments.	In the section "study approval" on page 9 we stated that all animal experiments were conducted with the approval of IACUC of Nanjing Medical University and in conformity with the Guide for the Care and Use of Laboratory Animals (National Academies Press, 2011).

10. We recommend consulting the ARRIVE guidelines (*PLoS Biol.* **8**(6), e1000412,2010) to ensure that other relevant aspects of animal studies are adequately reported.

Human subjects

	Reported in section/paragraph or page #
11. Identify the committee(s) approving the study protocol.	In the section "study approval" on page 9 we state that all tu mor collection and analysis was approved by the IRBs and the ethics committees of Nanjing Medical University and Harbin
12. Include a statement confirming that informed consent was obtained from all subjects.	In the section "study approval" on page 9 we state that inform ed consent was obtained from all individual participants.
13. For publication of patient photos, include a statement confirming that consent to publish was obtained.	We do not publish patient photos in this article.
14. Report the clinical trial registration number (at ClinicalTrials.gov or equivalent).	This article is not a study of a clinical nature.

15. For phase II and III randomized controlled trials, please refer to the CONSORT statement and submit the CONSORT checklist with your submission.

16. For tumor marker prognostic studies, we recommend that you follow the REMARK reporting guidelines.

Data deposition

	Reported in section/paragraph or page #
17. Provide accession codes for deposited data. Data deposition in a public repository is mandatory for:<br>a. Protein, DNA and RNA sequences<br>b. Macromolecular structures<br>c. Crystallographic data for small molecules<br>d. Microarray data	We have stated in the section "datasets" on page 8 that the Microarray data data used is from the CGGA database.

Deposition is strongly recommended for many other datasets for which structured public repositories exist; more details on our data policy are available in the Guide to Authors. We encourage the provision of other source data in supplementary information or in unstructured repositories such as Figshare and Dryad. We encourage publication of Data Descriptors (see Scientific Data) to maximize data reuse.

18. If computer code was used to generate results that are central to the paper's conclusions, include a statement in the Methods section under "**Code availability**" to indicate whether and how the code can be accessed. Include version information as necessary and any restrictions on availability.	The computer code in this article was not used to generate re sults that are central to the paper's conclusions.
